# Revisiting CNC_6_F_5_: The Quest for Isocyanide Ligands with Strong π‑Acceptor Properties Evaluated by Energy Decomposition Analysis

**DOI:** 10.1021/acsomega.5c04766

**Published:** 2025-07-30

**Authors:** Tim-Niclas Streit, Robin Sievers, Malte Sellin, Moritz Malischewski

**Affiliations:** † 9166 Freie Universität Berlin, Institut für Anorganische Chemie, Fabeckstraße 34/36, D-14195 Berlin, Germany; ‡ Institut für Anorganische und Analytische Chemie und Freiburger Materialforschungszentum (FMF), 9174 Albert-Ludwigs-Universität Freiburg, Albertstraße 21, 79104 Freiburg, Germany; § Departement Chemie, Universität Basel, St. Johanns-Ring 19, 4056 Basel, Switzerland

## Abstract

While perfluorinated isocyanide ligands such as CNCF_3_ and CNC_6_F_5_ have been known for decades, their use by organometallic chemists has been limited primarily due to the challenges associated with their cumbersome synthesis. In this study, we present an improved synthetic route to [Cr­(CO)_5_(CNC_6_F_5_)] and present its structural characterization. For a set of isocyanide ligands (CNC_6_H_5_, *p*-CNC_6_H_4_F, CNCH_3_) and their perfluorinated counterparts (CNC_6_F_5_, CNCF_3_), Gibbs energies of complexation have been calculated with regard to a series of isoelectronic metal fragments [V­(CO)_5_]^−^, [Cr­(CO)_5_], [Mn­(CO)_5_]^+^, and [Fe­(CO)_5_]^2+^. Furthermore, the σ-donor and π-acceptor properties of these isocyanide ligands in the resulting complexes were analyzed using the EDA-NOCV method. For completeness, we have also included ligands such as CO, CNH, and N_2_ into the analysis. While only minor differences in complexation energies are observed for the Cr­(CO)_5_ fragment, more pronounced effects have been observed for the charged complexes. Interestingly, perfluorinated isocyanide ligands show in all cases higher complexation energies than the carbonyl ligands, indicating their strong binding to metal centers. Their pronounced σ-donor and π-acceptor abilities reveal their potential suitability to stabilize metal centers in both positive and negative oxidation states.

## Graphical abstract

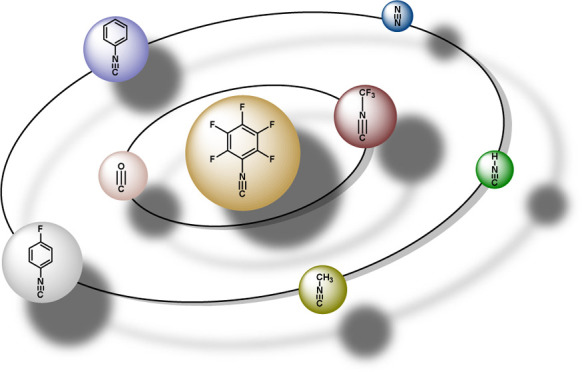

## Introduction

The carbonyl ligand (CO) has played a crucial role in the history of organometallic chemistry, enabling the stabilization of a wide range of homoleptic metal complexes across oxidation states ranging from −IV^1^ to +III^2–4^ while even higher oxidation states up to +VI^5–7^ have been experimentally realized in isolatable heteroleptic carbonyl complexes. Due to its synthetic accessibility and its unusual electronic properties, being a moderate σ-donor as well as an excellent π-acceptor, the CO ligand has proven to be extremely versatile for chemical synthesis, facilitating especially the isolation of low-valent organometallic complexes. As a consequence of its diatomic nature, no modification of the ligand structure and properties is possible, which can be a disadvantage in comparison to other ligand classes.

To address this limitation, the isoelectronic alkyl- and arylisocyanide ligands (CNR) have been extensively studied, as these ligands offer the potential to fine-tune the electronic and steric properties as well as introduce possible chelating designs through the modification of the organic backbone.^8^ Applications of isocyanides and its complexes are diverse, ranging from multicomponent reactions,^9–12^ small molecule activation,^13,14^ modern photosensitizers,^15–18^ supramolecular building blocks,^19–21^ polymers and material sciences,^22–24^ as well as medicinal imaging.^25–28^ In coordination compounds, they typically stabilize transition metal complexes in higher formal oxidation states than CO, extending up to +IV and even +V in homoleptic complexes.^29,30^

The comparison of frontier orbital energies can be used as a first approximation for the classification of ligand properties, although it is associated with significant inaccuracies. In agreement with the expectations, electron-rich isocyanide ligands display higher HOMO energies, indicating increased σ-donor properties. The perfluorination of alkyl or aryl groups in isocyanides, however, lowers their frontier orbital energies, thus making the CNCF_3_ and CNC_6_F_5_ ligands weaker σ-donor and stronger π-acceptor ligands and therefore electronically more similar to the carbonyl ligand than their nonfluorinated analogues.^31–34^ Despite these intriguing properties,^35^ the cumbersome synthesis of perfluorinated ligands has constricted research for several decades to few investigations on this topic.^36–38^ Circumventing the synthetic problem, partial fluorinated isocyanides have proven more accessible in recent years.^39^ In order to validate and quantify the above-mentioned trends, a more sophisticated computational method seemed necessary. This would then also allow well-founded statements about the influence of individual fluorine atoms on the ligand properties (e.g., *p*-CNC_6_H_4_F) in comparison with the nonfluorinated derivatives (Figure 1).^40^ Consequently, EDA-NOCV (energy decomposition analysis of natural orbitals for chemical valence)^41–43^ was chosen as a tool for the comparison of various isocyanide ligands, having the additional advantage of being able to conceptually separate σ-donor from π-acceptor interactions.

**Figure 1:**
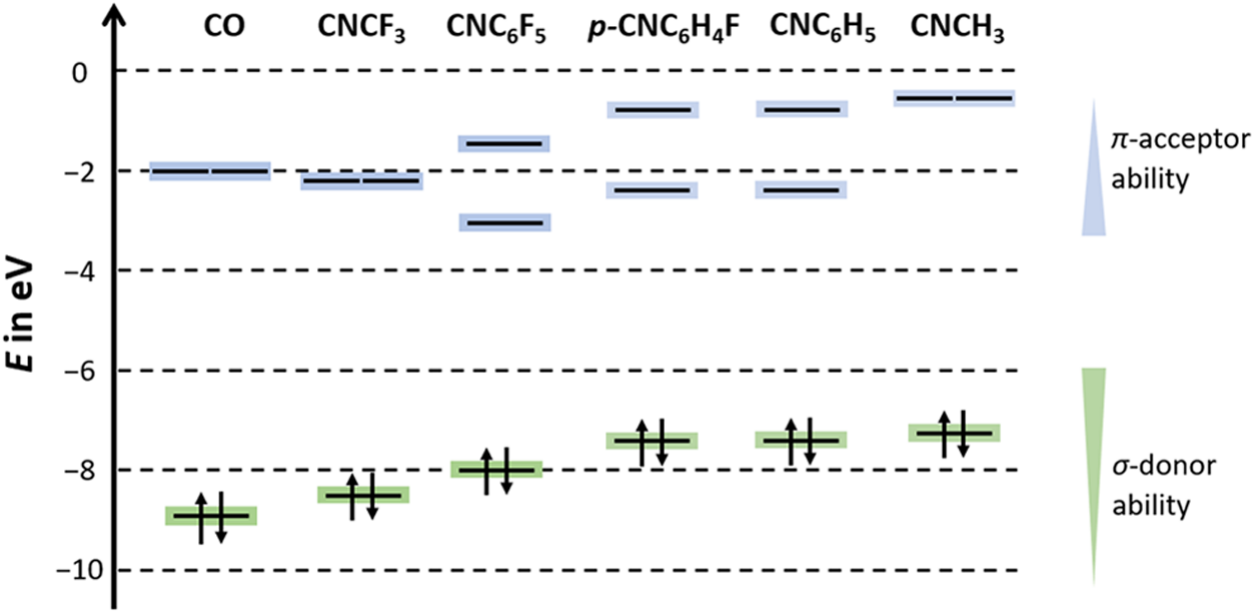
Simplified MO energies in eV and associated donor/acceptor abilities of CO, CNCF_3_, CNC_6_F_5_, p-CNC_6_H_4_F, CNC_6_H_5_, and CNCH_3_ (BP86-D3­(BJ)/def2-TZVPP).

## Results and Discussion

First reports of traces of CNC_6_F_5_ were reported by Haszeldine et al. in 1975,^31^ until Lentz et al. published multiple improved syntheses of the CNC_6_F_5_ ligand a decade later.^33,44^ Hereby, 1,2,3,4,5-pentafluoroaniline is reacted without a solvent at 140 °C with CBr_4_ and AlBr_3_ to give perhalogenated imine C_6_F_5_–N=CBr_2_ in 30% yield. Addition of magnesium in THF leads to reductive dehalogenation. The product is unstable at room temperature and is obtained in low yield as a THF solution upon condensation in a −196 °C cooling trap (Scheme 1). Its subsequent coordination to pentacarbonyl chromium was first accomplished using the labile *cis*-cyclooctene adduct [Cr(CO)_5_(C_8_H_14_)] as a precursor. In contrast, we prepared [Cr­(CO)_5_(CNC_6_F_5_)] by reaction of the more accessible THF adduct [Cr­(CO)_5_(THF)] with CNC_6_F_5_ at −78 °C in 40% yield. This modified procedure was already used for the synthesis of various other pentacarbonyl chromium isocyanide complexes.^39,45^

**Scheme 1:**
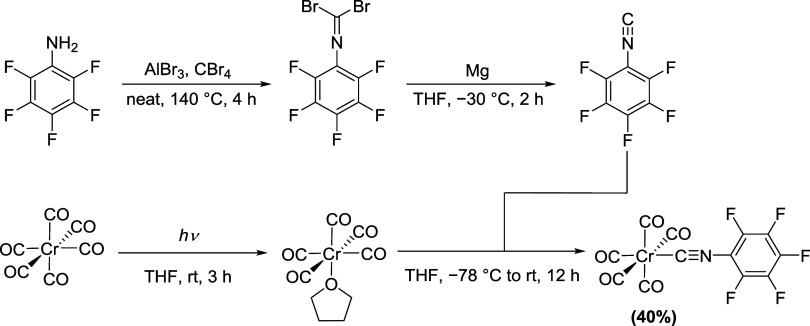
Synthesis of [Cr­(CO)_5_(CNC_6_F_5_)] Starting from C_6_F_5_NH_2_ and [Cr­(CO)_6_]

Single crystals were obtained from solution by slowly cooling [Cr(CO)_5_(CNC_6_F_5_)] in *n-*pentane to −70 °C. [Cr(CO)_5_(CNC_6_F_5_)] crystallizes in monoclinic space group *P*2̅_1_/*c*. It is so far only the third structurally characterized complex with CNC_6_F_5_ as a ligand.^33^ Selected bond lengths are depicted in Table 1, and its structure is shown in Figure 2. The geometric parameters are in excellent agreement with the optimized structure (BP86-D3­(BJ)/def2-TZVPP).

**Figure 2:**
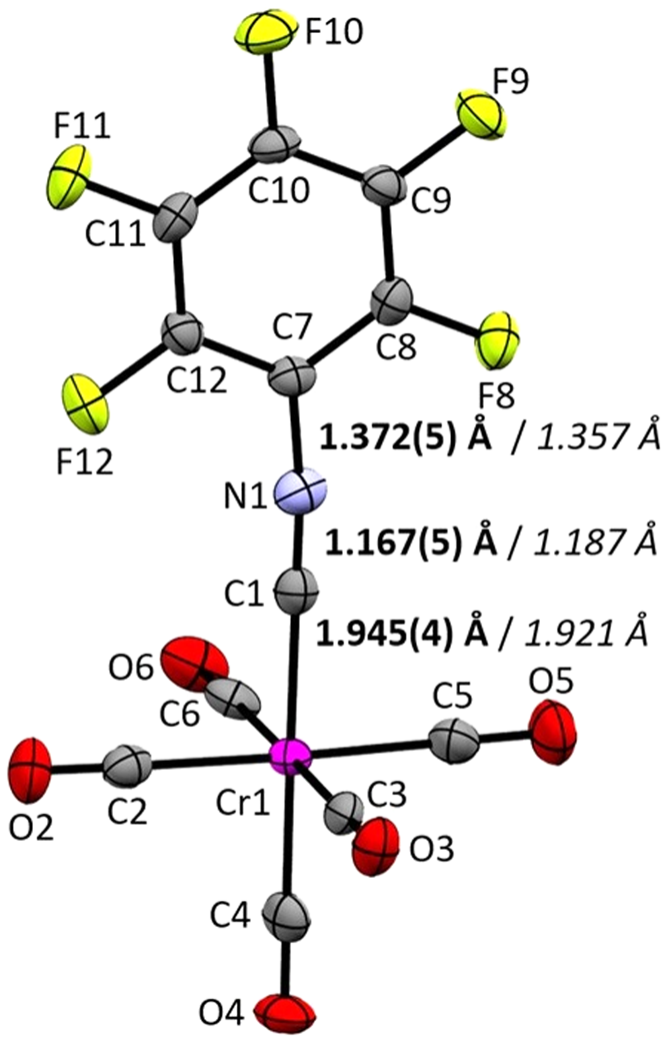
Molecular structure in the solid state of [Cr­(CO)_5_(CNC_6_F_5_)]. Ellipsoids are depicted with 50% probability level. Key experimental bond lengths in bold and calculated bond lengths (BP86­(D3-BJ)/def2-TZVPP) in italic.

**Table 1:** Selected Bond Length in Å of the [Cr­(CO)_5_(CNR)] Complexes

	*d*(Cr–C_iso_)	axial *d*(Cr–C_CO_)	avg. equatorial *d*(Cr–C_CO_)	*d*(N–C_ipso_)	*d*(N≡C)	∠ (CNC)
[Cr(CO)_5_(CNC_6_F_5_)]	1.945(4)	1.892(5)	1.907(5)	1.372(5)	1.167(5)	173.6
[Cr(CO)_5_(CN*p*-FAr^DArF2^)]^a^	1.967(2)	1.894(2)	1.908(6)	1.397(2)	1.162(2)	176.5
[Cr(CO)_5_(CN(3,5-C_6_H_3_(CF_3_)_2_))]^a^	1.960(3)	1.887(3)	1.912(3)	1.393(3)	1.162(4)	177.8
[Cr(CO)_5_(CNXyl)]^a^	1.984(5)	1.881(5)	1.910(2)	1.398(5)	1.162(6)	180.0

a taken from ref 39

In comparison with a series of [Cr­(CO)_5_CNR] complexes with partially fluorinated and nonfluorinated isocyanide ligands reported by Figueroa et al.,^39^ the [Cr­(CO)_5_(CNC_6_F_5_)] complex features the shortest Cr–C distance with 1.945(4) Å. Additionally, the CNC_6_F_5_ ligand exhibits the strongest deviation of a 180° C≡N–C bond angle with 173.6°. This is expected, as an increasing π-acceptor property influences the deviation from linearity,^46^ whereas isocyanide complexes dominated by σ-donation feature almost linear C≡N–C angles exemplified by [Cr­(CO)_5_(CNXyl)]. The structure optimizations on the (BP86-D3­(BJ)/def2-TZVPP) level of theory on the differently charged metal carbonyl fragments [V­(CO)_5_]^−^, [Cr­(CO)_5_], [Mn­(CO)_5_]^+^, and [Fe­(CO)_5_]^2+^ with CO, CNCH_3_, CNC_6_H_5_, *p-*CNC_6_H_4_F, and CNC_6_F_5_ are validating this trend (Figure 3). While the optimized structures for [V­(CO)_5_(CNR)]^−^ (see the SI) demonstrate for each isocyanide ligand a strong degree of deviation from 180° in the C≡N–C angle, the bending of the C≡N–C moiety in [Cr­(CO)_5_CNR] complexes is pronounced only for the perfluorinated ligands. When the metal fragment is very electron-deficient, as is the case for [Fe­(CO)_5_]^2+^, even the strong π-acceptor CNCF_3_ shows a linear C≡N–C arrangement.

**Figure 3:**
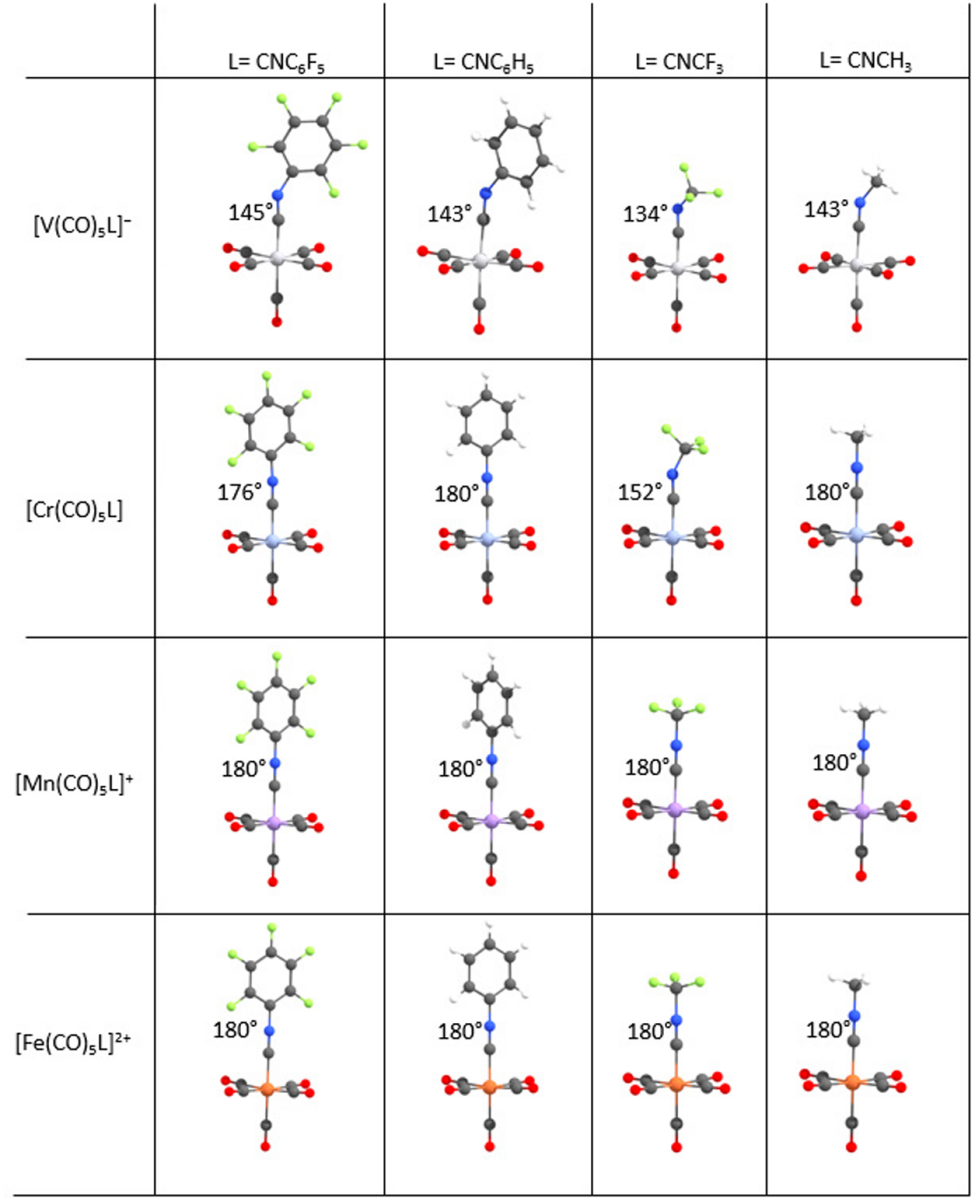
Depiction of ∠ (C≡N–C) angles of L = CNC_6_F_5_, CNC_6_H_5_, CNCF_3_, and CNCH_3_ in differently charged metal carbonyl fragments [V­(CO)_5_]^−^, [Cr­(CO)_5_], [Mn­(CO)_5_]^+^, and [Fe­(CO)_5_]^2+^ (BP86-D3­(BJ)/def2-TZVPP).

In the past, vibrational spectroscopy/force constants, electrochemical measurements, UV–vis spectroscopy, and ^13^C NMR shifts have been used to evaluate the ligand properties of isocyanide ligands.^39,47–49^ Herein, we present a computational approach to investigate various combinations of fluorinated and nonfluorinated isocyanide ligands with a set of square-pyramidal M­(CO)_5_ fragments in oxidation states from −I to +II by analyzing the resulting bonding situations with the EDA-NOCV method. This would, for example, allow a differentiation between σ-donor and π-acceptor contributions, which is typically not possible by vibrational spectroscopy, which will just reveal the net (resulting) effect of both opposing (however synergistic) interactions (see the Supporting Information).

To understand the electronic effects of the perfluorination of isocyanide ligands, the standard Gibbs energy of reaction (Δ_rxn,solv_ *G°*) between isocyanide and related ligands and differently charged metal pentacarbonyl fragments was calculated in a CH_2_Cl_2_ solution (Table 2 and Figure 4).

**Figure 4:**
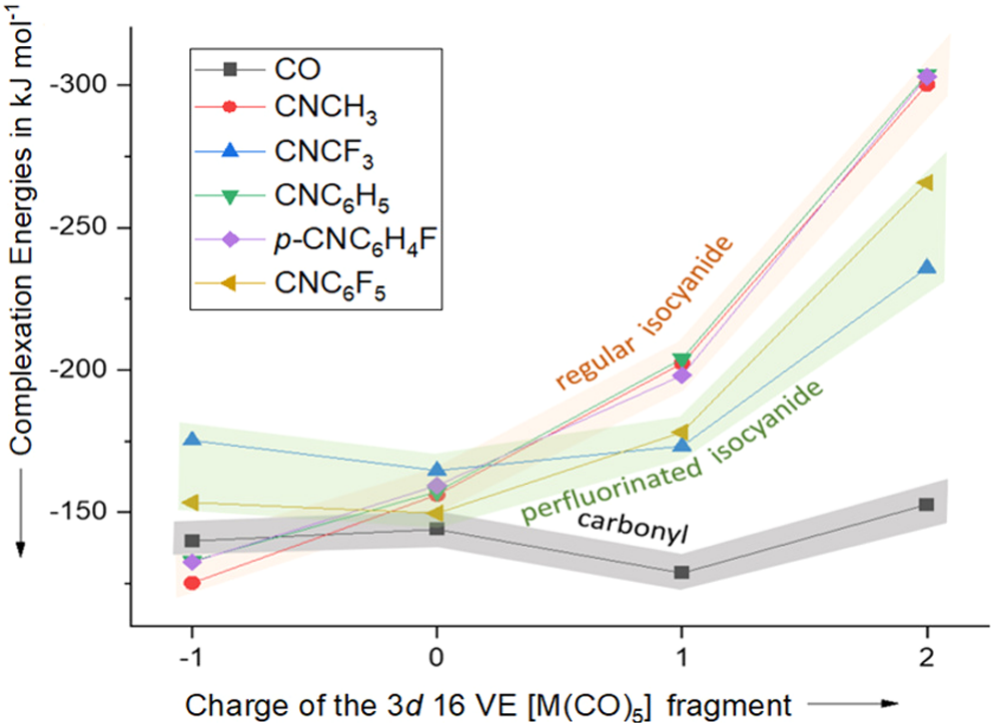
Gibbs complexation energies (Δ_rxn,solv_ *G* ^0^) calculated for CO, CNCH_3_, CNC_6_H_5_, CNC_6_H_4_F, and CNC_6_F_5_ with differently charged metal carbonyl fragments [V­(CO)_5_]^−^, [Cr­(CO)_5_], [Mn­(CO)_5_]^+^, and [Fe­(CO)_5_]^2+^ (BP86-D3­(BJ)/def2-TZVPP). Solvation effects in dichloromethane were calculated using COSMO-RS.

**Table 2:** Gibbs Complexation Energies in kJ mol^–1^ Calculated for CO, CNCH_3_, CNC_6_H_5_, CNC_6_H_4_F, and CNC_6_F_5_ with Differently Charged Metal Carbonyl Fragments [V­(CO)_5_]^−^, [Cr­(CO)_5_], [Mn­(CO)_5_]^+^, and [Fe­(CO)_5_]^2+^ (BP86-D3­(BJ)/def2-TZVPP)^a^

	[V(CO)_5_]^−^	[Cr(CO)_5_]	[Mn(CO)_5_]^+^	[Fe(CO)_5_]^2+^
CO	–140	–144	–128	–152
CNCH_3_	–125	–156	–202	–300
CNCF_3_	–175	–164	–173	–236
CNC_6_H_5_	–132	–157	–203	–304
*p*-CNC_6_H_4_F	–132	–159	–198	–303
CNC_6_F_5_	–153	–150	–178	–265

a Solvation effects in dichloromethane were calculated using COSMO-RS.

Surprisingly, these complexation energies vary only minimally in a range of 10 kJ mol^–1^ for the neutral [Cr­(CO)_5_L] (L = ligand) complexes. Based on the previously discussed MOs, one might have expected a more significant difference between nonfluorinated and fluorinated ligands. In neutral complexes, the data reveal that the improved π-acceptor ability of fluorinated isocyanide ligands is compensated by the weakened σ-donor ability compared to nonfluorinated isocyanides (see EDA-NOCV). Interestingly, the π-acceptor interaction contributes significantly to the overall bonding situation, which is in contrast to previous reports which claimed that the main differences between various isocyanide ligands result from variation in σ-donor ability, whereas d-π* backbonding is negligible.^47^

For anionic carbonyl fragments such as [V­(CO)_5_L]^−^, the difference in Gibbs complexation energies becomes more prominent. CNC_6_F_5_ binds 21 kJ mol^–1^ stronger than CNC_6_H_5_. For the CNCF_3_/CNCH_3_ pair, this effect is even more pronounced (Δ­(Δ_rxn,solv_ *G°*) = 50 kJ mol^–1^), being a consequence of the strongly increased π-acceptor properties of the fluorinated ligand. The complexation energies of the carbonyl ligands lie between those of fluorinated and nonfluorinated ligands with regard to the electron-rich [V­(CO)_5_]^−^ fragment.

In the case of the two investigated cationic metal pentacarbonyl fragments [Mn­(CO)_5_]^+^ and [Fe­(CO)_5_]^2+^, CO binds more weakly than any of the investigated isocyanide ligands (highlighting its poor σ-donor properties). Interestingly, fluorination in *para*-position for the CNC_6_H_5_ shows insignificant changes in orbital energies which results in almost identical complexation energies for CNC_6_H_5_ and *p*-CNC_6_H_4_F. Therefore, *para*-fluorination mainly influences chemical stability and reactivity of the isocyanide, not its complexation energy.^50^ This is likely due to the strong +M effect of fluorine at the *para*-position. For cationic metal carbonyls such as [Mn­(CO)_5_]^+^, complexation energies of nonfluorinated ligands CNCH_3_/CNC_6_H_5_ outperform CNCF_3_/CNC_6_F_5_ with Δ­(Δ_rxn,solv_ *G°*) = 29/25 kJ mol^–1^ respectively. This discrepancy increases further with [Fe­(CO)_5_]^2+^ to Δ­(Δ_rxn,solv_ *G°*) = 65/38 kJ mol^–1^, presumably originating from energetically higher-lying HOMOs of the nonfluorinated ligands. Due to the strong electron-withdrawing effect of the CO ligands, the Fe­(II) fragment is not particularly electron-rich. The use of stronger σ-donor ligands (e.g. phosphines) should therefore increase the stability of complexes with fluorinated isocyanide ligands, even in cationic complexes.

To investigate the binding interactions of the [Cr­(CO)_5_L] complexes in further detail, we performed EDA-NOCV calculations using the Cr­(CO)_5_ and the ligand fragments from the optimized structures on the (BP86-D3­(BJ)/def2-TZVPP) level of theory.^51–53^ The orbital interactions are as expected from the Dewar-Chatt-Duncanson^41^ model consisting of one σ-donation and two π-backdonation interactions for all the investigated ligands. Figure 5 shows the shapes of the deformation densities of the NOCVs and the corresponding participating symmetrized fragment orbitals (SFOs) that are exemplary for the combination of [Cr­(CO)_5_] with CNC_6_F_5_.

**Figure 5:**
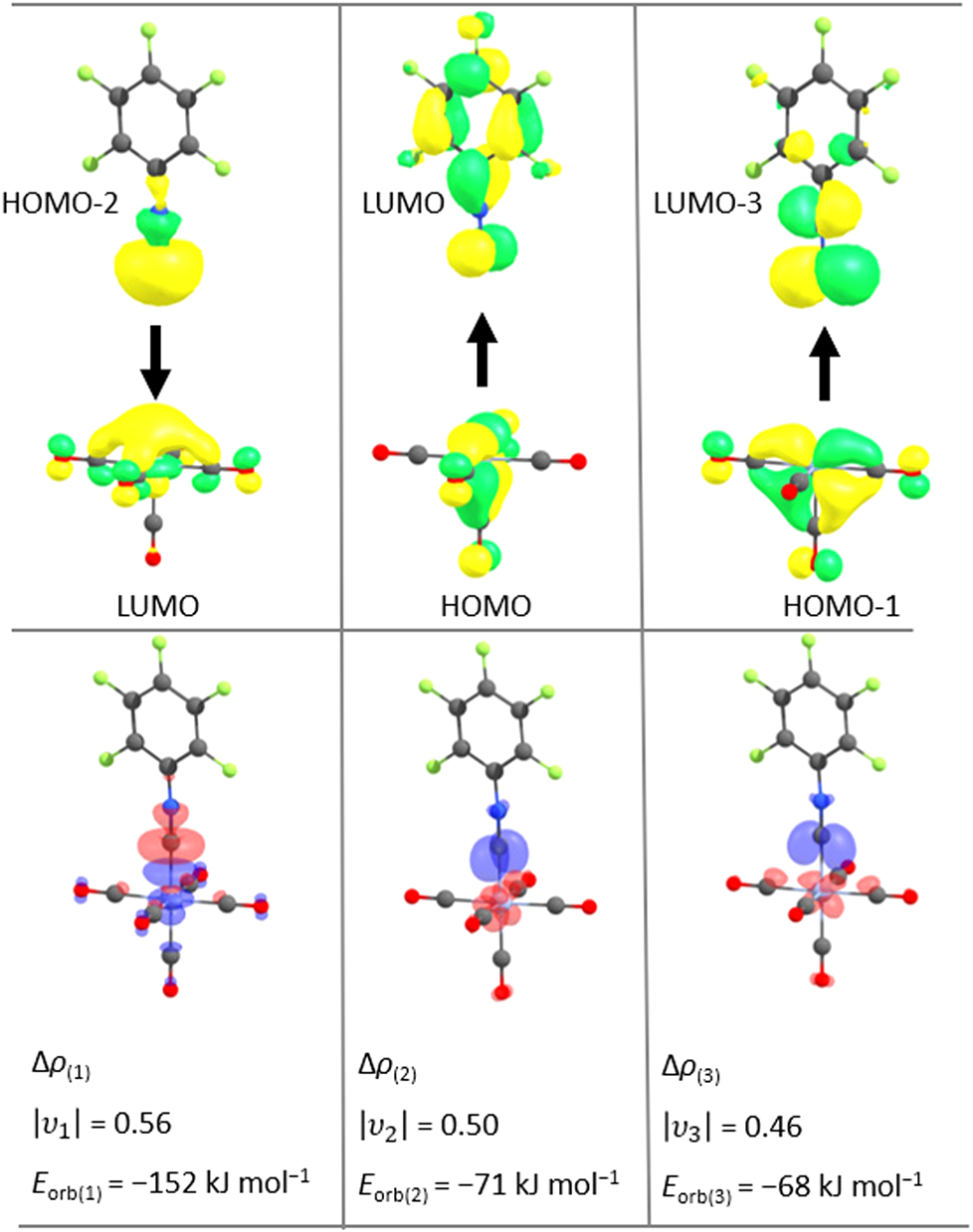
Deformation density plots for [Cr­(CO)_5_(CNC_6_F_5_)] of NOCVs (natural orbitals for chemical valence) 1–3 and most participating SFOs (symmetrized fragment orbitals) with Δρ (difference in electron density) (BP86-B3BJ/def2-TZV2P). Charge flow direction in the deformation densities is reddish to blue. Isodensity surfaces of the SFOs and deformation densities are plotted on the isosurface values of 0.05 and 0.003 *e* B^3–^, respectively.

Furthermore, we calculated the series of anionic, neutral, and cationic carbonyl complex fragments [V­(CO)_5_]^−^, [Cr­(CO)_5_], [Mn­(CO)_5_]^+^, [Fe­(CO)_5_]^2+^, and the ligands CO, CNH, CNCH_3_, CNCF_3_, CNC_6_H_5_, *p-*CNC_6_H_4_F, and CNC_6_F_5_ (see Tables 3–6). In all calculated metal fragments, CNCF_3_, CNC_6_H_5_, *p*-CNC_6_H_4_F, and CNC_6_F_5_ show increased Δ*E*_int_ compared to CO, as the overall charge of the metal fragment can be stabilized more efficiently by the ligand. As expected, the perfluorinated isocyanide ligand CNC_6_F_5_ shows 41 kJ mol^–1^ stronger Δ*E*_orb,π_ (acceptor) interactions than CNC_6_H_5_ for the [V­(CO)_5_]^−^ reference system, while the analogous difference between CNCF_3_ and CNCH_3_ is even more pronounced (Δ*E*_orb,π_ = 117 kJ mol^–1^). In general, perfluorinated isocyanide ligands always demonstrate higher Δ*E*_orb,π_ contributions to their respective Δ*E*_orb_ than nonfluorinated isocyanide ligands, resulting from their lower-lying LUMOs visualized in Figure 1 and consequently increased π-acceptor properties. This is also shown in the neutral [Cr­(CO)_5_] fragment in which CNC_6_F_5_ even exhibits the highest Δ*E*_int_ = −229 kJ mol^–1^ of all investigated ligands, even though CNC_6_H_5_ exhibits a very similar interaction energy.

**Table 3:** Summary of the Results of the EDA-NOCV Calculations between the [V­(CO)_5_]^−^ Fragment and a Variation of Ligands in kJ mol^–1^ ^a^

						NOCV stabilization interaction	
[V(CO)_5_]^−^	Δ*E*_int_	Δ*E*_Pauli_	Δ*E*_elstat_	Δ*E*_disp_	Δ*E*_orb_	Δ*E*_orb σ_	Δ*E*_orb π_ (2x π-acceptor)
CNC_6_F_5_	–274	511	–364	–30	–391	–123/31%	–250/64%
*p*-CNC_6_H_4_F	–252	506	–373	–31	–355	–122/34%	–214/60%
CNC_6_H_5_	–243	502	–366	–30	–349	–122/35%	–209/60%
CNCF_3_	–304	506	–363	–26	–422	–122/29%	–285/68%
CNCH_3_	–207	454	–338	–24	–296	–115/39%	–168/57%
CNH	–230	465	–344	–23	–329	–117/36%	–200/61%
CO	–224	403	–291	–20	–316	–102/32%	–203/64%
N_2_	–140	243	–151	–19	–213	–63/30%	–138/65%

a Percentages refer to the ratio of σ- and π-contribution of Δ*E*_orb_

**4:** Summary of the Results of the EDA-NOCV Calculations between the Cr­(CO)_5_ Fragment and a Variation of Ligands in kJ mol^–1^ ^a^

						NOCV stabilization interaction	
[Cr(CO)_5_]	Δ*E*_int_	Δ*E*_Pauli_	Δ*E*_elstat_	Δ*E*_disp_	Δ*E*_orb_	Δ*E*_orb σ_	Δ*E*_orb π_ (2x π-acceptor)
CNC_6_F_5_	–229	495	–392	–28	–304	–152/50%	–140/46%
*p*-CNC_6_H_4_F	–226	486	–402	–27	–283	–152/54%	–119/42%
CNC_6_H_5_	–227	486	–404	–27	–282	–152/54%	–118/42%
CNCF_3_	–228	486	–376	–26	–312	–148/47%	–155/49%
CNCH_3_	–220	443	–383	–25	–255	–144/56%	–100/39%
CNH	–220	477	–389	–23	–285	–150/53%	–124/44%
CO	–211	446	–326	–21	–310	–138/45%	–162/52%
N_2_	–120	234	–153	–20	–180	–78/43%	–92/51%

a Percentages refer to the ratio of *σ*- and π-contribution of Δ*E*_orb_

**5:** Summary of the Results of the EDA-NOCV Calculations between the [Mn­(CO)_5_]^+^ Fragment and a Variation of Ligands in kJ mol^–1^ ^a^

						NOCV stabilization interaction	
[Mn(CO)_5_]^+^	Δ*E*_int_	Δ*E*_Pauli_	Δ*E*_elstat_	Δ*E*_disp_	Δ*E*_orb_	Δ*E*_orb σ_	Δ*E*_orb π_ (2x π-acceptor)
CNC_6_F_5_	–284	477	–420	–16	–326	–195/60%	–111/34%
*p*-CNC_6_H_4_F	–303	480	–447	–15	–321	–198/62%	–102/32%
CNC_6_H_5_	–308	482	–454	–15	–321	–199/62%	–101/31%
CNCF_3_	–254	487	–407	–14	–320	–192/60%	–113/35%
CNCH_3_	–297	482	–461	–13	–305	–197/65%	–91/30%
CNH	–272	487	–439	–12	–307	–194/63%	–100/32%
CO	–207	467	–343	–10	–320	–180/56%	–128/40%
N_2_	–118	247	–162	–9	–193	–105/54%	–77/40%

a Percentages refer to the ratio of *σ*- and π-contribution of Δ*E*_orb_

**Table 6:** Summary of the Results of the EDA-NOCV Calculations between the [Fe­(CO)_5_]^2+^ Fragment and a Variation of Ligands in kJ mol^–1^ ^a^

						NOCV stabilization interaction	
[Fe(CO)_5_]^2+^	Δ*E*_int_	Δ*E*_Pauli_	Δ*E*_elstat_	Δ*E*_disp_	Δ*E*_orb_	Δ*E*_orb σ_	Δ*E*_orb π_ (2x π-acceptor)
CNC_6_F_5_	–437	481	–457	–18	–443	–259/59%	–146/33%
*p*-CNC_6_H_4_F	–473	495	–500	–18	–450	–267/59%	–146/32%
CNC_6_H_5_	–479	496	–510	–17	–447	–267/60%	–142/32%
CNCF_3_	–372	471	–446	–15	–382	–252/66%	–104/27%
CNCH_3_	–435	489	–519	–15	–390	–260/67%	–105/27%
CNH	–375	479	–477	–14	–363	–249/68%	–96/26%
CO	–243	436	–339	–12	–339	–222/65%	–102/30%
N_2_	–155	234	–222	–11	–222	–134/60%	–75/34%

a Percentages refer to the ratio of *σ*- and π-contribution of Δ*E*_orb_

In cationic complexes such as [Fe­(CO)_5_]^2+^, Δ*E*_orb_ interactions are mainly dominated by its σ-contribution. However, the differences between fluorinated and nonfluorinated isocyanides are surprisingly small, exemplified by the difference in CNCH_3_ (Δ*E*_orb,σ_ −260 kJ mol^–1^) to CNCF_3_ (Δ*E*_orb,σ_ = −252 kJ mol^–1^). The notably lower Δ*E*_int_ of −435 kJ mol^–1^ for CNCH_3_ to CNCF_3_ (Δ*E*_int_ = −372 kJ) can only be explained by the more favorable electrostatic contributions Δ*E*_elstat_ in the case of CNCH_3_. Interestingly, CNC_6_F_5_ exhibits the highest contribution of the Δ*E*_orb,π_ interaction of all investigated ligands in this scenario, leading to an almost identical Δ*E*_int_ of −437 kJ mol^–1^ compared with CNCH_3_ of −435 kJ mol^–1^. CNC_6_H_5_ and partially fluorinated *p*-CNC_6_H_4_F exhibit almost exclusively similar Δ*E*_orb_ and Δ*E*_int_ values in all investigated cases, leading to identical bonding situations, in which *para*-fluorination merely influences the coordination ability.^39,50^

## Conclusions

In summary, CNCF_3_ and CNC_6_F_5_ are strong π-acceptor ligands, which correlates also with their tendency to deviate from linear C≡N–C geometries. The interaction energies resulting from the EDA-NOCV are validating the increased strong π-acceptor properties of the isocyanides arising from the perfluorination. Interestingly, perfluorination does not lead to a strong decrease of σ-donor ability, but generally, CNCF_3_ is a weaker σ-donor than CNC_6_F_5_. Nevertheless, cationic complexes in higher oxidation states are better stabilized using the stronger σ-donors CNCH_3_, CNC_6_H_5_, and *p*-CNC_6_H_4_F. The latter two exhibit close to identical interaction energies, exemplifying that only perfluorination leads to distinctly different electronic properties. Overall, the good σ-donor and strong π-acceptor properties of perfluorinated isocyanide ligands make these ligands important alternatives to the ubiquitous CO ligand. With these results, it is feasible that CNCF_3_ and CNC_6_F_5_ could stabilize electron-rich metal centers as effectively as CO, possibly also extending metal isocyanide chemistry in negative oxidation states. However, since they also stabilize metal centers in positive oxidation states, they should also be excellently suited for applications in catalysis where the high degree of fluorination could facilitate their recovery using perfluorinated solvents. Their combination of significant σ-donor and strong π-acceptor properties should provide metal complexes with large ligand field splitting energies, which could display attractive photophysical properties. Furthermore, non-radiative decay pathways from excited states could be avoided through the absence of carbon-hydrogen bonds.

## Experimental Section

### Pentacarbonyl-1,2,3,4,5-pentafluorophenylisocyanide-chromium­(0)

**Caution!** Cr­(CO)_6_ is toxic, and benzene C_6_H_6_ is carcinogenic. Ultraviolet (UV) lamps emit radiation that can damage the skin and eyes. Therefore, the irradiation reaction has to be carried out in a shielded fume hood.

Following a modified procedure,^39,45^ a 500 mL Schlenk flask was filled with Cr­(CO)_6_ (1.69 g, 7.70 mmol, 10 equiv), dissolved in THF (350 mL) subjected to three freeze–pump–thaw cycles. The solution was warmed to room temperature and irradiated for 3 h using a mercury UV lamp. A solution of CNC_6_F_5_ (150 mg, 0.77 mmol, 1 equiv) in THF (5 mL) was added to the orange solution and stirred for 2 h at −78 °C and subsequently warmed to room temperature over 12 h. The yellow solution was evaporated to dryness. Unreacted Cr­(CO)_6_ was recovered using sublimation (4 × 10^–2^ mbar, 40 °C) for 6 h. The resulting residue was extracted using C_6_H_6_ and filtered under an argon atmosphere. The pale yellow filtrate was evaporated to dryness, and the residue was dissolved in *n*-pentane (10 mL) and stored in a −70 °C freezer. The crude product was purified by column chromatography (*n*-pentane). Pale yellow crystals of [Cr­(CO)_5_(CNC_6_F_5_)] (120 mg, 0.29 mmol) were obtained in a yield of 40%. The analytical data is in accordance with the literature.^38^

^**19**^ **F-NMR** (377 MHz, CDCl_3_, rt) δ (ppm) = −143.4 (m, ortho, 2F), −151.7 (m, para, 1F), −159.5 (m, meta, 2F).

^**13**^ **C­{** ^**19**^ **F}-NMR** (101 MHz, CDCl_3_, rt) δ [ppm] = 214.7 (s, CO_trans_), 213.4 (s, CO_cis_), 193.8 (s, CN), 143.8, 141.6, 138.5, 106.1 (s, C_ipso_).

**HRMS** (EI-TOF, positive) *m*/*z* for [C_12_F_5_O_5_NCr]^+^ calculated: 384.9102; measured: 384.9137.

**FT-IR** (ATR) *ṽ* [cm^–1^] = 2965 (w), 2138 (m), 2051 (s), 2008 (w), 1918 (vs), 1514 (s), 1463 (m), 1359 (w), 1322 (w), 1253 (m), 1139 (m), 1027 (m), 990 (s), 804 (m), 634 (s).

**Raman** *ṽ* [cm^–1^] = 2138 (m), 2042 (s), 2000 (s), 1654 (m), 1518 (w), 1459 (w), 1324 (w), 566 (m), 464 (w), 442 (w), 390 (m), 112 (m).

## Supplementary Material





## Notes

Crystallographic data and computational details (PDF)
P21c_full (CIF)
