# Supplementary material for: Revisiting CNC6F5: The Quest for Isocyanide Ligands with Strong π‑Acceptor Properties Evaluated by Energy Decomposition Analysis
Source: ACS Omega. 2025 Jul 30;10(31):35095–102. doi: 10.1021/acsomega.5c04766 (PMC12355232; doi:10.1021/acsomega.5c04766)
Supplement: Supplementary file 1 [file ao5c04766_si_001.pdf]

Supporting Information on

Revisiting CNC_6F_5: The quest for isocyanide ligands with strong π-acceptor properties evaluated by energy decomposition analysis

Tim-Niclas Streit, Robin Sievers, Malte Sellin and Moritz Malischewski**

Tim-Niclas Streit, Freie Universität Berlin, Institut für Anorganische Chemie, Fabeckstraße 34/36 D-14195 Berlin, Germany

Robin Sievers, Freie Universität Berlin, Institut für Anorganische Chemie, Fabeckstraße 34/36 D-14195 Berlin, Germany

Dr. Malte Sellin, Institut für Anorganische und Analytische Chemie and Freiburger Materialforschungszentum (FMF), Albert-Ludwigs-Universität Freiburg, Albertstraße 21, 79104 Freiburg, Germany, malte.sellin@unibas.ch

Dr. Moritz Malischewski, Freie Universität Berlin, Institut für Anorganische Chemie, Fabeckstraße 34/36, D-14195 Berlin, Germany, moritz.malischewski@fu-berlin.de

Table of Contents

1. General Procedures 2
2. Crystallographic Data 4
3. Analytical Data 5
   3.1 NMR Spectroscopy 5
   3.2 IR Spectroscopy 6
   3.3 Raman Spectroscopy 7

Figure S4. Experimental Raman spectrum (rt) 7

   3.4 Mass Spectrometry 8

4. DFT Calculations 9
   4.1 DFT Optimized Structures 9
5. References 78

1. General Procedures

All reactions and workups were performed in previously heated glassware under an atmosphere of argon using standard Schlenk techniques[1] and an oil pump vacuum of 10^{-3} mbar. Room temperature (rt) refers to 25 °C. The addition of liquid reagents and solvents was done by using threefold argon-flushed disposable syringes and septa, while solids were added in argon stream. Anhydrous THF was freshly distilled under potassium and stored over activated 3 Å molecular sieves. Deuterated solvents $CDCl_3$ and THF-d_8 were used as purchased and stored over activated 3 Å molecular sieves. Hexacarbonyl chromium was purchased from Sigma-Aldrich and used with no further purification.

Nuclear magnetic resonance (NMR) spectroscopy

NMR spectroscopy was measured on a JEOL ECX 400 (400 MHz) in the reported deuterated solvents THF-d_8. The ^{13}C-NMR spectra are calibrated on the respective resonance signals of $CDCl_3$ (δ = 77.16 ppm relative to tetramethylsilane). The ^{19}F-spectra are internally reference to $CFCl_3$. The given multiplicities are phenomenological, thus the actual appearance of the signals is stated and not the theoretically expected one. The following abbreviations were used and analogously combined to designate multiplicities: s (singlet), d (doublet), t (triplet), q (quartet), m (multiplet), m_c (centrosymmetric multiplet). Evaluation of spectra was performed with Mestrelab Research MNova 7.[2]

Infrared (IR) spectroscopy

IR spectroscopy was measured on a FT (Fourier transformation) Nicolet. The sample was directly measured by ATR (attenuated total reflection) technique. Characteristic absorptions are given in wavenumbers $\tilde{\nu}$ [cm^{-1}] and intensities are stated as vs (very strong), s (strong), m (medium) and w (weak).

Raman spectroscopy

Raman spectra were recorded on a Bruker MultiRAM II equipped with a low-temperature Ge detector (1064 nm). Characteristic absorptions are given in wavenumbers $\tilde{\nu}$ [cm^{-1}] and intensities are stated as vs (very strong), s (strong), m (medium) and w (weak).

High resolution mass spectrometry (HRMS)

HRMS was recorded using a Varian MAT 711 spectrometer by electron impact ionization (EI) at the department of mass spectrometry at the Freie Universität Berlin. A detailed listing of fragmentation is dispensed, instead the molecular ion peak or a characteristic fragment peak is stated.

Single-crystal X-ray diffraction (XRD)

X-Ray data were collected on a BRUKER D8 Venture system. Data were collected at 100(2) K using graphite monochromated Mo K_α radiation (λ_α = 0.71073 Å). The strategy for the data collection was evaluated by using the Smart software. The data were collected by the standard "ψ-ω scan techniques" and were scaled and reduced using Saint+software. The structure was solved by using Olex2,[3] the structure was solved with the XT[4] structure solution program using Intrinsic Phasing and refined with the XL refinement package[5,6] using Least Squares minimization. Bond length and angles were measured with Diamond Crystal and Molecular Structure Visualization Version 4.6.2.[7] Drawings were generated with POV-Ray.[8]

Density functional theory (DFT) calculations

Geometry optimizations were performed with the TURBOMOLE software[9] (v7.2 or v7.5) using the DFT functionals BP86 with the def2-TZVPP[10] basis set, the resolution-of-identity (RI) approximation,[11] dispersion correction (D3BJ),[12] a fine integration grid (m4) and the default SCF convergence criteria (10^{-6} a.u.). All structures were checked for proper spin occupancies and imaginary frequencies with the integrated *EIGER* and *AOFORCE*[13] modules. Gibbs free energies of solvation were calculated with the COSMO-RS model[14] at the BP86(D3)/def-TZ VP level of theory using the fine cavity construction algorithm ($cosmo_isorad) and the CosmoThermX software. EDA-NOCV calculations were carried out using the BP86(D3-BJ)/def2-TZVPP optimized structures on the BP86(D3-BJ)/TZ2P level of theory with the AMS software package. The figures were generated using ChemCraft.[15]

2. Crystallographic Data

Table S 1. Crystallographic data of $[Cr(CO)_5(CNC_6F_5)]$.

Empirical formula	$C_{12}CrF_5NO_5$
CCDC	2419003
Formula weight	385.13
Temperature/K	100.0
Crystal system	monoclinic
Space group	$P2_1/c$
a/Å	12.0759(6)
b/Å	10.6308(5)
c/Å	11.8263(5)
α/°	90
β/°	117.338(2)
γ/°	90
Volume/$Å^3$	1348.65(11)
Z	4
$\rho_{calc}g/cm^3$	1.897
μ/mm^{-1}	0.937
F(000)	752.0
Crystal size/mm^3	0.1 × 0.1 × 0.1
Radiation	MoKα (λ = 0.71073)
2Θ range for data collection/°	5.394 to 50.716
Index ranges	-14 ≤ h ≤ 14, -12 ≤ k ≤ 12, -14 ≤ l ≤ 14
Reflections collected	26458
Independent reflections	2472 [R_{int} = 0.0350, R_{sigma} = 0.0156]
Data/restraints/parameters	2472/0/217
Goodness-of-fit on F^2	1.158
Final R indexes [I>=2σ (I)]	R_1 = 0.0534, wR_2 = 0.1258
Final R indexes [all data]	R_1 = 0.0655, wR_2 = 0.1399
Largest diff. peak/hole / e $Å^{-3}$	1.22/-0.64

3. Analytical Data

3.1 NMR Spectroscopy

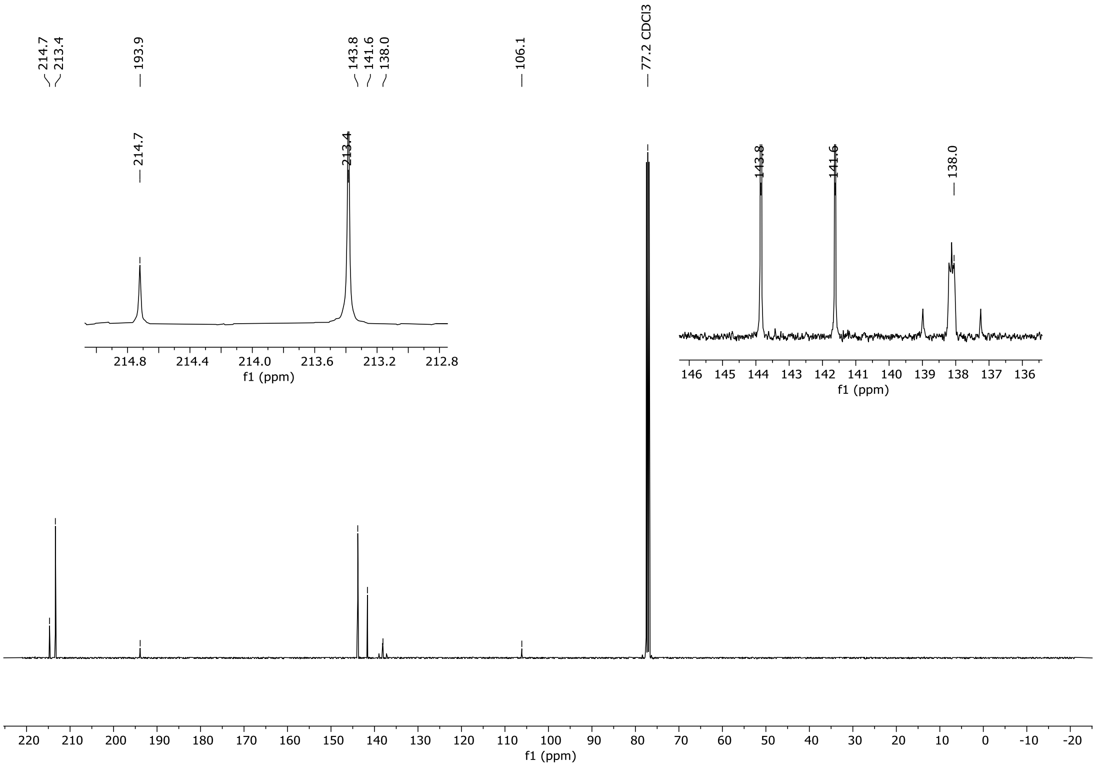

Figure S1. $^{13}C\{^{19}F\}$**-NMR** (101 MHz, $CDCl_3$, rt) spectrum of[$Cr(CO)_5(CNC_6F_5)$].

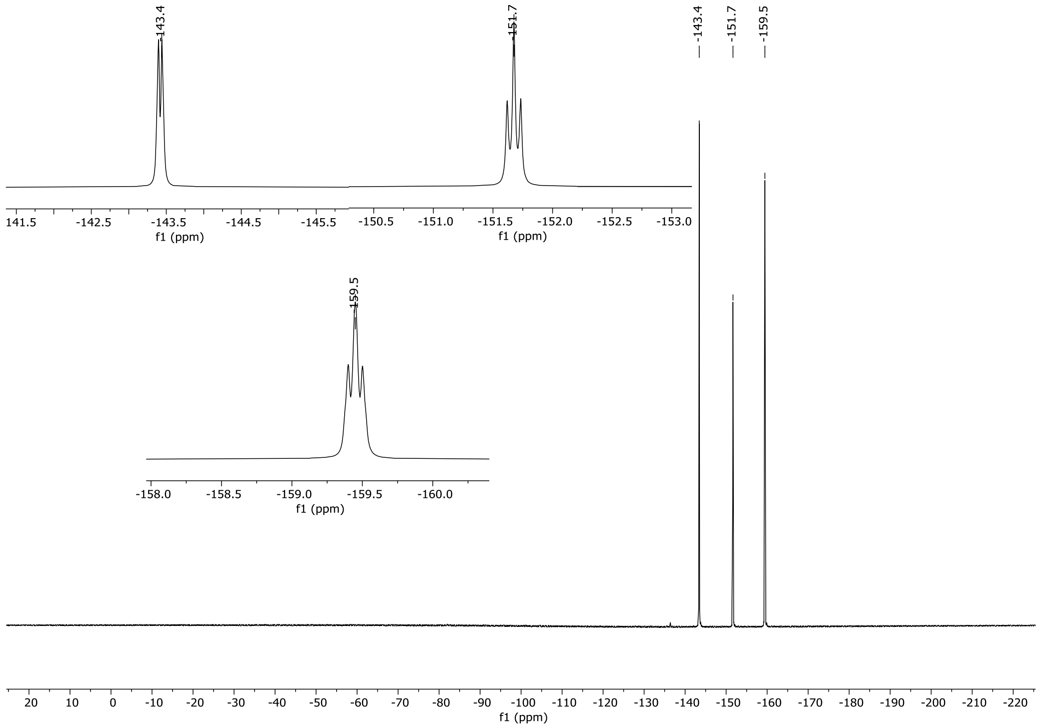

Figure S2. ^{19}F**-NMR** (377 MHz, $CDCl_3$, rt) spectrum of [$Cr(CO)_5(CNC_6F_5)$].

3.2 IR Spectroscopy

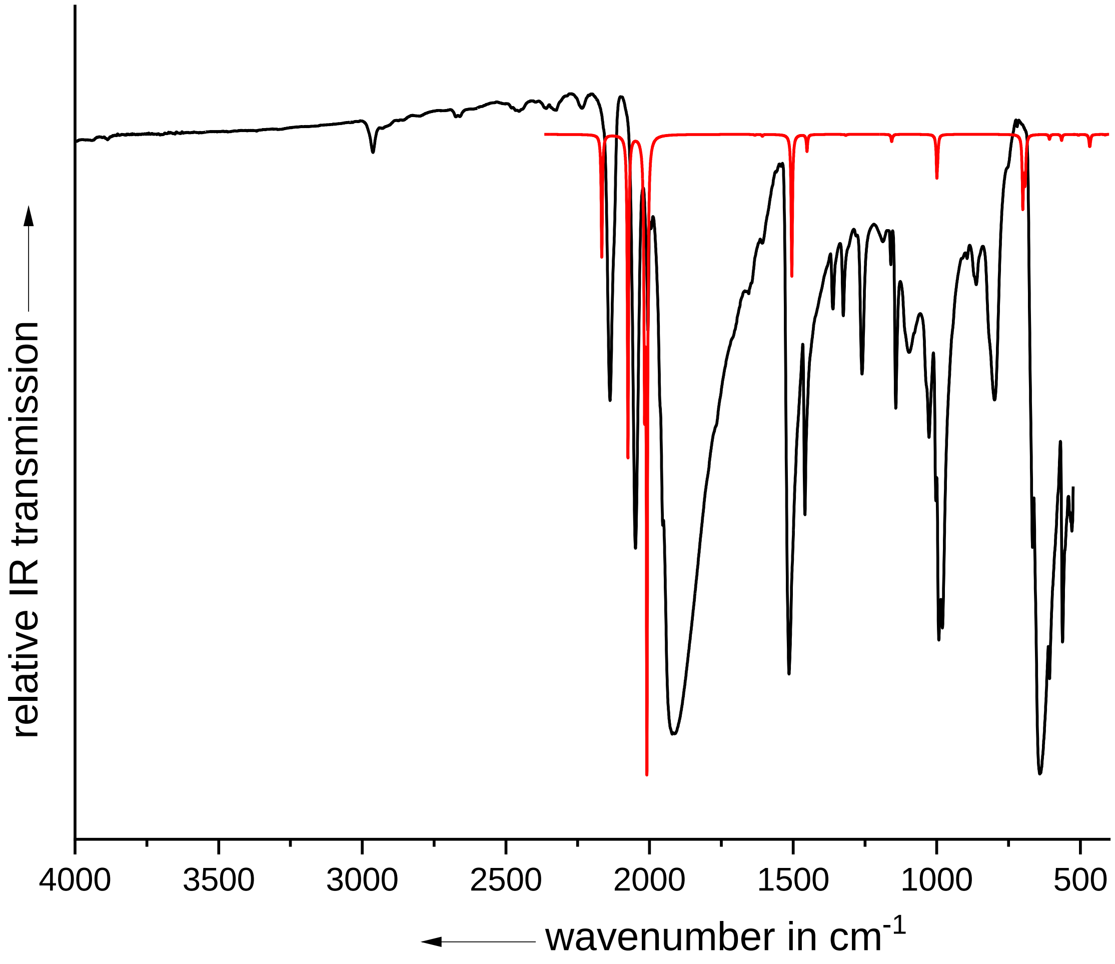

Figure S3. Experimental IR (ATR, rt) spectrum of [Cr(CO)$_5$(CNC$_6$F$_5$)] (black line) in comparison to the BP86(D3-BJ)/def2-TZVPP calculated IR spectrum scaled by the factor 1.013.[16]

3.3 Raman Spectroscopy

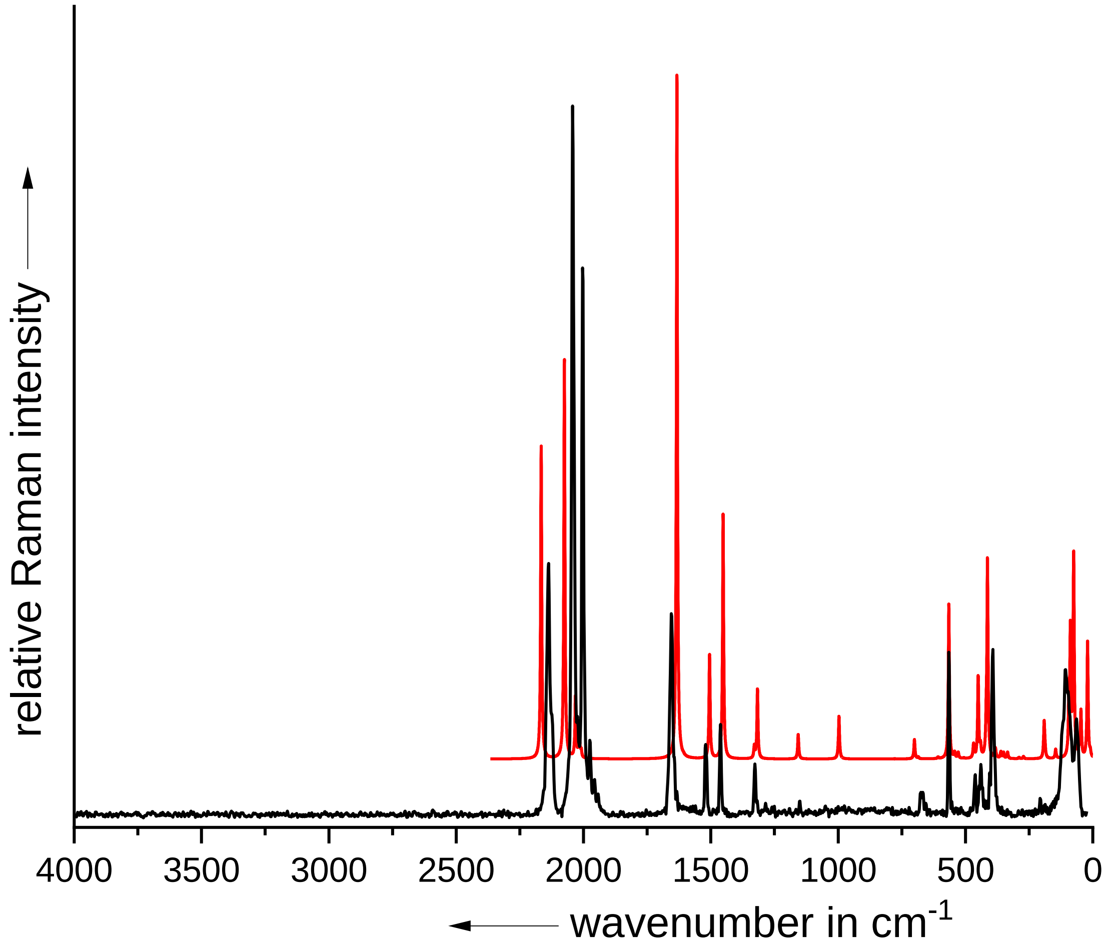

Figure S4. Experimental Raman spectrum (rt) of [Cr(CO)$_5$(CNC$_6$F$_5$)] (black line) in comparison to the BP86(D3-BJ)/def2-TZVPP calculated Raman spectrum scaled by the factor 1.013.[15]

3.4 Mass Spectrometry

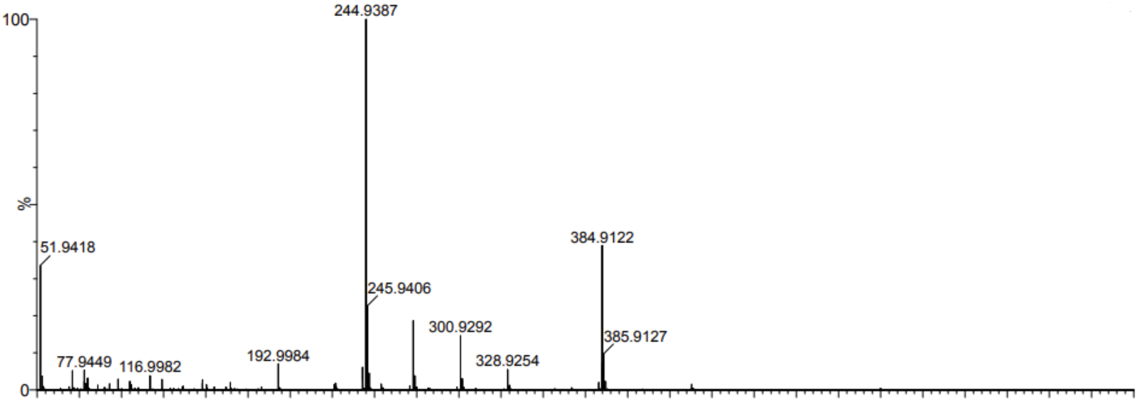

Figure S5. Electron ionization spectrum of [$Cr(CO)_5(CNC_6F_5)$].

4. DFT Calculations

4.1 DFT Optimized Structures

$[V(CO)_5]^-$

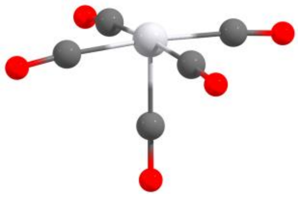

```
Method: (RI-)BP86(D3BJ)/def2-TZVPP
Symmetry: c4v

Cartesian coordinates in Ångström:
V    -0.0000000    0.0000000    0.5633467
O     2.2001767   -2.2001767    0.3275669
O     2.2001767    2.2001767    0.3275669
O     0.0000000    0.0000000   -2.4919230
O    -2.2001767   -2.2001767    0.3275669
O    -2.2001767    2.2001767    0.3275669
C     0.0000000    0.0000000   -1.3125533
C     1.3776473    1.3776473    0.4827155
C    -1.3776473   -1.3776473    0.4827155
C     1.3776473   -1.3776473    0.4827155
C    -1.3776473    1.3776473    0.4827155

SCF energy GEOOPT =  -1511.295920627 H
ZPE =   102.6 kJ/mol
FREEH energy =   134.40 kJ/mol
FREEH entropy =   0.46682 kJ/mol/K
mode     symmetry     wave number   IR intensity    selection rules
#                      cm**(-1)        km/mol          IR      RAMAN
    7        b2            29.82         0.00000         NO       YES
    8        e             53.48         0.40458         YES      YES
    9        e             53.48         0.40458         YES      YES
   10        b1            73.26         0.00000         NO       YES
   11        a1            79.17         0.57885         YES      YES
   12        e             83.92         0.93282         YES      YES
   13        e             83.92         0.93282         YES      YES
   14        a2           362.68         0.00000         NO       NO
   15        e            362.71         0.01396         YES      YES
   16        e            362.71         0.01396         YES      YES
   17        a1           394.14         0.60988         YES      YES
   18        b2           399.96         0.00000         NO       YES
   19        e            462.62        14.83066         YES      YES
   20        e            462.62        14.83066         YES      YES
   21        a1           490.00         0.01831         YES      YES
   22        b2           491.85         0.00000         NO       YES
   23        b1           507.95         0.00000         NO       YES
   24        e            545.36         6.39266         YES      YES
   25        e            545.36         6.39266         YES      YES
   26        e            638.81        36.80597         YES      YES
   27        e            638.81        36.80597         YES      YES
   28        a1           668.65        43.98320         YES      YES
   29        a1          1834.79       920.73348         YES      YES
   30        e           1845.44      2544.22746         YES      YES
   31        e           1845.44      2544.22746         YES      YES
   32        b2          1870.22         0.00000         NO       YES
   33        a1          1967.76        45.27266         YES      YES
```

$[V(CO)_5(N_2)]^-$

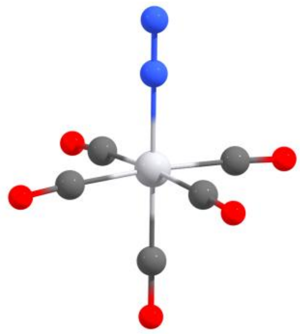

Method: (RI-)BP86(D3BJ)/def2-TZVPP
Symmetry: c4v

Cartesian coordinates in Ångström:

V	0.0000000	0.0000000	0.0748361
O	2.2054124	-2.2054124	-0.1144021
O	2.2054124	2.2054124	-0.1144021
O	0.0000000	0.0000000	-3.0247896
O	-2.2054124	-2.2054124	-0.1144021
N	0.0000000	0.0000000	3.2039129
O	-2.2054124	2.2054124	-0.1144021
C	-0.0000000	0.0000000	-1.8526508
N	0.0000000	0.0000000	2.0770080
C	1.3823913	1.3823913	-0.0051770
C	-1.3823913	-1.3823913	-0.0051770
C	1.3823913	-1.3823913	-0.0051770
C	-1.3823913	1.3823913	-0.0051770

SCF energy GEOOPT = -1620.925347090 H
ZPE = 124.3 kJ/mol
FREEH energy = 162.55 kJ/mol
FREEH entropy = 0.51535 kJ/mol/K

mode #	symmetry	wave number cm**(-1)	IR intensity km/mol	selection rules IR	RAMAN
7	b2	43.31	0.00000	NO	YES
8	e	47.73	0.04741	YES	YES
9	e	47.73	0.04741	YES	YES
10	b1	74.27	0.00000	NO	YES
11	a1	76.22	0.81221	YES	YES
12	e	78.14	0.17001	YES	YES
13	e	78.14	0.17001	YES	YES
14	e	84.97	0.46807	YES	YES
15	e	84.97	0.46807	YES	YES
16	e	352.67	0.00719	YES	YES
17	e	352.67	0.00719	YES	YES
18	a1	358.73	6.73782	YES	YES
19	a2	360.38	0.00000	NO	NO
20	a1	389.88	0.00293	YES	YES
21	b2	396.13	0.00000	NO	YES
22	e	451.28	2.41743	YES	YES
23	e	451.28	2.41743	YES	YES
24	a1	463.43	6.70320	YES	YES
25	e	473.54	7.33410	YES	YES
26	e	473.54	7.33410	YES	YES
27	b2	501.00	0.00000	NO	YES
28	b1	506.46	0.00000	NO	YES
29	e	522.00	1.53445	YES	YES
30	e	522.00	1.53445	YES	YES
31	e	659.09	100.77029	YES	YES
32	e	659.09	100.77029	YES	YES
33	a1	662.76	110.77546	YES	YES
34	a1	1873.88	979.06174	YES	YES
35	e	1874.89	2345.83870	YES	YES
36	e	1874.89	2345.83870	YES	YES
37	b2	1894.98	0.00000	NO	YES
38	a1	1969.01	571.26786	YES	YES
39	a1	2117.58	567.60402	YES	YES

[V(CO)$_6$]$^{-}$

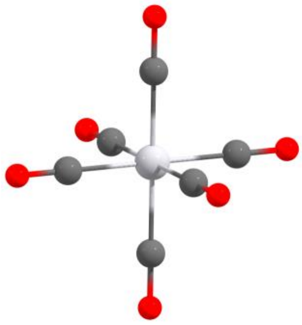

Method: (RI-)BP86(D3BJ)/def2-TZVPP
Symmetry: oh

Cartesian coordinates in Ångström:

V	0.0000000	0.0000000	-0.0000000
O	-0.0000000	3.1231012	-0.0000000
O	-0.0000000	0.0000000	3.1231012
O	-3.1231012	-0.0000000	-0.0000000
O	-0.0000000	0.0000000	-3.1231012
O	3.1231012	0.0000000	0.0000000
O	0.0000000	-3.1231012	-0.0000000
C	-1.9546846	-0.0000000	0.0000000
C	1.9546846	0.0000000	0.0000000
C	0.0000000	0.0000000	1.9546846
C	-0.0000000	-0.0000000	-1.9546846
C	-0.0000000	1.9546846	-0.0000000
C	0.0000000	-1.9546846	0.0000000

SCF energy GEOOPT = -1624.740764984 H
ZPE = 124.2 kJ/mol
FREEH energy = 162.27 kJ/mol
FREEH entropy = 0.50364 kJ/mol/K

mode	symmetry	wave number	IR intensity	selection rules	
#		cm**(-1)	km/mol	IR	RAMAN
13	t1u	75.20	0.53632	YES	NO
14	t1u	75.20	0.53632	YES	NO
15	t1u	75.20	0.53632	YES	NO
16	t1g	364.26	0.00000	NO	NO
17	t1g	364.26	0.00000	NO	NO
18	t1g	364.26	0.00000	NO	NO
19	a1g	387.82	0.00000	NO	YES
20	eg	398.60	0.00000	NO	YES
21	eg	398.60	0.00000	NO	YES
22	t1u	465.53	5.43388	YES	NO
23	t1u	465.53	5.43388	YES	NO
24	t1u	465.53	5.43388	YES	NO
25	t2u	503.68	0.00000	NO	NO
26	t2u	503.68	0.00000	NO	NO
27	t2u	503.68	0.00000	NO	NO
28	t2g	510.68	0.00000	NO	YES
29	t2g	510.68	0.00000	NO	YES
30	t2g	510.68	0.00000	NO	YES
31	t1u	671.98	120.18043	YES	NO
32	t1u	671.98	120.18043	YES	NO
33	t1u	671.98	120.18043	YES	NO
34	t1u	1881.41	2303.49280	YES	NO
35	t1u	1881.41	2303.49280	YES	NO
36	t1u	1881.41	2303.49280	YES	NO
37	eg	1899.38	0.00000	NO	YES
38	eg	1899.38	0.00000	NO	YES
39	a1g	2005.79	0.00000	NO	YES

$[V(CO)_5(CNH)]^-$

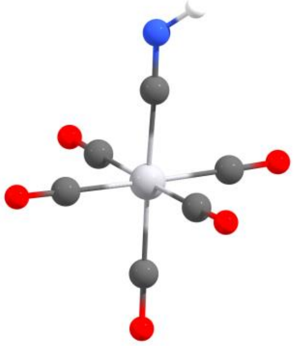

Method: (RI-)BP86(D3BJ)/def2-TZVPP
Symmetry: c1

Cartesian coordinates in Ångström:

V	0.0494443	-0.2948642	0.0409174
O	-3.0723107	-0.2821818	0.1012588
O	0.0648445	-0.1874092	-3.0796121
N	0.0005811	2.8789276	0.0880759
O	0.0375752	-0.3106726	3.1658353
O	0.0545386	-3.4215822	-0.0500913
O	3.1727171	-0.2116709	-0.0127299
C	-0.0033708	1.6595534	0.0473743
C	0.0554213	-2.2526660	-0.0133130
C	0.0564930	-0.2368128	-1.9107750
C	0.0476534	-0.3119099	1.9975136
C	-1.9027544	-0.2961526	0.0758653
C	2.0052221	-0.2501369	0.0126973
H	-0.5660547	3.5175782	-0.4630168

SCF energy GEOOPT = -1604.811466058 H
ZPE = 148.1 kJ/mol
FREEH energy = 188.88 kJ/mol
FREEH entropy = 0.55197 kJ/mol/K

mode #	symmetry	wave number cm**(-1)	IR intensity km/mol	selection rules IR	RAMAN
7	a	36.62	0.09022	YES	YES
8	a	38.91	0.32668	YES	YES
9	a	40.87	3.36570	YES	YES
10	a	65.95	16.97011	YES	YES
11	a	68.01	0.66517	YES	YES
12	a	71.45	1.05666	YES	YES
13	a	72.83	0.04710	YES	YES
14	a	73.39	0.21969	YES	YES
15	a	74.51	0.16617	YES	YES
16	a	97.74	49.73050	YES	YES
17	a	349.01	20.21061	YES	YES
18	a	357.77	0.32976	YES	YES
19	a	363.94	0.00258	YES	YES
20	a	388.31	0.19817	YES	YES
21	a	399.20	0.43949	YES	YES
22	a	399.63	4.72089	YES	YES
23	a	446.85	75.59115	YES	YES
24	a	460.76	5.88603	YES	YES
25	a	464.54	14.86669	YES	YES
26	a	482.11	10.81535	YES	YES
27	a	488.40	0.68770	YES	YES
28	a	499.59	0.63606	YES	YES
29	a	503.61	0.64521	YES	YES
30	a	509.31	1.05213	YES	YES
31	a	512.21	0.00049	YES	YES
32	a	627.96	297.21944	YES	YES
33	a	664.08	109.30774	YES	YES
34	a	667.31	139.96315	YES	YES
35	a	714.69	219.91872	YES	YES
36	a	1798.73	1567.36316	YES	YES
37	a	1875.27	2238.41024	YES	YES
38	a	1875.50	2198.79623	YES	YES
39	a	1886.22	824.85595	YES	YES
40	a	1895.25	122.18641	YES	YES

41	a	1989.89	68.22635	YES	YES
42	a	3500.08	13.93362	YES	YES

[V(CO)$_5$(CNCH$_3$)]$^-$

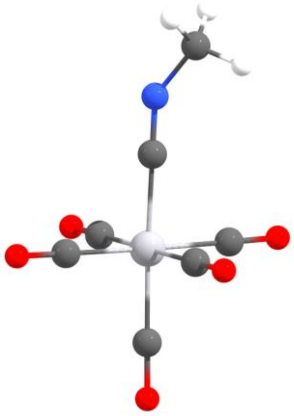

Method: (RI-)BP86(D3BJ)/def2-TZVPP
Symmetry: c1

Cartesian coordinates in Ångström:

V	0.7708975	-0.3094302	-0.7423469
H	-1.9194069	0.2534694	2.7781993
H	-3.4010528	0.7985788	1.9398194
H	-2.2588835	1.9971382	2.5812798
O	3.1659622	-1.7038708	-2.1809690
O	1.4569789	2.4122451	-2.1177037
O	-1.1612114	-1.0507478	-3.0833748
O	0.0764885	-3.0297911	0.6251098
O	2.6490143	0.4740271	1.6259683
N	-1.6399081	1.0197901	0.8469797
C	-0.4344806	-0.7712554	-2.2105629
C	1.9467445	0.1772287	0.7373731
C	1.1991883	1.3924814	-1.6072652
C	0.3380251	-2.0090318	0.1147253
C	2.2670805	-1.1773071	-1.6462830
C	-0.7234297	0.5141040	0.2453122
C	-2.3319966	1.0123814	2.0937487

SCF energy GEOOPT = -1644.137279992 H
ZPE = 222.0 kJ/mol
FREEH energy = 267.29 kJ/mol
FREEH entropy = 0.60935 kJ/mol/K

mode #	symmetry	wave number cm**(-1)	IR intensity km/mol	selection rules IR	RAMAN
7	a	14.49	2.40182	YES	YES
8	a	30.12	0.35976	YES	YES
9	a	30.26	1.78763	YES	YES
10	a	36.79	0.00565	YES	YES
11	a	46.56	0.14350	YES	YES
12	a	53.82	2.20287	YES	YES
13	a	67.72	2.47956	YES	YES
14	a	72.49	0.06744	YES	YES
15	a	73.94	0.19049	YES	YES
16	a	74.17	0.20841	YES	YES
17	a	83.65	1.00124	YES	YES
18	a	121.26	38.53089	YES	YES
19	a	349.39	10.74405	YES	YES
20	a	352.89	0.05999	YES	YES
21	a	363.93	0.00229	YES	YES
22	a	377.77	12.77302	YES	YES
23	a	392.20	0.22631	YES	YES
24	a	398.71	0.04166	YES	YES
25	a	454.48	15.19341	YES	YES
26	a	456.92	2.06662	YES	YES
27	a	466.94	5.23799	YES	YES
28	a	481.97	3.53229	YES	YES
29	a	500.64	13.25363	YES	YES
30	a	503.66	1.53642	YES	YES
31	a	507.52	11.25769	YES	YES

32	a	512.34	12.13997	YES	YES
33	a	514.04	0.03471	YES	YES
34	a	661.49	111.12407	YES	YES
35	a	663.08	142.25888	YES	YES
36	a	668.61	93.08966	YES	YES
37	a	998.79	42.91484	YES	YES
38	a	1089.79	12.75242	YES	YES
39	a	1100.92	0.54501	YES	YES
40	a	1391.61	176.15094	YES	YES
41	a	1437.82	1.37682	YES	YES
42	a	1444.47	7.28400	YES	YES
43	a	1868.11	2247.53567	YES	YES
44	a	1869.68	2173.64328	YES	YES
45	a	1876.02	1554.86361	YES	YES
46	a	1889.51	107.62142	YES	YES
47	a	1918.52	1443.83489	YES	YES
48	a	2006.21	74.69170	YES	YES
49	a	2922.17	220.82224	YES	YES
50	a	2979.53	33.38563	YES	YES
51	a	2982.87	31.01718	YES	YES

$[V(CO)_5(CNCF_3)]^-$

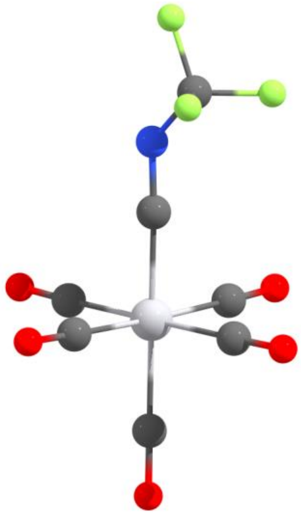

Method: (RI-)BP86(D3BJ)/def2-TZVPP
Symmetry: c1

Cartesian coordinates in Ångström:

V	0.7477929	-0.4645511	-0.6607576
F	-1.8386564	-0.4974223	3.1240613
F	-3.3180107	-0.8010881	1.5344808
F	-3.2359450	1.1086068	2.6050471
O	3.1093228	-1.4814877	-2.4648999
O	1.2430216	2.4748727	-1.6001099
O	-1.3206115	-0.8538187	-2.9675792
O	0.1947646	-3.3883366	0.3019397
O	2.6862064	-0.0317402	1.7481079
N	-1.5742040	0.6460033	1.1442390
C	-0.5383286	-0.7126023	-2.1142575
C	1.9725839	-0.1975704	0.8407665
C	1.0731987	1.3732515	-1.2606351
C	0.4081123	-2.3021800	-0.0610871
C	2.2315707	-1.1071603	-1.7969523
C	-0.6715640	0.1637932	0.4488629
C	-2.4519939	0.1256304	2.0642033

SCF energy GEOOPT = -1942.037151294 H
ZPE = 162.8 kJ/mol
FREEH energy = 211.95 kJ/mol
FREEH entropy = 0.65317 kJ/mol/K

mode	symmetry	wave number	IR intensity	selection rules	
#		cm**(-1)	km/mol	IR	RAMAN
7	a	6.83	0.03067	YES	YES
8	a	15.66	0.05168	YES	YES
9	a	22.94	0.02817	YES	YES
10	a	33.70	0.00119	YES	YES
11	a	42.36	0.00438	YES	YES
12	a	53.31	0.41285	YES	YES
13	a	66.41	1.13414	YES	YES
14	a	67.78	0.06861	YES	YES
15	a	72.03	0.31062	YES	YES
16	a	73.42	0.33840	YES	YES
17	a	76.70	0.05098	YES	YES
18	a	96.97	0.76058	YES	YES
19	a	284.11	0.56185	YES	YES
20	a	344.79	0.11253	YES	YES
21	a	353.73	0.14576	YES	YES
22	a	361.42	0.00017	YES	YES
23	a	387.68	2.27690	YES	YES
24	a	395.53	0.01437	YES	YES
25	a	396.96	0.01862	YES	YES
26	a	415.30	12.31597	YES	YES
27	a	440.85	3.29752	YES	YES
28	a	460.58	5.77819	YES	YES

29	a	468.63	4.30199	YES	YES
30	a	487.36	0.08977	YES	YES
31	a	492.45	0.85130	YES	YES
32	a	497.41	0.34425	YES	YES
33	a	502.74	0.04964	YES	YES
34	a	507.75	0.16052	YES	YES
35	a	513.78	1.70128	YES	YES
36	a	563.27	0.18786	YES	YES
37	a	596.58	71.34123	YES	YES
38	a	605.91	12.45567	YES	YES
39	a	664.74	114.82585	YES	YES
40	a	665.37	164.57114	YES	YES
41	a	686.02	97.61560	YES	YES
42	a	808.31	366.81316	YES	YES
43	a	1021.51	258.41764	YES	YES
44	a	1053.17	261.49675	YES	YES
45	a	1205.71	1172.66953	YES	YES
46	a	1775.15	2747.38977	YES	YES
47	a	1894.99	2188.35891	YES	YES
48	a	1902.90	2145.46880	YES	YES
49	a	1911.06	496.70461	YES	YES
50	a	1917.11	240.73069	YES	YES
51	a	2008.86	59.10972	YES	YES

$[V(CO)_5(CNC_6H_5)]^-$

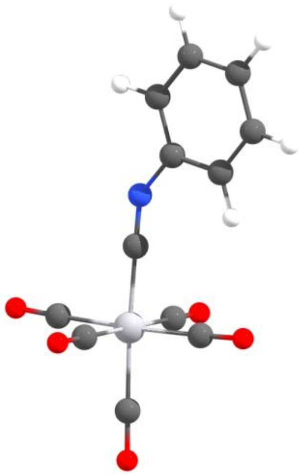

Method: (RI-)BP86(D3BJ)/def2-TZVPP
Symmetry: c1

Cartesian coordinates in Ångström:

V	2.0541622	-0.9687237	-1.4487739
H	-5.0316207	-0.0059050	2.2119818
H	-3.2989869	0.9393403	3.7404330
H	-0.8937710	0.8901650	3.0851307
H	-1.9297598	-1.0446682	-0.6151690
H	-4.3331896	-0.9981927	0.0296663
O	3.8211234	-1.9083549	-3.8572912
O	1.5521241	1.7559997	-2.8937255
O	-0.4780672	-2.1745631	-2.8147051
O	2.5936655	-3.6839882	0.0017582
O	4.4719810	0.3033922	0.0729157
N	0.0697793	-0.0958654	0.8492752
C	0.4838719	-1.7302517	-2.3185427
C	3.5795244	-0.1757637	-0.5060038
C	1.7414946	0.7379175	-2.3548263
C	2.3927606	-2.6697939	-0.5400328
C	3.1708194	-1.5544702	-2.9543724
C	0.8432020	-0.4312767	-0.0300477
C	-1.2590462	-0.0778446	1.1966248
C	-2.2437534	-0.6129321	0.3343700
C	-1.6593023	0.4802762	2.4269568
C	-3.0056962	0.5032343	2.7841694
C	-3.5844249	-0.5821085	0.7055825
C	-3.9789802	-0.0259727	1.9292460

SCF energy GEOOPT = -1835.983603369 H
ZPE = 357.9 kJ/mol
FREEH energy = 409.99 kJ/mol
FREEH entropy = 0.66042 kJ/mol/K

mode	symmetry	wave number	IR intensity	selection rules	
#		cm**(-1)	km/mol	IR	RAMAN
7	a	13.18	0.13138	YES	YES
8	a	24.91	0.37707	YES	YES
9	a	26.00	0.02470	YES	YES
10	a	39.14	0.11805	YES	YES
11	a	47.32	1.64876	YES	YES
12	a	49.15	0.01681	YES	YES
13	a	62.79	2.49474	YES	YES
14	a	72.70	0.13566	YES	YES
15	a	73.83	0.25621	YES	YES
16	a	74.74	0.12606	YES	YES
17	a	81.18	1.49195	YES	YES
18	a	82.20	0.16761	YES	YES
19	a	224.60	0.01051	YES	YES
20	a	251.70	6.37219	YES	YES
21	a	347.84	2.64514	YES	YES
22	a	361.93	0.00324	YES	YES
23	a	362.78	0.05929	YES	YES
24	a	389.11	3.63337	YES	YES

25	a	396.20	0.08999	YES	YES
26	a	406.27	0.02552	YES	YES
27	a	423.05	22.32343	YES	YES
28	a	444.67	5.41341	YES	YES
29	a	458.83	3.39187	YES	YES
30	a	471.85	5.05380	YES	YES
31	a	483.24	8.31897	YES	YES
32	a	493.92	0.46120	YES	YES
33	a	496.33	1.69613	YES	YES
34	a	503.04	0.98293	YES	YES
35	a	504.06	1.27494	YES	YES
36	a	508.88	1.81728	YES	YES
37	a	529.70	89.82660	YES	YES
38	a	546.12	2.20435	YES	YES
39	a	614.73	3.30528	YES	YES
40	a	658.72	183.14343	YES	YES
41	a	659.80	100.74203	YES	YES
42	a	670.39	114.79600	YES	YES
43	a	674.35	28.64590	YES	YES
44	a	727.70	45.26555	YES	YES
45	a	807.02	53.48110	YES	YES
46	a	809.46	0.04780	YES	YES
47	a	868.00	5.61768	YES	YES
48	a	924.23	0.12107	YES	YES
49	a	937.95	0.11919	YES	YES
50	a	987.94	16.57142	YES	YES
51	a	1017.46	6.56160	YES	YES
52	a	1067.02	9.99415	YES	YES
53	a	1139.54	1.15874	YES	YES
54	a	1155.30	17.90912	YES	YES
55	a	1232.61	27.49235	YES	YES
56	a	1290.15	4.44501	YES	YES
57	a	1336.84	3.21160	YES	YES
58	a	1438.86	0.11172	YES	YES
59	a	1475.20	79.85637	YES	YES
60	a	1554.61	9.62907	YES	YES
61	a	1587.00	431.42609	YES	YES
62	a	1852.48	3792.14958	YES	YES
63	a	1877.01	2178.37894	YES	YES
64	a	1887.05	2183.74623	YES	YES
65	a	1898.17	524.98490	YES	YES
66	a	1903.43	150.04270	YES	YES
67	a	1999.12	86.56611	YES	YES
68	a	3082.80	8.20934	YES	YES
69	a	3091.06	21.61089	YES	YES
70	a	3109.67	42.28924	YES	YES
71	a	3119.16	20.49379	YES	YES
72	a	3126.73	4.03312	YES	YES

[V(CO)$_5$(*p*-CNC$_6$H$_4$F)]$^-$

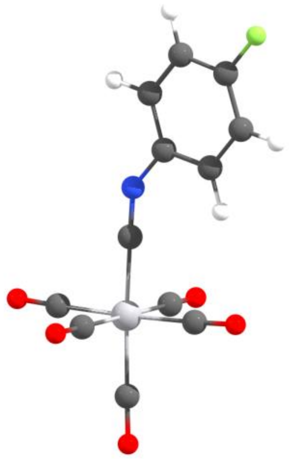

Method: (RI-)BP86(D3BJ)/def2-TZVPP
Symmetry: c1

Cartesian coordinates in Ångström:

V	2.0406964	-0.9655974	-1.4359087
F	-5.2767947	-0.0219862	2.2245075
H	-3.3599736	0.9237067	3.7214548
H	-0.9211194	0.8939767	3.1065164
H	-1.8864734	-1.0389292	-0.6155511
H	-4.3184928	-1.0054121	0.0024580
O	3.7632281	-1.9171068	-3.8726537
O	1.5231181	1.7615025	-2.8699790
O	-0.5168244	-2.1595612	-2.7637568
O	2.5776720	-3.6807214	0.0153594
O	4.4933577	0.2937786	0.0415432
N	0.0902467	-0.0797308	0.8848844
C	0.4553570	-1.7202823	-2.2826586
C	3.5883380	-0.1806128	-0.5208076
C	1.7196986	0.7423873	-2.3357981
C	2.3791336	-2.6666253	-0.5272575
C	3.1301825	-1.5592173	-2.9595605
C	0.8545053	-0.4200532	-0.0028694
C	-1.2463143	-0.0717577	1.2090491
C	-2.2143802	-0.6106144	0.3304433
C	-1.6707393	0.4804529	2.4332556
C	-3.0218495	0.4986949	2.7772381
C	-3.5642195	-0.5933231	0.6718620
C	-3.9478230	-0.0388083	1.8896792

SCF energy GEOOPT = -1935.273908510 H
ZPE = 336.8 kJ/mol
FREEH energy = 391.18 kJ/mol
FREEH entropy = 0.67865 kJ/mol/K

mode #	symmetry	wave number cm**(-1)	IR intensity km/mol	selection rules IR	RAMAN
7	a	11.39	0.00853	YES	YES
8	a	24.40	0.27378	YES	YES
9	a	26.18	0.03039	YES	YES
10	a	39.55	0.19114	YES	YES
11	a	46.42	1.64361	YES	YES
12	a	46.97	0.15784	YES	YES
13	a	61.97	2.23655	YES	YES
14	a	70.14	0.23899	YES	YES
15	a	74.12	0.30758	YES	YES
16	a	74.67	0.15429	YES	YES
17	a	75.31	0.01988	YES	YES
18	a	79.16	0.80065	YES	YES
19	a	163.01	0.61642	YES	YES
20	a	233.34	5.32969	YES	YES
21	a	335.97	1.36286	YES	YES
22	a	338.36	0.04850	YES	YES
23	a	361.95	0.00020	YES	YES
24	a	373.88	0.00646	YES	YES
25	a	382.00	16.33102	YES	YES

26	a	389.44	1.04973	YES	YES
27	a	396.38	0.11166	YES	YES
28	a	417.45	0.08143	YES	YES
29	a	431.01	12.60824	YES	YES
30	a	437.22	8.56996	YES	YES
31	a	459.87	3.87242	YES	YES
32	a	468.84	29.32715	YES	YES
33	a	486.91	9.95198	YES	YES
34	a	492.02	45.12929	YES	YES
35	a	493.59	16.77854	YES	YES
36	a	495.72	4.67548	YES	YES
37	a	503.32	1.41517	YES	YES
38	a	506.46	10.50192	YES	YES
39	a	508.74	2.08305	YES	YES
40	a	539.74	35.30767	YES	YES
41	a	632.56	16.04866	YES	YES
42	a	655.52	164.55200	YES	YES
43	a	659.93	108.33924	YES	YES
44	a	670.83	105.74922	YES	YES
45	a	687.40	0.48344	YES	YES
46	a	738.90	20.38138	YES	YES
47	a	779.09	1.71823	YES	YES
48	a	813.36	51.85637	YES	YES
49	a	835.73	102.91600	YES	YES
50	a	902.62	0.03588	YES	YES
51	a	926.20	0.00549	YES	YES
52	a	996.64	0.05179	YES	YES
53	a	1078.83	14.14427	YES	YES
54	a	1131.92	5.37496	YES	YES
55	a	1189.63	216.80285	YES	YES
56	a	1235.57	1.24186	YES	YES
57	a	1269.08	0.30803	YES	YES
58	a	1318.94	3.46049	YES	YES
59	a	1405.08	2.06129	YES	YES
60	a	1483.28	349.75226	YES	YES
61	a	1566.16	6.35217	YES	YES
62	a	1591.07	92.14056	YES	YES
63	a	1843.64	3666.66439	YES	YES
64	a	1876.28	2097.02471	YES	YES
65	a	1887.65	2179.59704	YES	YES
66	a	1899.68	570.34351	YES	YES
67	a	1904.69	241.05115	YES	YES
68	a	1998.78	109.03627	YES	YES
69	a	3113.50	14.68596	YES	YES
70	a	3117.03	5.34305	YES	YES
71	a	3128.98	12.21299	YES	YES
72	a	3133.07	4.05769	YES	YES

$[V(CO)_5(CNC_6F_5)]^-$

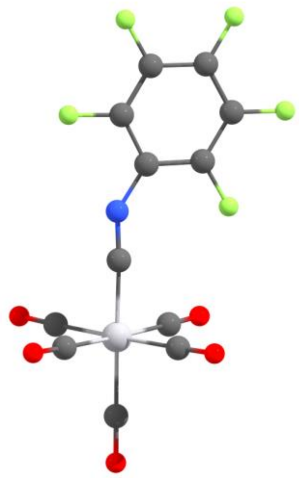

Method: (RI-)BP86(D3BJ)/def2-TZVPP
Symmetry: c1

Cartesian coordinates in Ångström:

V	0.6303031	-0.1680881	0.5927223
F	-6.0084498	1.8491786	5.3003544
F	-4.3738084	4.0435723	5.0695085
F	-2.0773436	3.8983215	3.6248796
F	-3.0176799	-0.6579605	2.6129542
F	-5.3134013	-0.4984147	4.0639597
O	2.5670053	-1.8762115	-1.1906207
O	1.2701960	2.4187115	-1.0383434
O	-1.7514381	-0.8150154	-1.3241469
O	-0.0715739	-2.6802461	2.3124602
O	2.9427783	0.5399585	2.5721919
N	-1.3134495	1.5670939	2.3568405
C	-0.8550699	-0.5800856	-0.6163616
C	2.0892640	0.2680444	1.8259402
C	1.0408234	1.4446378	-0.4401734
C	0.1965790	-1.7499296	1.6630254
C	1.8499326	-1.2406749	-0.5272988
C	-0.5841419	0.8529326	1.6761910
C	-2.4639087	1.6134180	3.0680819
C	-3.3329236	0.5023466	3.2084589
C	-2.8580908	2.8053760	3.7198292
C	-4.0330972	2.8868878	4.4610986
C	-4.5088858	0.5791778	3.9492327
C	-4.8673793	1.7729996	4.5812062

SCF energy GEOOPT = -2332.394912016 H
ZPE = 252.4 kJ/mol
FREEH energy = 317.24 kJ/mol
FREEH entropy = 0.76685 kJ/mol/K

mode	symmetry	wave number	IR intensity	selection rules	
#		cm**(-1)	km/mol	IR	RAMAN
7	a	4.48	0.03282	YES	YES
8	a	15.85	0.00536	YES	YES
9	a	18.94	0.14959	YES	YES
10	a	39.61	0.66009	YES	YES
11	a	40.47	0.19815	YES	YES
12	a	41.99	0.32541	YES	YES
13	a	60.18	1.62669	YES	YES
14	a	63.61	0.05392	YES	YES
15	a	68.81	0.14200	YES	YES
16	a	73.07	0.26532	YES	YES
17	a	74.06	0.14953	YES	YES
18	a	75.34	0.29667	YES	YES
19	a	127.69	0.01844	YES	YES
20	a	130.65	0.00070	YES	YES
21	a	176.34	0.01602	YES	YES
22	a	182.03	1.51972	YES	YES
23	a	202.90	1.50875	YES	YES
24	a	265.24	0.47249	YES	YES

25	a	268.24	1.19186	YES	YES
26	a	293.02	0.67853	YES	YES
27	a	303.81	5.18608	YES	YES
28	a	314.84	0.17043	YES	YES
29	a	337.12	2.49019	YES	YES
30	a	344.94	0.01021	YES	YES
31	a	361.06	0.00111	YES	YES
32	a	362.18	0.04177	YES	YES
33	a	384.65	16.79719	YES	YES
34	a	389.78	0.82390	YES	YES
35	a	395.24	0.43105	YES	YES
36	a	425.55	3.24464	YES	YES
37	a	432.60	1.94247	YES	YES
38	a	457.98	6.88093	YES	YES
39	a	458.79	19.44425	YES	YES
40	a	471.61	4.75001	YES	YES
41	a	490.04	0.30273	YES	YES
42	a	494.35	0.24492	YES	YES
43	a	494.88	0.10017	YES	YES
44	a	501.19	0.27248	YES	YES
45	a	504.60	0.30854	YES	YES
46	a	523.08	17.24554	YES	YES
47	a	551.72	79.64812	YES	YES
48	a	593.86	4.81110	YES	YES
49	a	597.87	0.09718	YES	YES
50	a	612.52	0.03920	YES	YES
51	a	652.99	13.07634	YES	YES
52	a	659.03	96.13602	YES	YES
53	a	660.39	266.19382	YES	YES
54	a	668.39	150.82898	YES	YES
55	a	765.37	0.66205	YES	YES
56	a	970.31	214.02024	YES	YES
57	a	977.74	124.71161	YES	YES
58	a	1109.60	1.98144	YES	YES
59	a	1127.69	21.96952	YES	YES
60	a	1278.43	5.29740	YES	YES
61	a	1307.23	12.10609	YES	YES
62	a	1418.57	83.60857	YES	YES
63	a	1468.56	515.82274	YES	YES
64	a	1474.21	348.35759	YES	YES
65	a	1568.80	21.94069	YES	YES
66	a	1605.54	264.65280	YES	YES
67	a	1834.82	3885.01890	YES	YES
68	a	1895.66	2101.92311	YES	YES
69	a	1896.62	2087.09573	YES	YES
70	a	1910.23	607.77146	YES	YES
71	a	1912.30	13.66253	YES	YES
72	a	2004.19	205.40772	YES	YES

$Cr(CO)_5$

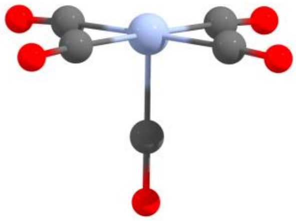

```
Method: (RI-)BP86(D3BJ)/def2-TZVPP
Symmetry: c1

Cartesian coordinates in Ångström:
Cr    0.0000221    0.0000000    0.4676910
O     2.1591507   -2.1592196    0.3839785
O     2.1591507    2.1592196    0.3839786
O     0.0000129   -0.0000000   -2.5152849
O    -2.1591914   -2.1591224    0.3839950
O    -2.1591914    2.1591224    0.3839950
C     0.0000168   -0.0000000   -1.3545061
C     1.3445576    1.3445709    0.4544448
C    -1.3445428   -1.3445295    0.4544531
C     1.3445576   -1.3445709    0.4544448
C    -1.3445428    1.3445295    0.4544531

SCF energy GEOOPT =  -1611.723045199 H
ZPE =   107.6 kJ/mol
FREEH energy =   138.64 kJ/mol
FREEH entropy =   0.46304 kJ/mol/K
mode      symmetry     wave number   IR intensity    selection rules
#                       cm**(-1)         km/mol         IR      RAMAN
     7        a             50.37          0.00000       YES      YES
     8        a             71.09          0.37528       YES      YES
     9        a             71.09          0.37527       YES      YES
    10        a             85.83          0.00000       YES      YES
    11        a             94.55          1.25475       YES      YES
    12        a             95.66          1.99013       YES      YES
    13        a             95.66          1.99012       YES      YES
    14        a            361.73          0.01533       YES      YES
    15        a            361.73          0.01533       YES      YES
    16        a            364.66          0.00000       YES      YES
    17        a            405.51          1.83141       YES      YES
    18        a            409.65          0.00000       YES      YES
    19        a            451.38         31.42649       YES      YES
    20        a            451.38         31.42649       YES      YES
    21        a            494.49          0.21503       YES      YES
    22        a            506.05          0.00000       YES      YES
    23        a            535.05          0.00000       YES      YES
    24        a            556.30          7.27500       YES      YES
    25        a            556.30          7.27499       YES      YES
    26        a            658.84         67.32356       YES      YES
    27        a            658.84         67.32354       YES      YES
    28        a            681.91         68.11413       YES      YES
    29        a           1956.35        730.75895       YES      YES
    30        a           1970.94       1798.34749       YES      YES
    31        a           1970.94       1798.34706       YES      YES
    32        a           1997.24          0.00002       YES      YES
    33        a           2078.84          6.42568       YES      YES
```

$Cr(CO)_5(N_2)$

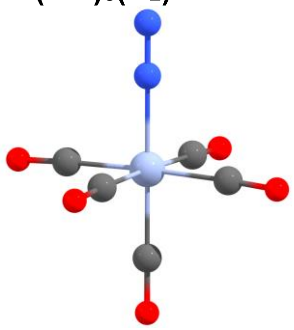

Method: (RI-)BP86(D3BJ)/def2-TZVPP
Symmetry: c1

Cartesian coordinates in Ångström:

Cr	-0.0000104	-0.0000000	0.0346478
O	2.1604208	-2.1603900	-0.0741576
O	2.1604208	2.1603900	-0.0741576
O	-0.0000059	0.0000000	-2.9919141
O	-2.1603991	-2.1604298	-0.0741548
N	-0.0000021	-0.0000000	3.1178490
O	-2.1603991	2.1604298	-0.0741548
C	-0.0000098	0.0000000	-1.8362698
N	-0.0000081	-0.0000000	2.0029244
C	1.3468554	1.3468502	-0.0079604
C	-1.3468590	-1.3468642	-0.0079591
C	1.3468554	-1.3468502	-0.0079604
C	-1.3468590	1.3468642	-0.0079591

SCF energy GEOOPT = -1721.346736122 H
ZPE = 130.1 kJ/mol
FREEH energy = 167.36 kJ/mol
FREEH entropy = 0.50959 kJ/mol/K

mode	symmetry	wave number	IR intensity	selection rules	
#		cm**(-1)	km/mol	IR	RAMAN
7	a	59.18	0.00000	YES	YES
8	a	62.99	0.07273	YES	YES
9	a	62.99	0.07273	YES	YES
10	a	86.49	0.00000	YES	YES
11	a	89.29	0.13466	YES	YES
12	a	89.29	0.13466	YES	YES
13	a	94.67	1.46575	YES	YES
14	a	101.18	1.05111	YES	YES
15	a	101.18	1.05111	YES	YES
16	a	348.41	0.03617	YES	YES
17	a	348.41	0.03617	YES	YES
18	a	356.26	10.00988	YES	YES
19	a	364.02	0.00000	YES	YES
20	a	403.67	0.18897	YES	YES
21	a	407.54	0.00000	YES	YES
22	a	449.90	14.45855	YES	YES
23	a	449.90	14.45851	YES	YES
24	a	466.78	11.41164	YES	YES
25	a	478.93	7.99176	YES	YES
26	a	478.93	7.99172	YES	YES
27	a	520.96	0.00000	YES	YES
28	a	532.36	0.00000	YES	YES
29	a	542.24	2.55224	YES	YES
30	a	542.24	2.55224	YES	YES
31	a	678.86	134.32483	YES	YES
32	a	678.86	134.32479	YES	YES
33	a	685.74	142.10525	YES	YES
34	a	1986.42	763.49257	YES	YES
35	a	1987.62	1663.26343	YES	YES
36	a	1987.62	1663.26352	YES	YES
37	a	2010.90	0.00000	YES	YES
38	a	2076.56	240.76272	YES	YES
39	a	2215.90	262.32882	YES	YES

$Cr(CO)_6$

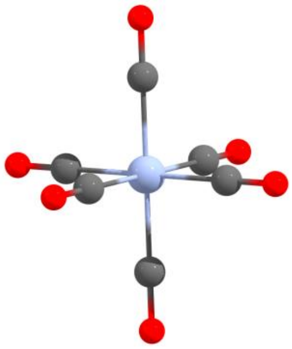

Method: (RI-)BP86(D3BJ)/def2-TZVPP
Symmetry: c1

Cartesian coordinates in Ångström:

Cr	0.0000000	-0.0000000	0.0000000
O	-0.0000000	3.0544183	-0.0000000
O	-0.0000000	-0.0000000	3.0544183
O	-3.0544183	0.0000000	-0.0000000
O	-0.0000000	0.0000000	-3.0544183
O	3.0544183	0.0000000	0.0000000
O	-0.0000000	-3.0544183	-0.0000000
C	-1.9023515	0.0000000	0.0000000
C	1.9023515	-0.0000000	0.0000000
C	0.0000000	-0.0000000	1.9023515
C	-0.0000000	0.0000000	-1.9023515
C	-0.0000000	1.9023515	-0.0000000
C	-0.0000000	-1.9023515	-0.0000000

SCF energy GEOOPT = -1725.164506650 H
ZPE = 130.5 kJ/mol
FREEH energy = 167.35 kJ/mol
FREEH entropy = 0.50910 kJ/mol/K

mode	symmetry	wave number	IR intensity	selection rules	
#		cm**(-1)	km/mol	IR	RAMAN
7	a	58.22	0.00000	YES	YES
8	a	58.22	0.00000	YES	YES
9	a	58.22	0.00000	YES	YES
10	a	87.48	0.00000	YES	YES
11	a	87.48	0.00000	YES	YES
12	a	87.48	0.00000	YES	YES
13	a	95.86	1.20984	YES	YES
14	a	95.86	1.20983	YES	YES
15	a	95.86	1.20982	YES	YES
16	a	368.04	0.00000	YES	YES
17	a	368.04	0.00000	YES	YES
18	a	368.04	0.00000	YES	YES
19	a	405.85	0.00000	YES	YES
20	a	410.88	0.00000	YES	YES
21	a	410.88	0.00000	YES	YES
22	a	462.75	15.65461	YES	YES
23	a	462.75	15.65459	YES	YES
24	a	462.75	15.65429	YES	YES
25	a	520.63	0.00000	YES	YES
26	a	520.63	0.00000	YES	YES
27	a	520.63	0.00000	YES	YES
28	a	535.91	0.00000	YES	YES
29	a	535.91	0.00000	YES	YES
30	a	535.91	0.00000	YES	YES
31	a	694.29	163.90334	YES	YES
32	a	694.29	163.90323	YES	YES
33	a	694.29	163.90461	YES	YES
34	a	1993.74	1625.13851	YES	YES
35	a	1993.74	1625.13843	YES	YES
36	a	1993.74	1625.13873	YES	YES
37	a	2014.61	0.00000	YES	YES
38	a	2014.61	0.00000	YES	YES
39	a	2104.41	0.00000	YES	YES

Cr(CO)$_5$(CNH)

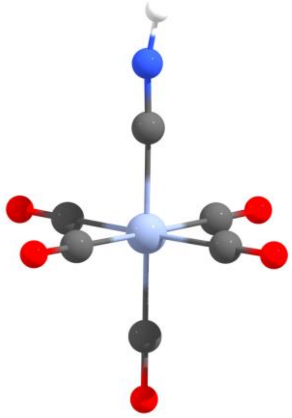

Method: (RI-)BP86(D3BJ)/def2-TZVPP
Symmetry: c1

Cartesian coordinates in Ångström:

Cr	0.3115723	0.0127399	-0.0093471
O	0.3413469	0.0137121	3.0423334
O	0.3474778	-3.0388982	-0.0114267
N	-2.8031425	-0.0199330	0.0302308
O	0.2283180	3.0637406	-0.0071205
O	3.3568922	0.0691977	-0.0619216
O	0.2322388	0.0122445	-3.0603432
C	-1.6242761	-0.0262106	0.0258831
C	2.2021240	0.0478302	-0.0420627
C	0.3354081	-1.8845883	-0.0106554
C	0.2618730	1.9101706	-0.0081745
C	0.3316746	0.0133885	1.8880509
C	0.2641622	0.0126731	-1.9066458
H	-3.7856693	-0.1860673	0.1311993

SCF energy GEOOPT = -1705.247383306 H
ZPE = 153.0 kJ/mol
FREEH energy = 194.21 kJ/mol
FREEH entropy = 0.54893 kJ/mol/K

mode #	symmetry	wave number cm**(-1)	IR intensity km/mol	selection rules IR	selection rules RAMAN
7	a	28.46	85.20366	YES	YES
8	a	53.78	0.94940	YES	YES
9	a	58.52	0.99683	YES	YES
10	a	59.04	5.39471	YES	YES
11	a	82.76	3.33811	YES	YES
12	a	86.25	6.44144	YES	YES
13	a	87.29	7.36124	YES	YES
14	a	91.68	7.04991	YES	YES
15	a	94.65	0.10200	YES	YES
16	a	95.06	1.12310	YES	YES
17	a	123.04	149.54257	YES	YES
18	a	358.18	4.29437	YES	YES
19	a	358.37	3.81984	YES	YES
20	a	369.70	0.00016	YES	YES
21	a	404.19	2.01064	YES	YES
22	a	411.10	0.47002	YES	YES
23	a	414.86	0.02021	YES	YES
24	a	461.83	20.28159	YES	YES
25	a	461.85	20.24804	YES	YES
26	a	465.52	14.86412	YES	YES
27	a	501.04	0.15026	YES	YES
28	a	501.74	0.19082	YES	YES
29	a	524.03	0.02068	YES	YES
30	a	536.90	0.14034	YES	YES
31	a	537.26	0.11644	YES	YES
32	a	538.13	0.02281	YES	YES
33	a	687.57	145.70281	YES	YES
34	a	687.76	146.49132	YES	YES
35	a	697.34	167.31966	YES	YES
36	a	1978.06	1704.80912	YES	YES
37	a	1978.18	1701.83612	YES	YES

38	a	1984.49	1230.94900	YES	YES
39	a	2000.85	2.25849	YES	YES
40	a	2012.12	454.92659	YES	YES
41	a	2095.39	3.14638	YES	YES
42	a	3753.87	673.65132	YES	YES

Cr(CO)$_5$(CNCH$_3$)

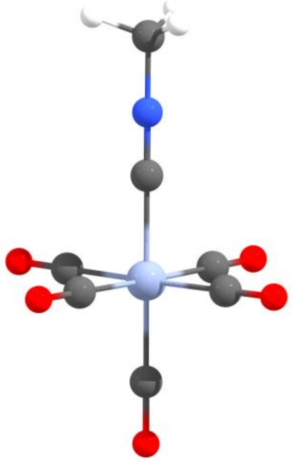

Method: (RI-)BP86(D3BJ)/def2-TZVPP
Symmetry: c1

Cartesian coordinates in Ångström:

Cr	0.8306583	-0.4165844	-0.6861651
H	-2.1024124	1.2081259	3.0580754
H	-3.3465267	0.6076131	1.9242352
H	-2.6772943	2.2529359	1.7269752
O	3.0288371	-1.4966660	-2.4891742
O	1.3272718	2.4077433	-1.7284134
O	-1.2341855	-0.8674485	-2.8869645
O	0.2918871	-3.2218078	0.3866711
O	2.8579840	0.0486271	1.5461709
N	-1.4255046	0.7069448	1.1725519
C	-0.4520551	-0.6967311	-2.0541614
C	2.0910779	-0.1275424	0.7004090
C	1.1401320	1.3379600	-1.3346598
C	0.4967256	-2.1599942	-0.0195612
C	2.1925501	-1.0863459	-1.8036130
C	-0.5795844	0.2832238	0.4743991
C	-2.4395606	1.2199464	2.0132249

SCF energy GEOOPT = -1744.581713014 H
ZPE = 230.1 kJ/mol
FREEH energy = 273.92 kJ/mol
FREEH entropy = 0.58026 kJ/mol/K

mode	symmetry	wave number	IR intensity	selection rules	
#		cm**(-1)	km/mol	IR	RAMAN
7	a	15.17	0.00015	YES	YES
8	a	42.80	2.07600	YES	YES
9	a	43.12	2.08074	YES	YES
10	a	51.14	0.00074	YES	YES
11	a	70.01	0.12878	YES	YES
12	a	70.21	0.13088	YES	YES
13	a	86.62	0.00078	YES	YES
14	a	88.36	1.76065	YES	YES
15	a	90.63	0.20413	YES	YES
16	a	90.96	0.19861	YES	YES
17	a	141.38	2.94264	YES	YES
18	a	141.77	2.94714	YES	YES
19	a	331.17	11.84529	YES	YES
20	a	360.15	0.00219	YES	YES
21	a	360.48	0.00028	YES	YES
22	a	369.64	0.00000	YES	YES
23	a	411.07	0.18399	YES	YES
24	a	416.13	0.00030	YES	YES
25	a	462.03	10.43816	YES	YES
26	a	462.10	10.51124	YES	YES
27	a	462.59	14.89177	YES	YES
28	a	494.82	5.49735	YES	YES
29	a	494.98	5.38891	YES	YES
30	a	525.17	0.00108	YES	YES
31	a	538.53	0.00046	YES	YES

32	a	540.12	0.34561	YES	YES
33	a	540.36	0.36328	YES	YES
34	a	685.78	153.60147	YES	YES
35	a	686.02	153.69786	YES	YES
36	a	695.38	178.26118	YES	YES
37	a	996.57	10.95790	YES	YES
38	a	1104.08	0.13780	YES	YES
39	a	1104.28	0.13322	YES	YES
40	a	1405.47	58.16158	YES	YES
41	a	1439.14	9.21047	YES	YES
42	a	1439.17	9.23166	YES	YES
43	a	1970.97	1715.71689	YES	YES
44	a	1970.99	1716.73529	YES	YES
45	a	1977.58	893.01877	YES	YES
46	a	1994.59	0.02629	YES	YES
47	a	2060.36	448.20288	YES	YES
48	a	2187.14	643.64040	YES	YES
49	a	2969.63	36.66788	YES	YES
50	a	3041.46	3.71474	YES	YES
51	a	3041.66	3.71803	YES	YES

$Cr(CO)_5(CNCF_3)$

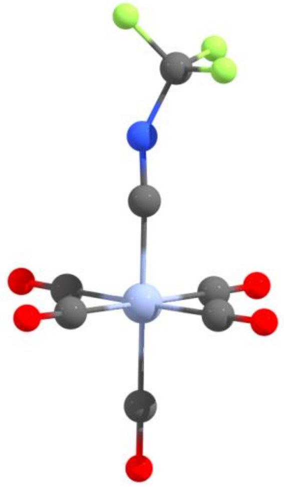

Method: (RI-)BP86(D3BJ)/def2-TZVPP
Symmetry: c1

Cartesian coordinates in Ångström:

Cr	0.8537167	-0.5338541	-0.6495758
F	-2.3609479	-1.2796403	2.8503700
F	-3.3370881	0.6315682	2.4305005
F	-1.4239315	0.5933033	3.4885936
O	3.1299582	-1.1291092	-2.6057323
O	0.8583840	2.4044059	-1.4835309
O	-1.2101471	-1.1611700	-2.8112764
O	0.8090768	-3.4618027	0.2205750
O	2.8795646	0.1040506	1.5457328
N	-1.4754686	0.0947316	1.2782172
C	-0.4260315	-0.9249400	-2.0010297
C	2.1181732	-0.1381019	0.7155468
C	0.8617269	1.2945320	-1.1748511
C	0.8295456	-2.3581489	-0.1096745
C	2.2734781	-0.9052633	-1.8685480
C	-0.5538719	-0.1702251	0.5614591
C	-2.1451074	0.0100239	2.5045836

SCF energy GEOOPT = -2042.454090231 H
ZPE = 169.8 kJ/mol
FREEH energy = 217.93 kJ/mol
FREEH entropy = 0.63770 kJ/mol/K

mode	symmetry	wave number	IR intensity	selection rules	
#		cm**(-1)	km/mol	IR	RAMAN
7	a	7.81	0.00930	YES	YES
8	a	10.82	0.01540	YES	YES
9	a	25.72	0.46743	YES	YES
10	a	52.86	0.01685	YES	YES
11	a	56.36	0.01143	YES	YES
12	a	58.44	1.55010	YES	YES
13	a	78.35	1.76701	YES	YES
14	a	78.98	0.06133	YES	YES
15	a	87.21	0.00976	YES	YES
16	a	89.84	0.47082	YES	YES
17	a	92.84	0.78300	YES	YES
18	a	97.10	0.66737	YES	YES
19	a	264.08	2.53624	YES	YES
20	a	347.68	0.19327	YES	YES
21	a	352.45	0.81327	YES	YES
22	a	367.51	0.00008	YES	YES
23	a	403.52	0.07908	YES	YES
24	a	406.43	4.16964	YES	YES
25	a	411.27	0.01959	YES	YES
26	a	419.76	9.94780	YES	YES
27	a	443.87	17.28380	YES	YES
28	a	459.18	13.99339	YES	YES
29	a	463.87	15.07985	YES	YES
30	a	506.10	0.85849	YES	YES

31	a	513.24	0.69927	YES	YES
32	a	519.34	0.01545	YES	YES
33	a	529.28	0.48127	YES	YES
34	a	529.57	0.03240	YES	YES
35	a	534.81	0.17472	YES	YES
36	a	576.62	0.60904	YES	YES
37	a	587.97	65.27220	YES	YES
38	a	613.30	11.56183	YES	YES
39	a	686.08	160.78693	YES	YES
40	a	686.56	208.51039	YES	YES
41	a	693.71	214.39024	YES	YES
42	a	833.29	160.16602	YES	YES
43	a	1109.63	273.25172	YES	YES
44	a	1124.99	300.85965	YES	YES
45	a	1163.80	1043.07810	YES	YES
46	a	1987.75	2916.69538	YES	YES
47	a	1993.19	1583.65103	YES	YES
48	a	1993.23	1594.28413	YES	YES
49	a	2010.48	9.18500	YES	YES
50	a	2013.98	1.73331	YES	YES
51	a	2107.44	31.45134	YES	YES

$Cr(CO)_5(CNC_6H_5)$

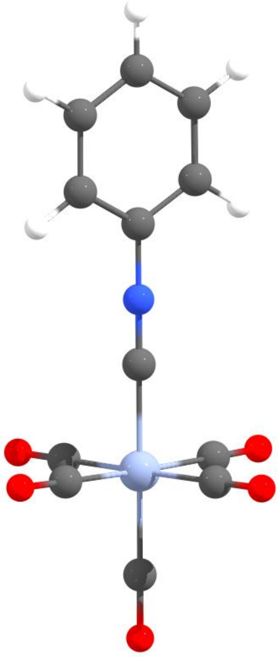

Method: (RI-)BP86(D3BJ)/def2-TZVPP
Symmetry: c1

Cartesian coordinates in Ångström:

Cr	2.1324017	-0.9296916	-1.6807281
H	-4.6776726	-0.1072918	3.1227869
H	-3.5824005	1.9121146	2.1623682
H	-1.5550604	1.6690624	0.7288214
H	-1.7184125	-2.6071286	1.2173427
H	-3.7464564	-2.3677953	2.6504706
O	4.6107322	-1.2424972	-3.4213914
O	1.8214998	1.9776036	-2.5525314
O	0.3746327	-1.8235999	-4.0107442
O	2.3572492	-3.8251358	-0.7459239
O	3.8695742	-0.0326379	0.6634268
N	-0.4093675	-0.6151969	0.1060898
C	1.0389234	-1.4849948	-3.1294867
C	3.2131875	-0.3724158	-0.2232882
C	1.9430940	0.8764967	-2.2263816
C	2.2774493	-2.7297640	-1.1029012
C	3.6701705	-1.1233973	-2.7601396
C	0.5516829	-0.7331867	-0.5707357
C	-1.5273125	-0.4820168	0.8958625
C	-2.1383405	-1.6264055	1.4333664
C	-2.0457668	0.7966580	1.1565285
C	-3.1779825	0.9214392	1.9579539
C	-3.2699925	-1.4818930	2.2322142
C	-3.7924426	-0.2125960	2.4969399

SCF energy GEOOPT = -1936.417585396 H
ZPE =   365.9 kJ/mol
FREEH energy =   416.93 kJ/mol
FREEH entropy =   0.65392 kJ/mol/K

mode	symmetry	wave number	IR intensity	selection rules	
#		cm**(-1)	km/mol	IR	RAMAN
7	a	4.73	0.00015	YES	YES
8	a	20.21	0.01880	YES	YES
9	a	24.97	0.07038	YES	YES
10	a	51.55	0.00773	YES	YES
11	a	59.25	0.10731	YES	YES
12	a	64.15	0.05271	YES	YES
13	a	80.61	0.21379	YES	YES
14	a	80.92	1.54044	YES	YES

15	a	85.89	0.09806	YES	YES
16	a	86.91	0.00087	YES	YES
17	a	95.12	0.55037	YES	YES
18	a	98.50	0.64645	YES	YES
19	a	228.83	5.24301	YES	YES
20	a	249.30	0.00065	YES	YES
21	a	337.31	0.42545	YES	YES
22	a	369.58	0.00002	YES	YES
23	a	370.44	0.00711	YES	YES
24	a	399.40	0.00003	YES	YES
25	a	410.07	1.73840	YES	YES
26	a	415.33	0.00012	YES	YES
27	a	434.32	2.15402	YES	YES
28	a	456.08	15.10771	YES	YES
29	a	460.99	8.12118	YES	YES
30	a	468.10	10.20540	YES	YES
31	a	488.47	14.67060	YES	YES
32	a	503.71	2.45267	YES	YES
33	a	508.60	1.55371	YES	YES
34	a	523.91	0.33418	YES	YES
35	a	537.53	0.17416	YES	YES
36	a	537.72	0.34660	YES	YES
37	a	538.46	0.04339	YES	YES
38	a	539.89	48.92625	YES	YES
39	a	615.74	0.03683	YES	YES
40	a	680.56	0.26357	YES	YES
41	a	685.18	171.82456	YES	YES
42	a	686.01	147.19895	YES	YES
43	a	691.50	262.63512	YES	YES
44	a	747.21	48.95391	YES	YES
45	a	801.59	31.59717	YES	YES
46	a	817.11	0.00003	YES	YES
47	a	899.02	3.33651	YES	YES
48	a	950.35	0.00014	YES	YES
49	a	971.93	0.03425	YES	YES
50	a	994.21	0.51692	YES	YES
51	a	1022.34	2.06304	YES	YES
52	a	1075.13	7.55278	YES	YES
53	a	1153.74	0.00666	YES	YES
54	a	1160.33	0.12494	YES	YES
55	a	1215.20	1.14014	YES	YES
56	a	1299.21	0.00065	YES	YES
57	a	1338.07	0.36545	YES	YES
58	a	1444.44	3.82224	YES	YES
59	a	1477.41	36.93334	YES	YES
60	a	1573.44	3.90043	YES	YES
61	a	1590.35	47.29696	YES	YES
62	a	1973.46	1612.05046	YES	YES
63	a	1976.73	1654.30605	YES	YES
64	a	1981.57	1114.00311	YES	YES
65	a	1998.29	0.23257	YES	YES
66	a	2050.97	1214.29342	YES	YES
67	a	2146.47	681.47858	YES	YES
68	a	3108.83	0.91789	YES	YES
69	a	3117.38	4.74464	YES	YES
70	a	3127.62	11.59608	YES	YES
71	a	3135.58	4.38499	YES	YES
72	a	3138.17	2.56006	YES	YES

$Cr(CO)_5(p\text{-}CNC_6H_4F)$

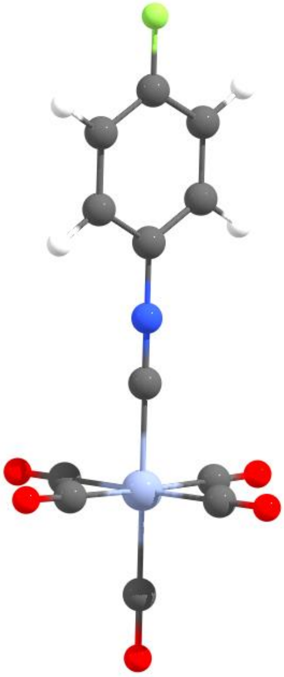

```
Method: (RI-)BP86(D3BJ)/def2-TZVPP
Symmetry: c1

Cartesian coordinates in Ångström:
Cr     2.1171713   -1.0091542   -1.5549374
F     -5.0037066    0.4113037    3.0736634
H     -2.6009738    1.0255048    3.8505266
H     -0.5239218    0.6005490    2.5003593
H     -2.9243381   -1.1274982   -0.6332798
H     -4.9961445   -0.6983413    0.7236715
O      4.6482493   -1.4570877   -3.1879923
O      1.6173736    1.6152358   -3.0318591
O      0.4455968   -2.4956366   -3.6299684
O      2.5992435   -3.6307243   -0.0663815
O      3.6904920    0.4886813    0.5868557
N     -0.4740356   -0.5196880    0.1175511
C      1.0834828   -1.9340062   -2.8481812
C      3.1017605   -0.0788930   -0.2285722
C      1.8073773    0.6227638   -2.4738748
C      2.4160103   -2.6396839   -0.6290991
C      3.6876764   -1.2877456   -2.5680481
C      0.5057566   -0.7108726   -0.5149573
C     -1.6079806   -0.2878529    0.8579395
C     -2.8629338   -0.6582896    0.3466784
C     -1.5042400    0.3197107    2.1204316
C     -2.6526740    0.5562606    2.8699739
C     -4.0116247   -0.4217946    1.0958995
C     -3.8870868    0.1814189    2.3450506

SCF energy GEOOPT =  -2035.705472587 H
ZPE =    344.5 kJ/mol
FREEH energy =    397.76 kJ/mol
FREEH entropy =    0.67760 kJ/mol/K
mode      symmetry      wave number   IR intensity    selection rules
#                        cm**(-1)        km/mol          IR      RAMAN
     7         a             2.95         0.00081       YES      YES
     8         a            17.24         0.08024       YES      YES
     9         a            22.06         0.03450       YES      YES
    10         a            51.29         0.00813       YES      YES
    11         a            57.28         0.37038       YES      YES
    12         a            59.98         0.47462       YES      YES
    13         a            78.59         0.51704       YES      YES
    14         a            78.78         0.16249       YES      YES
    15         a            79.08         1.21324       YES      YES
```

16	a	86.94	0.00016	YES	YES
17	a	93.52	0.34612	YES	YES
18	a	94.26	0.38757	YES	YES
19	a	182.77	0.81920	YES	YES
20	a	214.85	2.32734	YES	YES
21	a	325.25	1.88578	YES	YES
22	a	339.29	0.00254	YES	YES
23	a	369.42	0.00001	YES	YES
24	a	378.78	1.47027	YES	YES
25	a	396.50	0.01308	YES	YES
26	a	409.84	1.84031	YES	YES
27	a	410.80	0.00021	YES	YES
28	a	415.25	0.00563	YES	YES
29	a	447.62	1.60554	YES	YES
30	a	452.49	2.14942	YES	YES
31	a	462.80	10.69821	YES	YES
32	a	469.43	10.45438	YES	YES
33	a	484.01	91.89693	YES	YES
34	a	496.54	14.05166	YES	YES
35	a	503.38	1.95102	YES	YES
36	a	507.55	7.03498	YES	YES
37	a	523.72	0.34938	YES	YES
38	a	536.98	0.14248	YES	YES
39	a	537.60	0.00939	YES	YES
40	a	537.90	0.02665	YES	YES
41	a	634.56	0.00731	YES	YES
42	a	683.72	147.32606	YES	YES
43	a	685.84	148.78321	YES	YES
44	a	686.72	234.15660	YES	YES
45	a	693.59	1.35063	YES	YES
46	a	739.90	34.53041	YES	YES
47	a	790.11	0.00014	YES	YES
48	a	825.53	60.86167	YES	YES
49	a	842.55	37.73777	YES	YES
50	a	918.55	0.02675	YES	YES
51	a	935.00	0.00019	YES	YES
52	a	1001.54	1.11295	YES	YES
53	a	1088.58	10.48252	YES	YES
54	a	1140.29	23.85592	YES	YES
55	a	1203.97	64.50094	YES	YES
56	a	1237.64	125.41716	YES	YES
57	a	1275.47	1.16354	YES	YES
58	a	1326.51	0.19689	YES	YES
59	a	1411.35	0.26078	YES	YES
60	a	1489.15	213.96438	YES	YES
61	a	1578.21	1.10093	YES	YES
62	a	1596.28	12.12666	YES	YES
63	a	1973.56	1609.93844	YES	YES
64	a	1976.95	1654.15988	YES	YES
65	a	1982.37	1105.51054	YES	YES
66	a	1998.43	0.23060	YES	YES
67	a	2050.96	1209.92801	YES	YES
68	a	2145.48	648.97174	YES	YES
69	a	3130.61	4.27303	YES	YES
70	a	3132.39	0.18376	YES	YES
71	a	3144.04	0.06430	YES	YES
72	a	3144.88	0.60596	YES	YES

$Cr(CO)_5(CNC_6F_5)$

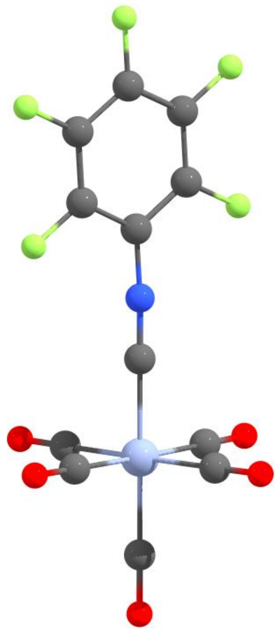

```
Method: (RI-)BP86(D3BJ)/def2-TZVPP
Symmetry: c1

Cartesian coordinates in Ångström:
Cr     0.5665683   -0.2985830    0.5565232
F     -6.6931284   -0.4193145    5.1777030
F     -5.6500045    1.9205272    4.2282998
F     -3.3382073    1.8892935    2.7740781
F     -3.1192737   -2.8259730    3.2291153
F     -5.4306385   -2.7910402    4.6827672
O      3.1065463   -0.1003463   -1.1210863
O     -0.2065865    2.5484560   -0.2265704
O     -0.9643208   -1.3797055   -1.8544385
O      1.2802745   -3.1500844    1.3804718
O      2.0664783    0.7759782    2.9900953
N     -2.0323688   -0.4835015    2.2496212
C     -0.3828590   -0.9714895   -0.9457756
C      1.5023096    0.3707461    2.0692790
C      0.0894240    1.4730891    0.0674115
C      1.0135684   -2.0727307    1.0666536
C      2.1459318   -0.1763947   -0.4857508
C     -1.0384021   -0.4109519    1.6057563
C     -3.1805890   -0.4688867    2.9723349
C     -3.7347156   -1.6633588    3.4717576
C     -3.8470545    0.7431892    3.2398188
C     -5.0266026    0.7624359    3.9807000
C     -4.9142667   -1.6490659    4.2133103
C     -5.5610830   -0.4351687    4.4676849

SCF energy GEOOPT =  -2432.817325144 H
ZPE =    260.5 kJ/mol
FREEH energy =    321.49 kJ/mol
FREEH entropy =    0.71060 kJ/mol/K
mode      symmetry     wave number   IR intensity    selection rules
#                       cm**(-1)        km/mol         IR      RAMAN
     8         a             9.52         0.04274       YES      YES
     9         a            20.22         0.04137       YES      YES
    10         a            45.81         0.06722       YES      YES
    11         a            53.34         0.13048       YES      YES
    12         a            53.42         0.01236       YES      YES
    13         a            73.76         0.10436       YES      YES
    14         a            74.31         0.01089       YES      YES
    15         a            75.89         1.19233       YES      YES
    16         a            86.76         0.00534       YES      YES
    17         a            92.19         0.64929       YES      YES
```

18	a	92.59	0.63200	YES	YES
19	a	128.17	0.00008	YES	YES
20	a	144.00	0.06272	YES	YES
21	a	185.68	0.33079	YES	YES
22	a	188.64	0.18921	YES	YES
23	a	218.82	2.07347	YES	YES
24	a	265.97	0.06093	YES	YES
25	a	268.79	0.55356	YES	YES
26	a	285.93	0.13687	YES	YES
27	a	307.11	1.26164	YES	YES
28	a	330.30	5.45267	YES	YES
29	a	345.87	0.03180	YES	YES
30	a	356.33	0.00028	YES	YES
31	a	368.03	0.00027	YES	YES
32	a	370.26	0.81680	YES	YES
33	a	377.34	0.01254	YES	YES
34	a	408.23	3.04380	YES	YES
35	a	412.94	0.00356	YES	YES
36	a	435.02	0.53633	YES	YES
37	a	444.36	0.75718	YES	YES
38	a	459.80	15.53274	YES	YES
39	a	460.50	20.33329	YES	YES
40	a	462.40	38.38824	YES	YES
41	a	499.86	2.20384	YES	YES
42	a	500.43	2.10785	YES	YES
43	a	521.59	0.39094	YES	YES
44	a	533.02	0.05487	YES	YES
45	a	533.86	0.04522	YES	YES
46	a	535.82	0.10972	YES	YES
47	a	558.05	30.89607	YES	YES
48	a	599.79	25.52129	YES	YES
49	a	625.24	0.05524	YES	YES
50	a	627.09	0.00047	YES	YES
51	a	676.36	5.57997	YES	YES
52	a	682.48	144.79977	YES	YES
53	a	684.91	143.14642	YES	YES
54	a	691.45	354.88652	YES	YES
55	a	768.61	0.01092	YES	YES
56	a	983.86	68.88672	YES	YES
57	a	986.95	195.07298	YES	YES
58	a	1137.46	3.37561	YES	YES
59	a	1142.00	36.52429	YES	YES
60	a	1299.93	5.63190	YES	YES
61	a	1312.21	0.67875	YES	YES
62	a	1433.12	84.85806	YES	YES
63	a	1484.82	351.84312	YES	YES
64	a	1485.84	402.10077	YES	YES
65	a	1586.42	9.03238	YES	YES
66	a	1612.11	4.37072	YES	YES
67	a	1983.03	1610.16391	YES	YES
68	a	1983.42	1567.67322	YES	YES
69	a	1991.38	1201.10645	YES	YES
70	a	2005.40	1.95098	YES	YES
71	a	2048.42	1640.52940	YES	YES
72	a	2138.34	623.19242	YES	YES

$[Mn(CO)_5]^+$

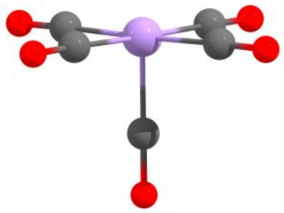

Method: (RI-)BP86(D3BJ)/def2-TZVPP
Symmetry: c4v

Cartesian coordinates in Ångström:

Mn	0.0000000	0.0000000	0.4165787
O	2.1409515	-2.1409515	0.4298023
O	2.1409515	2.1409515	0.4298023
O	0.0000000	0.0000000	-2.5269165
O	-2.1409515	-2.1409515	0.4298023
O	-2.1409515	2.1409515	0.4298023
C	0.0000000	0.0000000	-1.3824419
C	1.3359739	1.3359739	0.4433927
C	-1.3359739	-1.3359739	0.4433927
C	1.3359739	-1.3359739	0.4433927
C	-1.3359739	1.3359739	0.4433927

SCF energy GEOOPT = -1717.995275464 H
ZPE = 109.5 kJ/mol
FREEH energy = 140.76 kJ/mol
FREEH entropy = 0.44797 kJ/mol/K

mode	symmetry	wave number	IR intensity	selection rules	
#		cm**(-1)	km/mol	IR	RAMAN
7	b2	62.95	0.00000	NO	YES
8	e	81.78	0.07739	YES	YES
9	e	81.78	0.07739	YES	YES
10	b1	92.66	0.00000	NO	YES
11	e	103.12	2.32685	YES	YES
12	e	103.12	2.32685	YES	YES
13	a1	105.20	1.56747	YES	YES
14	e	350.96	0.03179	YES	YES
15	e	350.96	0.03179	YES	YES
16	a2	353.55	0.00000	NO	NO
17	b2	389.89	0.00000	NO	YES
18	a1	391.82	2.54294	YES	YES
19	e	412.74	30.22049	YES	YES
20	e	412.74	30.22049	YES	YES
21	a1	475.86	0.89121	YES	YES
22	b2	494.44	0.00000	NO	YES
23	b1	532.28	0.00000	NO	YES
24	e	538.24	5.82371	YES	YES
25	e	538.24	5.82371	YES	YES
26	e	636.49	71.80747	YES	YES
27	e	636.49	71.80747	YES	YES
28	a1	650.93	72.80063	YES	YES
29	a1	2068.04	460.39977	YES	YES
30	e	2085.75	964.44557	YES	YES
31	e	2085.75	964.44557	YES	YES
32	b2	2108.57	0.00000	NO	YES
33	a1	2164.06	0.05121	YES	YES

[Mn(CO)$_5$(N$_2$)]$^+$

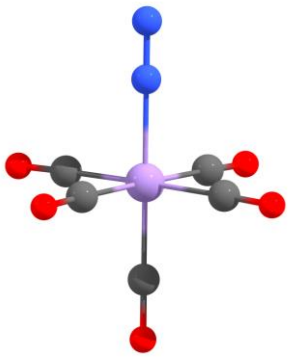

Method: (RI-)BP86(D3BJ)/def2-TZVPP
Symmetry: c4v

Cartesian coordinates in Ångström:

Mn	-0.0000000	0.0000000	0.0074509
O	2.1396295	-2.1396295	-0.0473505
O	2.1396295	2.1396295	-0.0473505
O	0.0000000	0.0000000	-2.9807174
O	-2.1396295	-2.1396295	-0.0473505
N	0.0000000	0.0000000	3.0785857
O	-2.1396295	2.1396295	-0.0473505
C	0.0000000	0.0000000	-1.8393990
N	0.0000000	0.0000000	1.9710167
C	1.3349848	1.3349848	-0.0118837
C	-1.3349848	-1.3349848	-0.0118837
C	1.3349848	-1.3349848	-0.0118837
C	-1.3349848	1.3349848	-0.0118837

SCF energy GEOOPT = -1827.617187358 H
ZPE = 132.3 kJ/mol
FREEH energy = 169.74 kJ/mol
FREEH entropy = 0.49373 kJ/mol/K

mode	symmetry	wave number	IR intensity	selection rules	
#		cm**(-1)	km/mol	IR	RAMAN
7	b2	69.70	0.00000	NO	YES
8	e	72.79	0.05287	YES	YES
9	e	72.79	0.05287	YES	YES
10	b1	93.06	0.00000	NO	YES
11	e	94.78	0.09178	YES	YES
12	e	94.78	0.09178	YES	YES
13	a1	106.52	1.31873	YES	YES
14	e	110.98	1.08881	YES	YES
15	e	110.98	1.08881	YES	YES
16	e	333.18	0.13912	YES	YES
17	e	333.18	0.13912	YES	YES
18	a1	333.51	5.51660	YES	YES
19	a2	355.07	0.00000	NO	NO
20	b2	393.27	0.00000	NO	YES
21	a1	394.20	0.46921	YES	YES
22	e	419.88	21.56553	YES	YES
23	e	419.88	21.56553	YES	YES
24	a1	446.94	9.77787	YES	YES
25	e	464.27	3.92894	YES	YES
26	e	464.27	3.92894	YES	YES
27	b2	514.71	0.00000	NO	YES
28	b1	531.58	0.00000	NO	YES
29	e	535.32	2.93958	YES	YES
30	e	535.32	2.93958	YES	YES
31	e	655.91	122.87991	YES	YES
32	e	655.91	122.87991	YES	YES
33	a1	664.50	128.03722	YES	YES
34	a1	2088.87	467.48695	YES	YES
35	e	2091.47	946.71041	YES	YES
36	e	2091.47	946.71041	YES	YES
37	b2	2113.22	0.00000	NO	YES
38	a1	2163.23	68.50640	YES	YES

39	a1	2287.67	46.65574	YES	YES

$[Mn(CO)_6]^+$

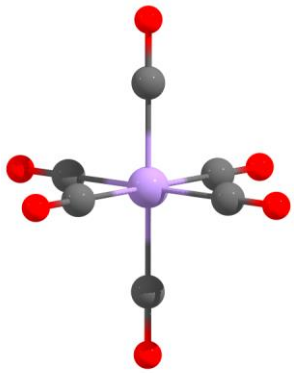

Method: (RI-)BP86(D3BJ)/def2-TZVPP
Symmetry: oh

Cartesian coordinates in Ångström:

Mn	-0.0000000	-0.0000000	-0.0000000
O	0.0000000	3.0225472	-0.0000000
O	0.0000000	-0.0000000	3.0225472
O	-3.0225472	0.0000000	0.0000000
O	-0.0000000	0.0000000	-3.0225472
O	3.0225472	-0.0000000	-0.0000000
O	-0.0000000	-3.0225472	0.0000000
C	-1.8840943	0.0000000	0.0000000
C	1.8840943	-0.0000000	0.0000000
C	0.0000000	-0.0000000	1.8840943
C	-0.0000000	0.0000000	-1.8840943
C	0.0000000	1.8840943	0.0000000
C	-0.0000000	-1.8840943	0.0000000

SCF energy GEOOPT = -1831.435027762 H
ZPE = 133.0 kJ/mol
FREEH energy = 169.91 kJ/mol
FREEH entropy = 0.47634 kJ/mol/K

mode	symmetry	wave number	IR intensity	selection rules	
#		cm**(-1)	km/mol	IR	RAMAN
7	t2u	69.12	0.00000	NO	NO
8	t2u	69.12	0.00000	NO	NO
9	t2u	69.12	0.00000	NO	NO
10	t2g	94.04	0.00000	NO	YES
11	t2g	94.04	0.00000	NO	YES
12	t2g	94.04	0.00000	NO	YES
13	t1u	108.26	1.32337	YES	NO
14	t1u	108.26	1.32337	YES	NO
15	t1u	108.26	1.32337	YES	NO
16	t1g	358.93	0.00000	NO	NO
17	t1g	358.93	0.00000	NO	NO
18	t1g	358.93	0.00000	NO	NO
19	eg	397.86	0.00000	NO	YES
20	eg	397.86	0.00000	NO	YES
21	a1g	400.36	0.00000	NO	YES
22	t1u	432.69	19.87732	YES	NO
23	t1u	432.69	19.87732	YES	NO
24	t1u	432.69	19.87732	YES	NO
25	t2u	512.15	0.00000	NO	NO
26	t2u	512.15	0.00000	NO	NO
27	t2u	512.15	0.00000	NO	NO
28	t2g	534.88	0.00000	NO	YES
29	t2g	534.88	0.00000	NO	YES
30	t2g	534.88	0.00000	NO	YES
31	t1u	671.30	156.91390	YES	NO
32	t1u	671.30	156.91390	YES	NO
33	t1u	671.30	156.91390	YES	NO
34	t1u	2095.22	933.82721	YES	NO
35	t1u	2095.22	933.82721	YES	NO
36	t1u	2095.22	933.82721	YES	NO
37	eg	2115.23	0.00000	NO	YES

38	eg	2115.23	0.00000	NO	YES
39	a1g	2180.15	0.00000	NO	YES

$[Mn(CO)_5(CNH)]^+$

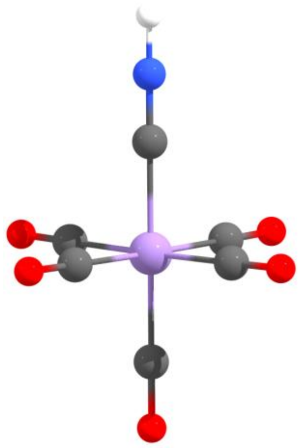

Method: (RI-)BP86(D3BJ)/def2-TZVPP
Symmetry: c4v

Cartesian coordinates in Ångström:

Mn	-0.0000000	0.0000000	0.3167253
O	-2.1328552	2.1328552	0.2819372
O	-2.1328552	-2.1328552	0.2819372
N	0.0000000	0.0000000	-2.7688661
O	2.1328552	2.1328552	0.2819372
O	0.0000000	0.0000000	3.3276196
O	2.1328552	-2.1328552	0.2819372
C	0.0000000	0.0000000	-1.6051302
C	0.0000000	0.0000000	2.1866780
C	-1.3266405	-1.3266405	0.2982381
C	1.3266405	1.3266405	0.2982381
C	-1.3266405	1.3266405	0.2982381
C	1.3266405	-1.3266405	0.2982381
H	0.0000000	0.0000000	-3.7777280

SCF energy GEOOPT = -1811.539022826 H
ZPE = 159.7 kJ/mol
FREEH energy = 198.18 kJ/mol
FREEH entropy = 0.49942 kJ/mol/K

mode #	symmetry	wave number cm**(-1)	IR intensity km/mol	selection rules IR	RAMAN
7	b2	65.61	0.00000	NO	YES
8	e	69.53	1.81501	YES	YES
9	e	69.53	1.81501	YES	YES
10	e	91.84	1.65219	YES	YES
11	e	91.84	1.65219	YES	YES
12	b1	93.93	0.00000	NO	YES
13	a1	106.24	1.63487	YES	YES
14	e	107.33	0.03304	YES	YES
15	e	107.33	0.03304	YES	YES
16	e	350.63	4.58131	YES	YES
17	e	350.63	4.58131	YES	YES
18	a2	361.84	0.00000	NO	NO
19	a1	394.48	3.50172	YES	YES
20	b2	404.89	0.00000	NO	YES
21	a1	405.33	0.00080	YES	YES
22	e	435.02	110.57937	YES	YES
23	e	435.02	110.57937	YES	YES
24	e	437.04	22.90802	YES	YES
25	e	437.04	22.90802	YES	YES
26	a1	440.36	19.12606	YES	YES
27	e	494.32	0.30110	YES	YES
28	e	494.32	0.30110	YES	YES
29	b2	517.63	0.00000	NO	YES
30	e	535.96	0.63347	YES	YES
31	e	535.96	0.63347	YES	YES
32	b1	539.81	0.00000	NO	YES
33	e	666.81	141.90324	YES	YES

34	e	666.81	141.90324	YES	YES
35	a1	678.43	162.68586	YES	YES
36	e	2080.85	1016.07559	YES	YES
37	e	2080.85	1016.07559	YES	YES
38	a1	2088.04	641.95924	YES	YES
39	b2	2102.59	0.00000	NO	YES
40	a1	2119.29	232.47230	YES	YES
41	a1	2174.01	1.30720	YES	YES
42	a1	3675.06	820.30818	YES	YES

$[Mn(CO)_5(CNCH_3)]^+$

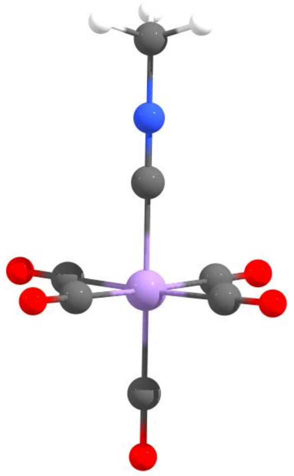

Method: (RI-)BP86(D3BJ)/def2-TZVPP
Symmetry: c1

Cartesian coordinates in Ångström:

Mn	0.8341635	-0.4176685	-0.6885785
H	-2.0758770	1.1934934	3.0395194
H	-3.3225505	0.5901575	1.8997490
H	-2.6523752	2.2422882	1.7035365
O	3.0044474	-1.4865962	-2.4710293
O	1.3044365	2.3824788	-1.7013203
O	-1.2273990	-0.8517950	-2.8450287
O	0.2801117	-3.1777013	0.3903294
O	2.8172077	0.0516054	1.5329649
N	-1.4025996	0.6967922	1.1549366
C	-0.4435819	-0.6890616	-2.0318029
C	2.0704743	-0.1275498	0.6888561
C	1.1306397	1.3204997	-1.3215466
C	0.4939134	-2.1349324	-0.0208962
C	2.1791707	-1.0807425	-1.7935924
C	-0.5653581	0.2771734	0.4636701
C	-2.4248238	1.2115586	2.0002329

SCF energy GEOOPT = -1850.882131507 H
ZPE = 233.9 kJ/mol
FREEH energy = 277.10 kJ/mol
FREEH entropy = 0.56700 kJ/mol/K

mode #	symmetry	wave number cm**(-1)	IR intensity km/mol	selection rules IR	selection rules RAMAN
7	a	14.07	0.00007	YES	YES
8	a	51.05	1.92239	YES	YES
9	a	51.27	1.92481	YES	YES
10	a	63.18	0.00009	YES	YES
11	a	80.67	0.08159	YES	YES
12	a	80.79	0.08238	YES	YES
13	a	93.69	0.00003	YES	YES
14	a	101.60	0.20169	YES	YES
15	a	101.79	0.42785	YES	YES
16	a	101.96	1.00337	YES	YES
17	a	190.00	4.07209	YES	YES
18	a	190.30	4.08101	YES	YES
19	a	324.03	6.24140	YES	YES
20	a	362.50	0.00002	YES	YES
21	a	367.25	0.03194	YES	YES
22	a	367.53	0.03005	YES	YES
23	a	406.97	0.11327	YES	YES
24	a	407.26	0.00037	YES	YES
25	a	439.21	18.99685	YES	YES
26	a	439.23	19.01845	YES	YES
27	a	442.84	13.39917	YES	YES
28	a	492.90	2.68836	YES	YES
29	a	493.15	2.61741	YES	YES

30	a	518.92	0.00040	YES	YES
31	a	538.98	0.44606	YES	YES
32	a	539.16	0.46035	YES	YES
33	a	541.22	0.00025	YES	YES
34	a	666.18	147.52126	YES	YES
35	a	666.33	147.55788	YES	YES
36	a	678.59	171.35839	YES	YES
37	a	959.30	2.32447	YES	YES
38	a	1101.43	0.23221	YES	YES
39	a	1101.53	0.22628	YES	YES
40	a	1400.45	9.86430	YES	YES
41	a	1429.89	15.87348	YES	YES
42	a	1429.94	15.91256	YES	YES
43	a	2073.97	1036.36071	YES	YES
44	a	2073.99	1036.48108	YES	YES
45	a	2080.58	543.22392	YES	YES
46	a	2096.52	0.00836	YES	YES
47	a	2149.09	171.25717	YES	YES
48	a	2258.09	234.64726	YES	YES
49	a	2990.28	0.79402	YES	YES
50	a	3074.90	0.96211	YES	YES
51	a	3075.11	0.96802	YES	YES

$[Mn(CO)_5(CNCF_3)]^+$

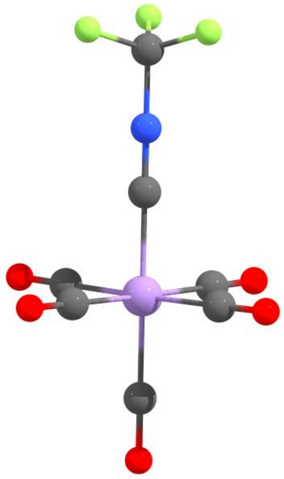

Method: (RI-)BP86(D3BJ)/def2-TZVPP
Symmetry: c1

Cartesian coordinates in Ångström:

Mn	0.8310399	-0.4165880	-0.6872384
F	-1.9826237	1.1834578	3.2567329
F	-3.5063010	0.4486254	1.8723499
F	-2.6899358	2.4619283	1.6308341
O	3.0111943	-1.4887508	-2.4726983
O	1.3194443	2.3786457	-1.7158559
O	-1.2140180	-0.8607028	-2.8623628
O	0.2932224	-3.1890164	0.3781708
O	2.8305183	0.0464067	1.5258347
N	-1.3873973	0.6879111	1.1388435
C	-0.4391257	-0.6940998	-2.0432239
C	2.0784790	-0.1296245	0.6875829
C	1.1381597	1.3215150	-1.3300776
C	0.4994595	-2.1434575	-0.0261613
C	2.1863598	-1.0836464	-1.7976264
C	-0.5455889	0.2661520	0.4442818
C	-2.4228869	1.2112441	2.0006138

SCF energy GEOOPT = -2148.734438311 H
ZPE = 173.2 kJ/mol
FREEH energy = 221.14 kJ/mol
FREEH entropy = 0.62131 kJ/mol/K

mode	symmetry	wave number	IR intensity	selection rules	
#		cm**(-1)	km/mol	IR	RAMAN
7	a	6.95	0.00030	YES	YES
8	a	29.70	0.22188	YES	YES
9	a	30.56	0.21962	YES	YES
10	a	65.72	0.00004	YES	YES
11	a	71.91	0.12698	YES	YES
12	a	72.22	0.13131	YES	YES
13	a	90.33	0.03947	YES	YES
14	a	90.60	0.04163	YES	YES
15	a	93.77	0.00018	YES	YES
16	a	95.21	0.66268	YES	YES
17	a	107.00	0.66892	YES	YES
18	a	107.26	0.66428	YES	YES
19	a	230.18	3.21279	YES	YES
20	a	336.55	0.35651	YES	YES
21	a	336.90	0.35850	YES	YES
22	a	360.49	0.00004	YES	YES
23	a	402.72	0.00093	YES	YES
24	a	403.08	1.12210	YES	YES
25	a	413.00	1.32084	YES	YES
26	a	413.05	1.34627	YES	YES
27	a	429.70	19.74357	YES	YES
28	a	436.73	18.38768	YES	YES
29	a	436.77	18.36370	YES	YES
30	a	494.94	0.88492	YES	YES

31	a	495.12	0.84442	YES	YES
32	a	515.81	0.00017	YES	YES
33	a	533.07	0.07653	YES	YES
34	a	533.24	0.08245	YES	YES
35	a	537.94	0.00012	YES	YES
36	a	589.19	1.33718	YES	YES
37	a	589.35	1.32781	YES	YES
38	a	600.31	15.11786	YES	YES
39	a	664.94	149.03579	YES	YES
40	a	665.09	149.08977	YES	YES
41	a	674.29	275.64193	YES	YES
42	a	829.64	0.02186	YES	YES
43	a	1064.54	911.66901	YES	YES
44	a	1195.94	287.64775	YES	YES
45	a	1196.22	287.69310	YES	YES
46	a	2084.24	978.16584	YES	YES
47	a	2084.26	978.26975	YES	YES
48	a	2093.32	633.95404	YES	YES
49	a	2105.54	0.00040	YES	YES
50	a	2139.96	577.46919	YES	YES
51	a	2208.44	429.14100	YES	YES

$[Mn(CO)_5(CNC_6H_5)]^+$

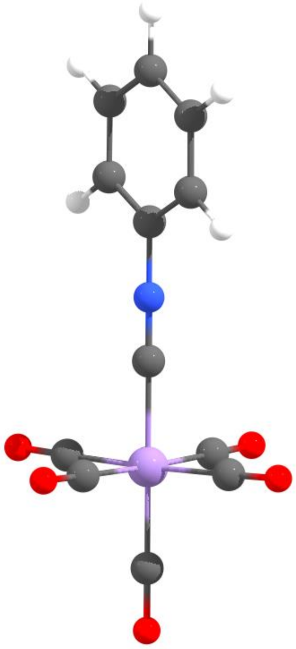

Method: (RI-)BP86(D3BJ)/def2-TZVPP
Symmetry: c2

Cartesian coordinates in Ångström:

Mn	0.0000000	-0.0000000	2.8205973
H	0.0000000	-0.0000000	-5.5293681
H	0.9270767	-1.9473053	-4.2905323
H	0.9290135	-1.9509645	-1.7961649
H	-0.9290135	1.9509645	-1.7961649
H	-0.9270767	1.9473053	-4.2905323
O	0.0000000	0.0000000	5.8246171
O	0.5787376	-2.9562438	2.7483225
O	-2.9575002	-0.5796046	2.7477037
O	-0.5787376	2.9562438	2.7483225
O	2.9575002	0.5796046	2.7477037
N	-0.0000000	0.0000000	-0.2886646
C	-1.8381067	-0.3602371	2.7828856
C	1.8381067	0.3602371	2.7828856
C	0.3593477	-1.8365708	2.7838013
C	-0.3593477	1.8365708	2.7838013
C	0.0000000	-0.0000000	4.6818190
C	0.0000000	-0.0000000	0.8799179
C	0.0000000	0.0000000	-1.6732502
C	-0.5273777	1.1073438	-2.3543119
C	0.5273777	-1.1073438	-2.3543119
C	0.5218707	-1.0960325	-3.7460936
C	-0.5218707	1.0960325	-3.7460936
C	-0.0000000	0.0000000	-4.4404129

SCF energy GEOOPT = -2042.719762874 H
ZPE = 369.5 kJ/mol
FREEH energy = 420.17 kJ/mol
FREEH entropy = 0.64176 kJ/mol/K

mode #	symmetry	wave number cm**(-1)	IR intensity km/mol	selection rules IR	RAMAN
7	a	2.29	0.00002	YES	YES
8	b	27.89	0.00110	YES	YES
9	b	28.12	0.04248	YES	YES
10	a	61.88	0.00812	YES	YES
11	b	73.49	0.06195	YES	YES
12	b	75.20	0.00284	YES	YES
13	b	90.97	0.05565	YES	YES
14	a	92.22	0.23654	YES	YES
15	b	93.27	0.00591	YES	YES
16	a	93.69	0.00011	YES	YES
17	b	109.42	0.58018	YES	YES

18	b	110.56	1.17339	YES	YES
19	a	221.46	0.95899	YES	YES
20	b	258.52	0.02290	YES	YES
21	b	327.55	0.82363	YES	YES
22	a	362.99	0.00000	YES	YES
23	b	373.86	0.05071	YES	YES
24	a	393.15	0.00002	YES	YES
25	a	406.87	0.82344	YES	YES
26	a	408.82	0.55436	YES	YES
27	b	430.68	11.67267	YES	YES
28	b	436.95	14.11945	YES	YES
29	a	442.65	12.69396	YES	YES
30	b	459.11	8.25011	YES	YES
31	b	473.39	12.31746	YES	YES
32	b	516.03	0.43705	YES	YES
33	b	517.25	5.15117	YES	YES
34	a	518.74	0.06540	YES	YES
35	a	535.64	20.66748	YES	YES
36	b	540.43	0.01985	YES	YES
37	b	540.49	0.27303	YES	YES
38	a	541.44	0.00075	YES	YES
39	b	614.55	0.00583	YES	YES
40	b	663.04	126.15534	YES	YES
41	b	667.52	140.66403	YES	YES
42	b	676.70	34.33073	YES	YES
43	a	677.62	239.29557	YES	YES
44	b	751.36	53.85579	YES	YES
45	a	791.78	9.39959	YES	YES
46	a	814.64	0.00010	YES	YES
47	b	912.39	1.94884	YES	YES
48	a	962.31	0.00007	YES	YES
49	a	994.42	7.86062	YES	YES
50	b	996.15	0.00368	YES	YES
51	a	1021.06	1.39761	YES	YES
52	b	1081.34	7.25320	YES	YES
53	a	1158.11	15.61982	YES	YES
54	b	1162.98	0.07567	YES	YES
55	a	1191.61	7.73885	YES	YES
56	b	1305.50	0.00520	YES	YES
57	b	1344.50	3.37716	YES	YES
58	b	1447.97	8.20662	YES	YES
59	a	1470.83	4.33041	YES	YES
60	b	1575.85	3.16462	YES	YES
61	a	1581.81	6.86819	YES	YES
62	b	2071.23	996.12536	YES	YES
63	b	2073.07	1016.99946	YES	YES
64	a	2076.80	640.58378	YES	YES
65	a	2094.96	0.36208	YES	YES
66	a	2144.35	294.89368	YES	YES
67	a	2212.13	69.92060	YES	YES
68	a	3121.70	0.04421	YES	YES
69	b	3128.87	0.11510	YES	YES
70	a	3134.40	0.37382	YES	YES
71	b	3140.99	0.24612	YES	YES
72	a	3144.90	0.23586	YES	YES

[Mn(CO)$_5$(*p*-CNC$_6$H$_4$F)]$^+$

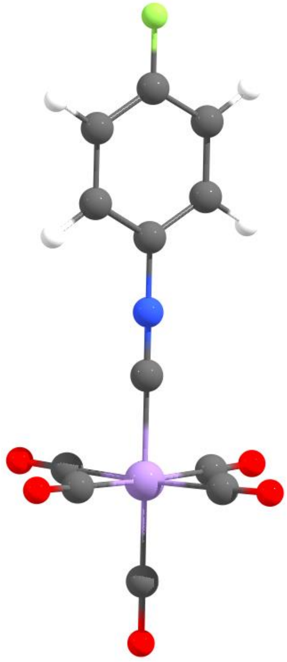

Method: (RI-)BP86(D3BJ)/def2-TZVPP
Symmetry: c1

Cartesian coordinates in Ångström:

Mn	-0.0000061	-0.0000041	2.8183743
F	0.0000326	0.0000157	-5.7581896
H	0.9237692	-1.9441679	-4.3153232
H	0.9278593	-1.9528820	-1.8042144
H	-0.9279516	1.9528382	-1.8042143
H	-0.9236262	1.9442362	-4.3153232
O	0.0000532	0.0000225	5.8225242
O	0.5797128	-2.9560807	2.7476102
O	-2.9573454	-0.5810412	2.7452058
O	-0.5797300	2.9560708	2.7475827
O	2.9573305	0.5810311	2.7450864
N	-0.0000448	-0.0000214	-0.2917931
C	-1.8380816	-0.3611228	2.7806854
C	1.8380680	0.3611136	2.7806142
C	0.3599747	-1.8364472	2.7825507
C	-0.3599897	1.8364382	2.7825343
C	0.0000289	0.0000116	4.6797834
C	-0.0000326	-0.0000159	0.8774215
C	-0.0000442	-0.0000209	-1.6734505
C	-0.5262017	1.1071807	-2.3588849
C	0.5261448	-1.1072076	-2.3588849
C	0.5253944	-1.1056820	-3.7477439
C	-0.5253450	1.1057058	-3.7477439
C	0.0000403	0.0000193	-4.4192071

SCF energy GEOOPT = -2142.005952350 H
ZPE = 348.0 kJ/mol
FREEH energy = 400.93 kJ/mol
FREEH entropy = 0.66721 kJ/mol/K

mode	symmetry	wave number	IR intensity	selection rules	
#		cm**(-1)	km/mol	IR	RAMAN
7	a	2.10	0.00001	YES	YES
8	a	24.99	0.08040	YES	YES
9	a	25.61	0.14747	YES	YES
10	a	61.77	0.01107	YES	YES
11	a	69.04	0.30731	YES	YES
12	a	71.56	0.30949	YES	YES
13	a	84.25	0.05837	YES	YES
14	a	88.60	0.17135	YES	YES

15	a	90.14	0.10676	YES	YES
16	a	93.68	0.00041	YES	YES
17	a	103.82	0.39734	YES	YES
18	a	107.63	0.31693	YES	YES
19	a	196.26	1.41083	YES	YES
20	a	208.46	0.15128	YES	YES
21	a	318.41	2.34233	YES	YES
22	a	323.92	0.03909	YES	YES
23	a	362.91	0.00001	YES	YES
24	a	390.01	2.66627	YES	YES
25	a	404.79	0.00186	YES	YES
26	a	406.67	1.04368	YES	YES
27	a	408.84	0.74863	YES	YES
28	a	412.63	1.62080	YES	YES
29	a	435.07	11.11203	YES	YES
30	a	441.28	6.93456	YES	YES
31	a	443.64	16.51669	YES	YES
32	a	463.40	7.88718	YES	YES
33	a	479.08	46.63878	YES	YES
34	a	486.03	9.72125	YES	YES
35	a	515.18	0.24915	YES	YES
36	a	515.35	11.58137	YES	YES
37	a	518.76	0.04175	YES	YES
38	a	539.97	0.00266	YES	YES
39	a	540.25	0.21391	YES	YES
40	a	541.34	0.00130	YES	YES
41	a	634.40	0.03757	YES	YES
42	a	662.37	138.27888	YES	YES
43	a	667.68	141.47484	YES	YES
44	a	675.32	237.59677	YES	YES
45	a	689.45	0.24842	YES	YES
46	a	734.09	9.99976	YES	YES
47	a	791.49	0.00004	YES	YES
48	a	831.60	63.56808	YES	YES
49	a	843.36	10.37913	YES	YES
50	a	923.36	0.02471	YES	YES
51	a	942.19	0.00004	YES	YES
52	a	999.41	2.02649	YES	YES
53	a	1096.61	9.46227	YES	YES
54	a	1143.16	58.55993	YES	YES
55	a	1188.13	2.63181	YES	YES
56	a	1254.92	211.73085	YES	YES
57	a	1279.20	1.80844	YES	YES
58	a	1338.24	1.43022	YES	YES
59	a	1418.51	2.31661	YES	YES
60	a	1485.99	94.06753	YES	YES
61	a	1574.15	1.97171	YES	YES
62	a	1590.63	161.53077	YES	YES
63	a	2071.16	997.67609	YES	YES
64	a	2073.07	1015.84865	YES	YES
65	a	2077.13	639.03469	YES	YES
66	a	2094.94	0.46424	YES	YES
67	a	2144.24	290.57707	YES	YES
68	a	2208.80	46.40483	YES	YES
69	a	3134.98	0.00203	YES	YES
70	a	3136.56	0.48690	YES	YES
71	a	3149.22	4.26917	YES	YES
72	a	3149.56	0.12862	YES	YES

$[Mn(CO)_5(CNC_6F_5)]^+$

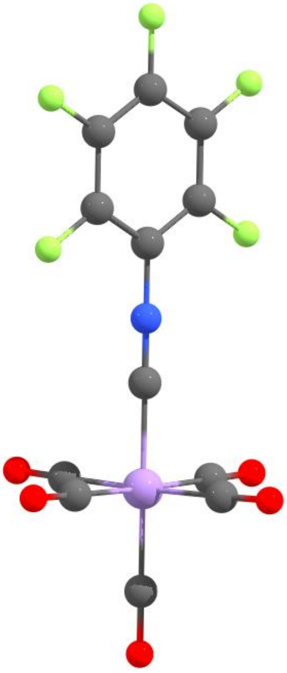

Method: (RI-)BP86(D3BJ)/def2-TZVPP
Symmetry: c2

Cartesian coordinates in Ångström:

Mn	0.0000000	0.0000000	2.8240644
F	0.0000000	0.0000000	-5.7564756
F	0.8652750	-2.2035855	-4.4109485
F	0.8653135	-2.2037165	-1.6690585
F	-0.8653135	2.2037165	-1.6690585
F	-0.8652750	2.2035855	-4.4109485
O	0.0000000	0.0000000	5.8316816
O	0.6907683	-2.9345713	2.7670475
O	-2.9350032	-0.6915102	2.7637475
O	-0.6907683	2.9345713	2.7670475
O	2.9350032	0.6915102	2.7637475
N	0.0000000	0.0000000	-0.2743963
C	-1.8250488	-0.4298326	2.7936146
C	1.8250488	0.4298326	2.7936146
C	0.4295424	-1.8244962	2.7945486
C	-0.4295424	1.8244962	2.7945486
C	0.0000000	0.0000000	4.6897447
C	0.0000000	0.0000000	0.8964818
C	0.0000000	-0.0000000	-1.6400389
C	-0.4453286	1.1339129	-2.3466729
C	0.4453286	-1.1339129	-2.3466729
C	0.4460387	-1.1357928	-3.7391361
C	-0.4460387	1.1357928	-3.7391361
C	0.0000000	0.0000000	-4.4318282

SCF energy GEOOPT = -2539.110474779 H
ZPE = 264.3 kJ/mol
FREEH energy = 324.82 kJ/mol
FREEH entropy = 0.68830 kJ/mol/K

mode #	symmetry	wave number cm**(-1)	IR intensity km/mol	selection rules IR	RAMAN
8	b	19.23	0.04117	YES	YES
9	b	23.95	0.08059	YES	YES
10	b	57.21	0.00404	YES	YES
11	b	60.05	0.00004	YES	YES
12	a	63.08	0.00563	YES	YES
13	b	81.18	0.05780	YES	YES
14	b	82.77	0.04358	YES	YES
15	a	85.04	0.00053	YES	YES

16	a	93.62	0.00014	YES	YES
17	b	102.65	0.75877	YES	YES
18	b	104.05	0.77121	YES	YES
19	a	126.75	0.00000	YES	YES
20	b	146.51	0.07041	YES	YES
21	a	183.57	0.16597	YES	YES
22	b	189.46	1.10723	YES	YES
23	b	235.66	2.07875	YES	YES
24	b	267.22	0.02918	YES	YES
25	a	267.86	0.37759	YES	YES
26	b	287.45	0.04150	YES	YES
27	b	307.58	1.36626	YES	YES
28	a	328.34	3.40784	YES	YES
29	b	341.85	0.01238	YES	YES
30	a	358.28	0.00000	YES	YES
31	a	361.85	0.00003	YES	YES
32	b	387.47	0.07143	YES	YES
33	b	401.27	0.31547	YES	YES
34	a	405.25	1.37518	YES	YES
35	a	406.39	0.90158	YES	YES
36	b	431.64	7.15316	YES	YES
37	a	436.55	6.37080	YES	YES
38	b	439.41	20.24076	YES	YES
39	b	444.28	11.46163	YES	YES
40	a	454.21	31.37750	YES	YES
41	b	489.53	1.72144	YES	YES
42	b	499.02	0.97416	YES	YES
43	a	517.41	0.01301	YES	YES
44	b	536.81	0.10302	YES	YES
45	b	537.46	0.28327	YES	YES
46	a	539.78	0.00053	YES	YES
47	a	558.65	7.50815	YES	YES
48	a	597.62	11.19817	YES	YES
49	b	630.16	0.09937	YES	YES
50	a	631.76	0.00002	YES	YES
51	b	660.86	140.56476	YES	YES
52	b	666.70	135.97833	YES	YES
53	b	677.05	0.19408	YES	YES
54	a	677.20	287.47557	YES	YES
55	b	775.21	0.00000	YES	YES
56	a	983.61	46.80174	YES	YES
57	b	996.97	188.55685	YES	YES
58	a	1149.26	61.10497	YES	YES
59	b	1154.44	3.44937	YES	YES
60	a	1313.15	30.08773	YES	YES
61	b	1323.40	1.62788	YES	YES
62	a	1422.42	126.87067	YES	YES
63	a	1489.36	374.99436	YES	YES
64	b	1494.36	365.99576	YES	YES
65	b	1585.82	9.52881	YES	YES
66	a	1610.25	44.24081	YES	YES
67	b	2076.42	997.98243	YES	YES
68	b	2077.34	961.39006	YES	YES
69	a	2082.49	642.86176	YES	YES
70	a	2099.18	0.16029	YES	YES
71	a	2144.85	381.49730	YES	YES
72	a	2210.25	113.10983	YES	YES

$[Fe(CO)_5]^{2+}$

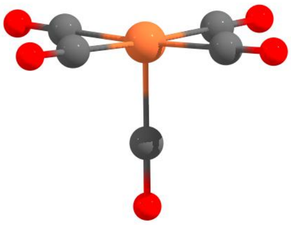

Method: (RI-)BP86(D3BJ)/def2-TZVPP
Symmetry: c4v

Cartesian coordinates in Ångström:

Fe	0.0000000	0.0000000	0.3797215
O	2.1452246	-2.1452246	0.4659621
O	2.1452246	2.1452246	0.4659621
O	0.0000000	0.0000000	-2.5541752
O	-2.1452246	-2.1452246	0.4659621
O	-2.1452246	2.1452246	0.4659621
C	0.0000000	0.0000000	-1.4222089
C	1.3483021	1.3483021	0.4332036
C	-1.3483021	-1.3483021	0.4332036
C	1.3483021	-1.3483021	0.4332036
C	-1.3483021	1.3483021	0.4332036

SCF energy GEOOPT = -1830.233273650 H
ZPE = 109.0 kJ/mol
FREEH energy = 141.20 kJ/mol
FREEH entropy = 0.45162 kJ/mol/K

mode	symmetry	wave number	IR intensity	selection rules	
#		cm**(-1)	km/mol	IR	RAMAN
7	b2	69.89	0.00000	NO	YES
8	e	85.47	0.00002	YES	YES
9	e	85.47	0.00002	YES	YES
10	b1	95.56	0.00000	NO	YES
11	e	108.51	1.60245	YES	YES
12	e	108.51	1.60245	YES	YES
13	a1	111.56	1.94491	YES	YES
14	e	333.04	0.00336	YES	YES
15	e	333.04	0.00336	YES	YES
16	a2	335.07	0.00000	NO	NO
17	b2	353.91	0.00000	NO	YES
18	a1	360.60	2.06776	YES	YES
19	e	371.02	7.32505	YES	YES
20	e	371.02	7.32505	YES	YES
21	a1	439.77	0.67945	YES	YES
22	b2	461.65	0.00000	NO	YES
23	e	491.90	2.76875	YES	YES
24	e	491.90	2.76875	YES	YES
25	b1	501.93	0.00000	NO	YES
26	e	586.96	55.13363	YES	YES
27	e	586.96	55.13363	YES	YES
28	a1	595.23	58.77700	YES	YES
29	a1	2160.43	203.40933	YES	YES
30	e	2180.07	296.60967	YES	YES
31	e	2180.07	296.60967	YES	YES
32	b2	2196.31	0.00000	NO	YES
33	a1	2222.00	1.40655	YES	YES

$[Fe(CO)_5(N_2)]^{2+}$

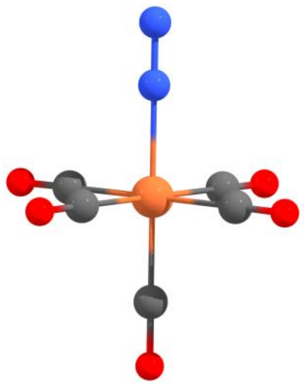

```
Method: (RI-)BP86(D3BJ)/def2-TZVPP
Symmetry: c4v

Cartesian coordinates in Ångström:
Fe   -0.0000000    0.0000000   -0.0023896
O     2.1405786   -2.1405786   -0.0168596
O     2.1405786    2.1405786   -0.0168596
O     0.0000000    0.0000000   -2.9853599
O    -2.1405786   -2.1405786   -0.0168596
N     0.0000000    0.0000000    3.0678932
O    -2.1405786    2.1405786   -0.0168596
C     0.0000000    0.0000000   -1.8553149
N     0.0000000    0.0000000    1.9625900
C     1.3429466    1.3429466   -0.0073436
C    -1.3429466   -1.3429466   -0.0073436
C     1.3429466   -1.3429466   -0.0073436
C    -1.3429466    1.3429466   -0.0073436

SCF energy GEOOPT =  -1939.869224907 H
ZPE =    131.9 kJ/mol
FREEH energy =    170.28 kJ/mol
FREEH entropy =    0.49651 kJ/mol/K
 mode      symmetry      wave number    IR intensity    selection rules
#                         cm**(-1)         km/mol          IR      RAMAN
     7        b2              75.94         0.00000        NO       YES
     8        e               78.09         0.02900        YES      YES
     9        e               78.09         0.02900        YES      YES
    10        b1              95.71         0.00000        NO       YES
    11        e               97.35         0.06061        YES      YES
    12        e               97.35         0.06061        YES      YES
    13        a1             114.21         1.01677        YES      YES
    14        e              117.68         0.78281        YES      YES
    15        e              117.68         0.78281        YES      YES
    16        e              316.09         0.45270        YES      YES
    17        e              316.09         0.45270        YES      YES
    18        a1             319.85         0.07470        YES      YES
    19        a2             338.80         0.00000        NO       NO
    20        b2             362.29         0.00000        NO       YES
    21        a1             367.82         0.22233        YES      YES
    22        e              380.14         9.32130        YES      YES
    23        e              380.14         9.32130        YES      YES
    24        a1             411.65         3.11748        YES      YES
    25        e              438.50         0.55066        YES      YES
    26        e              438.50         0.55066        YES      YES
    27        b2             484.68         0.00000        NO       YES
    28        e              502.87         1.96025        YES      YES
    29        e              502.87         1.96025        YES      YES
    30        b1             505.91         0.00000        NO       YES
    31        e              607.08        85.37905        YES      YES
    32        e              607.08        85.37905        YES      YES
    33        a1             615.48        88.65844        YES      YES
    34        a1            2176.06       192.82865        YES      YES
    35        e             2179.77       358.54631        YES      YES
    36        e             2179.77       358.54631        YES      YES
    37        b2            2196.66         0.00000        NO       YES
```

38	a1	2224.64	12.25037	YES	YES
39	a1	2321.55	6.69203	YES	YES

$[Fe(CO)_6]^{2+}$

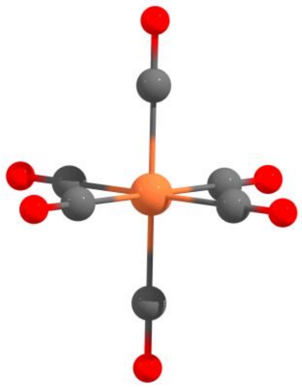

Method: (RI-)BP86(D3BJ)/def2-TZVPP
Symmetry: oh

Cartesian coordinates in Ångström:

Fe	-0.0000000	-0.0000000	0.0000000
O	0.0000000	3.0215798	-0.0000000
O	0.0000000	-0.0000000	3.0215798
O	-3.0215798	0.0000000	-0.0000000
O	-0.0000000	0.0000000	-3.0215798
O	3.0215798	-0.0000000	-0.0000000
O	-0.0000000	-3.0215798	0.0000000
C	-1.8932449	0.0000000	0.0000000
C	1.8932449	-0.0000000	-0.0000000
C	0.0000000	-0.0000000	1.8932449
C	0.0000000	0.0000000	-1.8932449
C	0.0000000	1.8932449	0.0000000
C	-0.0000000	-1.8932449	-0.0000000

SCF energy GEOOPT = -1943.686693534 H
ZPE =   132.7 kJ/mol
FREEH energy =   170.51 kJ/mol
FREEH entropy =   0.47848 kJ/mol/K

mode #	symmetry	wave number cm**(-1)	IR intensity km/mol	selection rules IR	RAMAN
7	t2u	75.32	0.00000	NO	NO
8	t2u	75.32	0.00000	NO	NO
9	t2u	75.32	0.00000	NO	NO
10	t2g	96.84	0.00000	NO	YES
11	t2g	96.84	0.00000	NO	YES
12	t2g	96.84	0.00000	NO	YES
13	t1u	116.14	1.20741	YES	NO
14	t1u	116.14	1.20741	YES	NO
15	t1u	116.14	1.20741	YES	NO
16	t1g	342.97	0.00000	NO	NO
17	t1g	342.97	0.00000	NO	NO
18	t1g	342.97	0.00000	NO	NO
19	eg	367.80	0.00000	NO	YES
20	eg	367.80	0.00000	NO	YES
21	a1g	377.00	0.00000	NO	YES
22	t1u	392.20	9.19516	YES	NO
23	t1u	392.20	9.19516	YES	NO
24	t1u	392.20	9.19516	YES	NO
25	t2u	481.48	0.00000	NO	NO
26	t2u	481.48	0.00000	NO	NO
27	t2u	481.48	0.00000	NO	NO
28	t2g	510.67	0.00000	NO	YES
29	t2g	510.67	0.00000	NO	YES
30	t2g	510.67	0.00000	NO	YES
31	t1u	621.61	113.71436	YES	NO
32	t1u	621.61	113.71436	YES	NO
33	t1u	621.61	113.71436	YES	NO
34	t1u	2179.42	373.59267	YES	NO
35	t1u	2179.42	373.59267	YES	NO
36	t1u	2179.42	373.59267	YES	NO
37	eg	2195.67	0.00000	NO	YES

38	eg	2195.67	0.00000	NO	YES
39	a1g	2232.64	0.00000	NO	YES

$[Fe(CO)_5(CNH)]^{2+}$

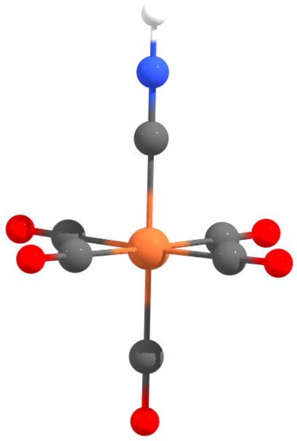

Method: (RI-)BP86(D3BJ)/def2-TZVPP
Symmetry: c4v

Cartesian coordinates in Ångström:

Fe	0.0000000	0.0000000	0.3185911
O	-2.1295491	2.1295491	0.2743963
O	-2.1295491	-2.1295491	0.2743963
N	0.0000000	0.0000000	-2.7552186
O	2.1295491	2.1295491	0.2743963
O	0.0000000	0.0000000	3.3310126
O	2.1295491	-2.1295491	0.2743963
C	0.0000000	0.0000000	-1.6009867
C	0.0000000	0.0000000	2.2012958
C	-1.3309630	-1.3309630	0.2956482
C	1.3309630	1.3309630	0.2956482
C	-1.3309630	1.3309630	0.2956482
C	1.3309630	-1.3309630	0.2956482
H	0.0000000	0.0000000	-3.7748722

SCF energy GEOOPT = -1923.815475968 H
ZPE = 160.9 kJ/mol
FREEH energy = 199.52 kJ/mol
FREEH entropy = 0.49733 kJ/mol/K

mode	symmetry	wave number	IR intensity	selection rules	
#		cm**(-1)	km/mol	IR	RAMAN
7	b2	72.74	0.00000	NO	YES
8	e	75.85	2.04187	YES	YES
9	e	75.85	2.04187	YES	YES
10	e	95.56	2.02180	YES	YES
11	e	95.56	2.02180	YES	YES
12	b1	97.01	0.00000	NO	YES
13	a1	115.34	1.41284	YES	YES
14	e	115.44	0.19104	YES	YES
15	e	115.44	0.19104	YES	YES
16	e	337.75	6.55445	YES	YES
17	e	337.75	6.55445	YES	YES
18	a2	346.48	0.00000	NO	NO
19	a1	374.28	0.08090	YES	YES
20	b2	376.72	0.00000	NO	YES
21	a1	383.63	0.04789	YES	YES
22	e	396.44	17.39471	YES	YES
23	e	396.44	17.39471	YES	YES
24	a1	406.27	8.65988	YES	YES
25	e	468.50	1.51284	YES	YES
26	e	468.50	1.51284	YES	YES
27	b2	487.55	0.00000	NO	YES
28	e	509.26	1.03147	YES	YES
29	e	509.26	1.03147	YES	YES
30	b1	517.62	0.00000	NO	YES
31	e	587.75	87.21784	YES	YES
32	e	587.75	87.21784	YES	YES
33	e	619.44	121.28420	YES	YES

34	e	619.44	121.28420	YES	YES
35	a1	631.40	121.61498	YES	YES
36	e	2168.82	434.01326	YES	YES
37	e	2168.82	434.01326	YES	YES
38	a1	2176.13	263.77995	YES	YES
39	b2	2186.46	0.00000	NO	YES
40	a1	2194.73	38.77774	YES	YES
41	a1	2227.63	1.96001	YES	YES
42	a1	3552.69	912.52671	YES	YES

$[Fe(CO)_5(CNCH_3)]^{2+}$

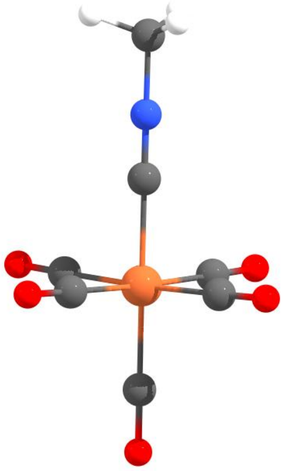

Method: (RI-)BP86(D3BJ)/def2-TZVPP
Symmetry: c1

Cartesian coordinates in Ångström:

Fe	0.8588115	-0.3967386	-0.6738934
H	-2.1468677	1.1231383	2.9919741
H	-3.3610350	0.4988262	1.8163469
H	-2.7180511	2.1729709	1.6438148
O	3.0907332	-1.4070293	-2.4173700
O	1.2718732	2.4204205	-1.6427201
O	-1.1611317	-0.8491953	-2.8562160
O	0.3240502	-3.1600350	0.3871140
O	2.7609543	0.1037345	1.6016415
N	-1.4282684	0.6543462	1.1185237
C	-0.3954756	-0.6821115	-2.0417257
C	2.0532016	-0.0866717	0.7411790
C	1.1240573	1.3587222	-1.2842244
C	0.5319639	-2.1250354	-0.0167152
C	2.2510634	-1.0279497	-1.7620265
C	-0.5725944	0.2579763	0.4468868
C	-2.4832847	1.1446315	1.9474105

SCF energy GEOOPT = -1963.171623019 H
ZPE = 233.7 kJ/mol
FREEH energy = 277.35 kJ/mol
FREEH entropy = 0.56772 kJ/mol/K

mode #	symmetry	wave number cm**(-1)	IR intensity km/mol	selection rules IR	RAMAN
7	a	10.18	0.00013	YES	YES
8	a	55.60	1.89074	YES	YES
9	a	55.70	1.88919	YES	YES
10	a	70.34	0.00008	YES	YES
11	a	85.85	0.07947	YES	YES
12	a	85.94	0.07948	YES	YES
13	a	97.07	0.00001	YES	YES
14	a	108.44	0.08855	YES	YES
15	a	108.50	0.08897	YES	YES
16	a	111.40	0.70770	YES	YES
17	a	212.33	5.66557	YES	YES
18	a	212.58	5.67290	YES	YES
19	a	319.19	0.76688	YES	YES
20	a	347.93	0.00001	YES	YES
21	a	365.37	0.47620	YES	YES
22	a	365.53	0.47605	YES	YES
23	a	380.75	0.00014	YES	YES
24	a	385.47	0.28420	YES	YES
25	a	404.02	10.27935	YES	YES
26	a	404.11	10.31648	YES	YES
27	a	404.86	5.75419	YES	YES
28	a	474.20	0.51197	YES	YES
29	a	474.39	0.49648	YES	YES
30	a	488.89	0.00032	YES	YES
31	a	513.46	0.12901	YES	YES

32	a	513.56	0.12856	YES	YES
33	a	520.51	0.00007	YES	YES
34	a	619.11	106.04518	YES	YES
35	a	619.13	106.05389	YES	YES
36	a	632.79	135.65229	YES	YES
37	a	917.56	0.22506	YES	YES
38	a	1090.35	0.67770	YES	YES
39	a	1090.42	0.66534	YES	YES
40	a	1386.72	0.03102	YES	YES
41	a	1409.88	22.76626	YES	YES
42	a	1409.99	22.82428	YES	YES
43	a	2162.94	458.74887	YES	YES
44	a	2162.96	458.65835	YES	YES
45	a	2170.62	230.50908	YES	YES
46	a	2181.28	0.00563	YES	YES
47	a	2214.02	56.06521	YES	YES
48	a	2293.29	21.25881	YES	YES
49	a	2982.75	48.80700	YES	YES
50	a	3074.31	13.06920	YES	YES
51	a	3074.55	13.11223	YES	YES

$[Fe(CO)_5(CNCF_3)]^{2+}$

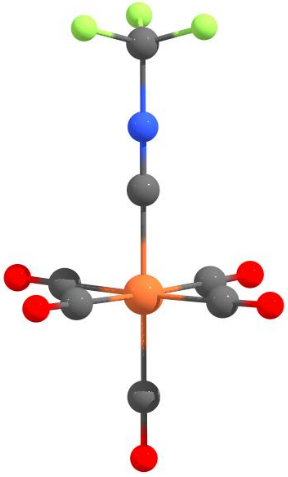

Method: (RI-)BP86(D3BJ)/def2-TZVPP
Symmetry: c1

Cartesian coordinates in Ångström:

Fe	0.8859417	-0.2992569	-0.7116022
F	-2.1110054	0.8304707	3.3020706
F	-3.5338782	-0.0097187	1.8745765
F	-2.9511471	2.0894787	1.7282182
O	3.1506902	-1.0806671	-2.5376985
O	1.1050855	2.5767687	-1.5772084
O	-1.1240795	-0.8042842	-2.8962724
O	0.5843244	-3.1460715	0.2217318
O	2.8126148	0.2330899	1.5408305
N	-1.4325141	0.4917867	1.1541963
C	-0.3650241	-0.6163449	-2.0809131
C	2.0946387	0.0320140	0.6920899
C	1.0275213	1.4963226	-1.2564372
C	0.7024649	-2.0798084	-0.1324197
C	2.3013240	-0.7871833	-1.8528059
C	-0.5588793	0.1956212	0.4518831
C	-2.5880778	0.8777826	2.0797606

SCF energy GEOOPT = -2261.009360426 H
ZPE = 173.0 kJ/mol
FREEH energy = 221.74 kJ/mol
FREEH entropy = 0.62440 kJ/mol/K

mode #	symmetry	wave number cm**(-1)	IR intensity km/mol	selection rules IR	RAMAN
7	a	3.74	0.00008	YES	YES
8	a	35.32	0.43807	YES	YES
9	a	36.02	0.43552	YES	YES
10	a	71.87	0.00003	YES	YES
11	a	80.89	0.09033	YES	YES
12	a	81.17	0.09255	YES	YES
13	a	97.05	0.00044	YES	YES
14	a	97.58	0.10186	YES	YES
15	a	97.93	0.10912	YES	YES
16	a	102.52	0.17736	YES	YES
17	a	117.44	0.50209	YES	YES
18	a	117.74	0.49667	YES	YES
19	a	205.37	5.58972	YES	YES
20	a	321.48	0.83005	YES	YES
21	a	321.81	0.83167	YES	YES
22	a	346.68	0.00003	YES	YES
23	a	377.33	0.00028	YES	YES
24	a	381.99	0.92026	YES	YES
25	a	392.34	7.89528	YES	YES
26	a	392.45	7.88513	YES	YES
27	a	395.22	10.55920	YES	YES
28	a	418.57	3.48859	YES	YES
29	a	418.62	3.47451	YES	YES
30	a	473.70	0.02215	YES	YES

31	a	473.85	0.02161	YES	YES
32	a	487.41	0.00006	YES	YES
33	a	510.15	0.00166	YES	YES
34	a	510.33	0.00195	YES	YES
35	a	518.03	0.00053	YES	YES
36	a	552.69	59.67434	YES	YES
37	a	588.97	0.10594	YES	YES
38	a	589.06	0.11141	YES	YES
39	a	619.47	110.38228	YES	YES
40	a	619.66	110.33905	YES	YES
41	a	634.85	152.39111	YES	YES
42	a	757.09	292.82101	YES	YES
43	a	970.67	568.19322	YES	YES
44	a	1270.83	297.17488	YES	YES
45	a	1271.68	297.34661	YES	YES
46	a	2167.55	433.18708	YES	YES
47	a	2167.58	433.00905	YES	YES
48	a	2175.81	255.04210	YES	YES
49	a	2185.32	0.00445	YES	YES
50	a	2210.89	157.18003	YES	YES
51	a	2256.94	186.28013	YES	YES

$[Fe(CO)_5(CNC_6H_5)]^{2+}$

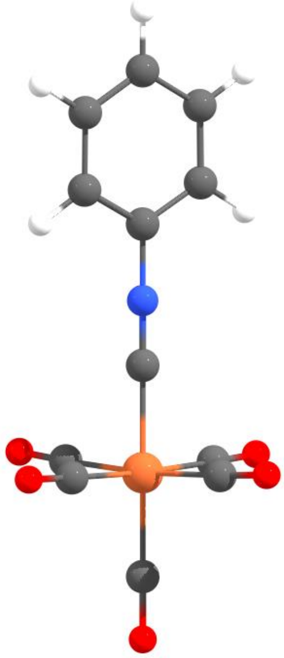

Method: (RI-)BP86(D3BJ)/def2-TZVPP
Symmetry: c1

Cartesian coordinates in Ångström:

Fe	0.0000100	0.0000047	2.8251068
H	0.0003468	0.0001653	-5.4980926
H	0.9281284	-1.9501031	-4.2652779
H	0.9330441	-1.9609666	-1.7733557
H	-0.9333939	1.9608000	-1.7733553
H	-0.9281424	1.9500968	-4.2652777
O	0.0000703	0.0000338	5.8278774
O	0.5737053	-2.9455734	2.7197639
O	-2.9443097	-0.5805325	2.6573693
O	-0.5736885	2.9455810	2.7197304
O	2.9443232	0.5805390	2.6572436
N	-0.0000565	-0.0000271	-0.2739594
C	-1.8379621	-0.3619259	2.7433753
C	1.8379792	0.3619337	2.7432968
C	0.3580779	-1.8365318	2.7724763
C	-0.3580599	1.8365402	2.7724554
C	0.0000476	0.0000229	4.6963874
C	-0.0000265	-0.0000128	0.8924643
C	-0.0000817	-0.0000390	-1.6508501
C	-0.5309511	1.1156524	-2.3287214
C	0.5307930	-1.1157277	-2.3287216
C	0.5237520	-1.1006467	-3.7173033
C	-0.5237214	1.1006616	-3.7173032
C	0.0001157	0.0000552	-4.4088186

SCF energy GEOOPT = -2155.021736022 H
ZPE = 368.6 kJ/mol
FREEH energy = 420.23 kJ/mol
FREEH entropy = 0.64659 kJ/mol/K

mode	symmetry	wave number	IR intensity	selection rules	
#		cm**(-1)	km/mol	IR	RAMAN
7	a	3.81	0.00030	YES	YES
8	a	22.97	0.01497	YES	YES
9	a	31.59	0.00065	YES	YES
10	a	67.38	0.18393	YES	YES
11	a	80.56	0.03504	YES	YES
12	a	81.42	0.01837	YES	YES
13	a	94.68	0.12539	YES	YES
14	a	95.74	0.25604	YES	YES

15	a	97.19	0.00670	YES	YES
16	a	100.56	0.62438	YES	YES
17	a	113.49	1.68706	YES	YES
18	a	116.76	0.68946	YES	YES
19	a	221.42	0.28948	YES	YES
20	a	234.09	0.38841	YES	YES
21	a	314.52	1.79730	YES	YES
22	a	345.62	1.13031	YES	YES
23	a	349.21	0.00014	YES	YES
24	a	382.50	0.00022	YES	YES
25	a	384.18	0.04971	YES	YES
26	a	388.87	3.20561	YES	YES
27	a	389.36	7.00918	YES	YES
28	a	400.11	8.33375	YES	YES
29	a	407.60	5.31179	YES	YES
30	a	430.30	5.00843	YES	YES
31	a	441.49	1.90101	YES	YES
32	a	487.22	0.57475	YES	YES
33	a	501.79	3.02851	YES	YES
34	a	509.56	0.16580	YES	YES
35	a	515.23	2.31777	YES	YES
36	a	522.26	0.05837	YES	YES
37	a	528.73	0.02706	YES	YES
38	a	536.70	2.38456	YES	YES
39	a	607.16	89.03845	YES	YES
40	a	609.42	4.77849	YES	YES
41	a	622.14	99.27700	YES	YES
42	a	633.35	233.05531	YES	YES
43	a	657.75	19.59217	YES	YES
44	a	748.83	56.16549	YES	YES
45	a	788.73	0.51812	YES	YES
46	a	802.04	0.00058	YES	YES
47	a	920.26	0.92283	YES	YES
48	a	971.23	0.00017	YES	YES
49	a	989.04	19.70352	YES	YES
50	a	1007.64	0.16624	YES	YES
51	a	1014.94	0.04818	YES	YES
52	a	1086.70	7.47837	YES	YES
53	a	1150.81	111.56375	YES	YES
54	a	1170.32	0.31624	YES	YES
55	a	1189.24	24.94216	YES	YES
56	a	1306.19	0.11648	YES	YES
57	a	1352.00	7.19021	YES	YES
58	a	1447.12	15.69144	YES	YES
59	a	1455.76	3.92457	YES	YES
60	a	1558.31	2.02789	YES	YES
61	a	1566.90	126.85557	YES	YES
62	a	2156.38	466.50462	YES	YES
63	a	2157.01	470.88868	YES	YES
64	a	2163.38	247.10331	YES	YES
65	a	2175.87	0.08701	YES	YES
66	a	2206.94	14.16072	YES	YES
67	a	2218.65	321.66808	YES	YES
68	a	3127.13	0.09617	YES	YES
69	a	3132.69	0.40706	YES	YES
70	a	3136.80	2.72557	YES	YES
71	a	3143.61	3.13936	YES	YES
72	a	3147.28	3.50581	YES	YES

[Fe(CO)$_5$(*p*-CNC$_6$H$_4$F)]$^{2+}$

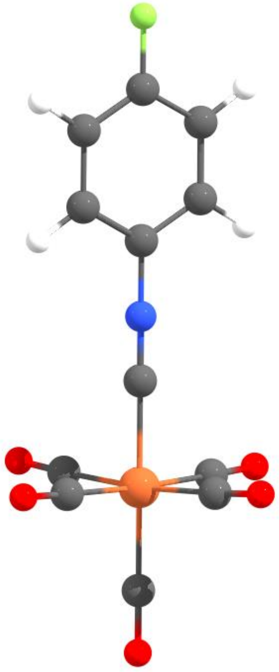

Method: (RI-)BP86(D3BJ)/def2-TZVPP
Symmetry: c1

Cartesian coordinates in Ångström:

Fe	0.0000431	0.0000182	2.8238597
F	-0.0002376	-0.0001089	-5.7134749
H	0.9264502	-1.9484013	-4.2881217
H	0.9335889	-1.9623917	-1.7812353
H	-0.9334533	1.9624535	-1.7812301
H	-0.9267069	1.9482835	-4.2881164
O	-0.0002438	-0.0001134	5.8261744
O	0.5733154	-2.9452882	2.7214059
O	-2.9434256	-0.5814335	2.6433715
O	-0.5731833	2.9453391	2.7215461
O	2.9435456	0.5814931	2.6439595
N	0.0001541	0.0000704	-0.2774280
C	-1.8375773	-0.3624226	2.7363578
C	1.8376792	0.3624710	2.7367058
C	0.3579107	-1.8360547	2.7727955
C	-0.3577984	1.8360991	2.7728781
C	-0.0001243	-0.0000583	4.6946425
C	0.0001595	0.0000729	0.8904703
C	0.0000667	0.0000303	-1.6492747
C	-0.5305188	1.1155519	-2.3334259
C	0.5305526	-1.1155370	-2.3334288
C	0.5285037	-1.1114315	-3.7170302
C	-0.5286092	1.1113840	-3.7170272
C	-0.0000810	-0.0000362	-4.3893740

SCF energy GEOOPT = -2254.307183637 H
ZPE = 347.0 kJ/mol
FREEH energy = 401.03 kJ/mol
FREEH entropy = 0.67227 kJ/mol/K

mode	symmetry	wave number	IR intensity	selection rules	
#		cm**(-1)	km/mol	IR	RAMAN
7	a	2.72	0.00093	YES	YES
8	a	14.81	0.10784	YES	YES
9	a	29.36	0.19279	YES	YES
10	a	67.10	0.31893	YES	YES
11	a	70.07	0.03815	YES	YES
12	a	79.08	0.15335	YES	YES
13	a	84.48	0.00390	YES	YES
14	a	91.93	0.04896	YES	YES
15	a	97.17	0.02972	YES	YES
16	a	98.19	1.37963	YES	YES

17	a	106.99	0.23727	YES	YES
18	a	114.55	0.32085	YES	YES
19	a	189.93	3.34963	YES	YES
20	a	208.84	0.90412	YES	YES
21	a	256.80	0.44838	YES	YES
22	a	308.04	3.80995	YES	YES
23	a	349.22	0.00045	YES	YES
24	a	372.36	6.76638	YES	YES
25	a	383.85	0.04273	YES	YES
26	a	389.15	4.04406	YES	YES
27	a	390.93	7.88953	YES	YES
28	a	394.37	0.00678	YES	YES
29	a	406.19	2.16556	YES	YES
30	a	407.01	4.14812	YES	YES
31	a	424.48	5.31547	YES	YES
32	a	433.87	0.17260	YES	YES
33	a	445.69	2.76273	YES	YES
34	a	480.98	18.16726	YES	YES
35	a	486.77	0.24926	YES	YES
36	a	505.31	4.50166	YES	YES
37	a	509.86	1.30273	YES	YES
38	a	513.86	8.24505	YES	YES
39	a	522.21	0.07953	YES	YES
40	a	526.62	0.10985	YES	YES
41	a	603.91	84.86285	YES	YES
42	a	620.42	85.61251	YES	YES
43	a	631.15	255.52988	YES	YES
44	a	632.42	17.25854	YES	YES
45	a	682.23	1.14589	YES	YES
46	a	736.12	0.00011	YES	YES
47	a	783.77	0.00082	YES	YES
48	a	835.05	64.92229	YES	YES
49	a	841.70	0.07593	YES	YES
50	a	928.09	0.40029	YES	YES
51	a	948.96	0.00000	YES	YES
52	a	990.61	0.34960	YES	YES
53	a	1103.97	9.15005	YES	YES
54	a	1142.36	160.68580	YES	YES
55	a	1188.45	48.05708	YES	YES
56	a	1280.36	2.99105	YES	YES
57	a	1280.95	280.35482	YES	YES
58	a	1341.05	2.18304	YES	YES
59	a	1431.63	7.75469	YES	YES
60	a	1476.12	40.04415	YES	YES
61	a	1548.50	4.02334	YES	YES
62	a	1581.51	479.78509	YES	YES
63	a	2154.79	471.26938	YES	YES
64	a	2156.29	474.57131	YES	YES
65	a	2162.88	236.62940	YES	YES
66	a	2174.92	0.56474	YES	YES
67	a	2203.89	10.62146	YES	YES
68	a	2212.68	428.03181	YES	YES
69	a	3135.55	3.45452	YES	YES
70	a	3137.08	1.05879	YES	YES
71	a	3148.43	18.87248	YES	YES
72	a	3148.80	3.67290	YES	YES

$[Fe(CO)_5(CNC_6F_5)]^{2+}$

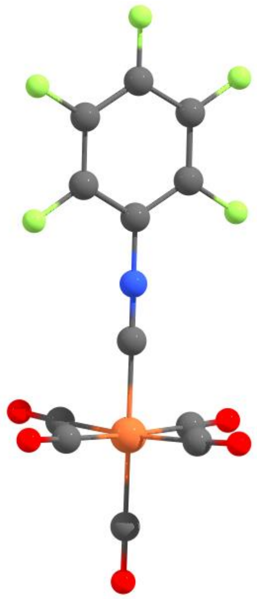

Method: (RI-)BP86(D3BJ)/def2-TZVPP
Symmetry: c1

Cartesian coordinates in Ångström:

Fe	2.2296633	-0.5580841	-1.6485436
F	-4.5116366	1.1200297	3.3304227
F	-3.0170055	3.1803209	2.3886570
F	-0.8423166	2.6472144	0.7873443
F	-1.7312462	-1.9899711	1.1257262
F	-3.9066234	-1.4557873	2.7259358
O	4.5883054	-1.1669727	-3.4074699
O	2.1765855	2.3104860	-2.5430095
O	0.2508722	-1.2536550	-3.8030218
O	2.1264170	-3.3874383	-0.6403479
O	3.9723565	0.2025246	0.6814044
N	-0.2047087	0.0612002	0.1579268
C	1.0112927	-0.9971698	-3.0063988
C	3.3346866	-0.0878292	-0.2060921
C	2.2064443	1.2295421	-2.2129592
C	2.1748220	-2.3260617	-1.0268796
C	3.7004483	-0.9374176	-2.7452708
C	0.7158924	-0.1707962	-0.5212523
C	-1.2764248	0.3260132	0.9490111
C	-2.0626950	-0.7415669	1.4459557
C	-1.6009984	1.6663718	1.2697989
C	-2.6985741	1.9366599	2.0781117
C	-3.1608463	-0.4731716	2.2538463
C	-3.4747106	0.8655588	2.5671044

SCF energy GEOOPT = -2651.405333210 H
ZPE = 263.9 kJ/mol
FREEH energy = 325.33 kJ/mol
FREEH entropy = 0.69778 kJ/mol/K

mode #	symmetry	wave number cm**(-1)	IR intensity km/mol	selection rules IR	selection rules RAMAN
8	a	19.79	0.14012	YES	YES
9	a	22.17	0.04619	YES	YES
10	a	58.64	0.34396	YES	YES
11	a	61.50	0.20459	YES	YES
12	a	68.18	0.19835	YES	YES
13	a	83.81	0.07929	YES	YES
14	a	86.80	0.08327	YES	YES
15	a	91.63	2.73989	YES	YES
16	a	97.15	0.00202	YES	YES
17	a	107.17	0.53239	YES	YES
18	a	110.73	0.69948	YES	YES

19	a	124.26	0.00005	YES	YES
20	a	143.07	0.01827	YES	YES
21	a	182.76	2.04663	YES	YES
22	a	187.95	1.06385	YES	YES
23	a	223.12	3.29580	YES	YES
24	a	266.79	0.13006	YES	YES
25	a	267.68	0.01970	YES	YES
26	a	284.25	0.09602	YES	YES
27	a	305.05	2.19308	YES	YES
28	a	311.33	1.24093	YES	YES
29	a	326.50	0.45421	YES	YES
30	a	348.26	0.00041	YES	YES
31	a	354.98	0.00034	YES	YES
32	a	377.89	4.90751	YES	YES
33	a	382.13	0.01315	YES	YES
34	a	386.64	4.07958	YES	YES
35	a	388.13	2.69711	YES	YES
36	a	404.30	4.55960	YES	YES
37	a	408.63	8.85102	YES	YES
38	a	422.36	6.71044	YES	YES
39	a	432.90	0.17150	YES	YES
40	a	438.85	0.15219	YES	YES
41	a	451.47	12.14303	YES	YES
42	a	484.80	0.08830	YES	YES
43	a	486.78	0.27166	YES	YES
44	a	508.91	0.00911	YES	YES
45	a	513.75	0.02586	YES	YES
46	a	520.69	0.06293	YES	YES
47	a	558.34	0.03454	YES	YES
48	a	596.91	5.46809	YES	YES
49	a	604.90	87.76444	YES	YES
50	a	620.83	97.83286	YES	YES
51	a	624.03	0.34132	YES	YES
52	a	630.37	0.00290	YES	YES
53	a	635.98	246.40186	YES	YES
54	a	677.00	0.16736	YES	YES
55	a	780.20	0.04000	YES	YES
56	a	991.82	32.54210	YES	YES
57	a	1004.31	174.90875	YES	YES
58	a	1164.77	54.04934	YES	YES
59	a	1168.22	4.86213	YES	YES
60	a	1329.79	88.63081	YES	YES
61	a	1336.26	2.84644	YES	YES
62	a	1399.43	296.22404	YES	YES
63	a	1485.37	593.79247	YES	YES
64	a	1495.95	380.58470	YES	YES
65	a	1567.59	39.79919	YES	YES
66	a	1608.47	112.10455	YES	YES
67	a	2158.27	455.93420	YES	YES
68	a	2160.51	449.97305	YES	YES
69	a	2166.09	243.45357	YES	YES
70	a	2178.31	0.50666	YES	YES
71	a	2208.39	26.46526	YES	YES
72	a	2222.77	269.10038	YES	YES

N_2

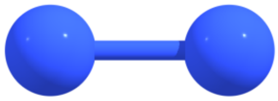

```
Method: (RI-)BP86(D3BJ)/def2-TZVPP
Symmetry: d6h

Cartesian coordinates in Ångström:
N      0.0000000     0.0000000     0.5512924
N      0.0000000     0.0000000    -0.5512924

SCF energy GEOOPT =  -109.5810536899 H
ZPE =   14.06 kJ/mol
FREEH energy =    20.26 kJ/mol
FREEH entropy =   0.19179 kJ/mol/K
mode      symmetry      wave number   IR intensity    selection rules
#                        cm**(-1)        km/mol          IR      RAMAN
     6        a1g          2350.49         0.00000        NO       YES
```

CO

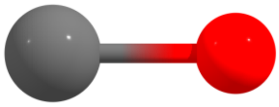

```
Method: (RI-)BP86(D3BJ)/def2-TZVPP
Symmetry: c6v

Cartesian coordinates in Ångström:
O      0.0000000     0.0000000     0.5682110
C      0.0000000     0.0000000    -0.5682110

SCF energy GEOOPT =  -113.3659587682 H
ZPE =   12.71 kJ/mol
FREEH energy =    18.91 kJ/mol
FREEH entropy =   0.19789 kJ/mol/K
mode      symmetry      wave number   IR intensity    selection rules
#                        cm**(-1)        km/mol          IR      RAMAN
     6        a1           2125.72        64.19851        YES      YES
```

CNH

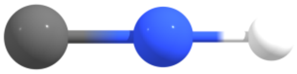

```
Method: (RI-)BP86(D3BJ)/def2-TZVPP
Symmetry: c6v

Cartesian coordinates in Ångström:
N      0.0000000     0.0000000     0.0568059
C      0.0000000     0.0000000    -1.1186975
H      0.0000000     0.0000000     1.0618915

SCF energy GEOOPT =    -93.444729577 H
ZPE =   39.73 kJ/mol
FREEH energy =    47.31 kJ/mol
FREEH entropy =   0.20606 kJ/mol/K
mode      symmetry      wave number   IR intensity    selection rules
#                        cm**(-1)        km/mol          IR      RAMAN
     6        e1            451.44       135.14090        YES      YES
     7        e1            451.44       135.14090        YES      YES
     8        a1           2031.30        65.03921        YES      YES
```

9	a1	3708.27	202.11078	YES	YES

$CNCH_3$

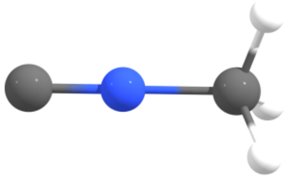

Method: (RI-)BP86(D3BJ)/def2-TZVPP
Symmetry: c3v

Cartesian coordinates in Ångström:

H	-0.5158388	0.8934590	-0.8559526
H	-0.5158388	-0.8934590	-0.8559526
H	1.0316776	0.0000000	-0.8559526
N	0.0000000	0.0000000	0.9366794
C	0.0000000	0.0000000	2.1136113
C	0.0000000	0.0000000	-0.4824330

SCF energy GEOOPT = -132.7781851240 H
ZPE = 115.0 kJ/mol
FREEH energy = 125.12 kJ/mol
FREEH entropy = 0.24672 kJ/mol/K

mode	symmetry	wave number	IR intensity	selection rules	
#		cm**(-1)	km/mol	IR	RAMAN
6	e1	451.44	135.14090	YES	YES
7	e1	451.44	135.14090	YES	YES
8	a1	2031.30	65.03921	YES	YES
9	a1	3708.27	202.11078	YES	YES

$CNCF_3$

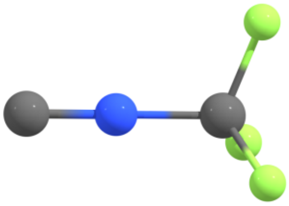

Method: (RI-)BP86(D3BJ)/def2-TZVPP
Symmetry: c3v

Cartesian coordinates in Ångström:

F	-0.6310799	1.0930625	-0.8986502
F	-0.6310799	-1.0930625	-0.8986502
F	1.2621598	0.0000000	-0.8986502
N	-0.0000000	0.0000000	0.9747282
C	0.0000000	0.0000000	2.1570919
C	-0.0000000	0.0000000	-0.4358694

SCF energy GEOOPT = -430.6480766618 H
ZPE = 55.07 kJ/mol
FREEH energy = 69.22 kJ/mol
FREEH entropy = 0.30444 kJ/mol/K

mode	symmetry	wave number	IR intensity	selection rules	
#		cm**(-1)	km/mol	IR	RAMAN
7	e	143.64	1.83699	YES	YES
8	e	143.64	1.83699	YES	YES
9	e	421.39	0.07849	YES	YES
10	e	421.39	0.07849	YES	YES
11	a1	532.66	4.82735	YES	YES
12	e	593.99	2.33806	YES	YES
13	e	593.99	2.33806	YES	YES
14	a1	802.20	0.78590	YES	YES
15	a1	1140.72	477.11285	YES	YES

16	e	1147.07	353.28140	YES	YES
17	e	1147.07	353.28140	YES	YES
18	a1	2119.41	335.82884	YES	YES

CNC_6H_5

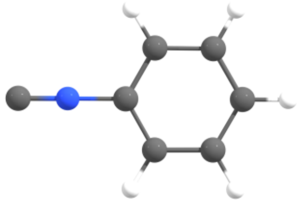

Method: (RI-)BP86(D3BJ)/def2-TZVPP
Symmetry: c2v

Cartesian coordinates in Ångström:

H	0.0000000	0.0000000	-2.8141023
H	2.1552182	0.0000000	-1.5675789
H	2.1514480	0.0000000	0.9291766
H	-2.1514480	0.0000000	0.9291766
H	-2.1552182	0.0000000	-1.5675789
N	0.0000000	0.0000000	2.4443655
C	0.0000000	0.0000000	3.6260167
C	0.0000000	0.0000000	1.0608953
C	-1.2190110	0.0000000	0.3677738
C	1.2190110	0.0000000	0.3677738
C	1.2101795	0.0000000	-1.0255825
C	-1.2101795	0.0000000	-1.0255825
C	0.0000000	0.0000000	-1.7247532

SCF energy GEOOPT = -324.6115437671 H
ZPE = 251.3 kJ/mol
FREEH energy = 268.10 kJ/mol
FREEH entropy = 0.32743 kJ/mol/K

mode	symmetry	wave number	IR intensity	selection rules	
#		cm**(-1)	km/mol	IR	RAMAN
7	b2	139.87	1.73166	YES	YES
8	b1	153.12	3.08842	YES	YES
9	b2	325.81	0.23031	YES	YES
10	a2	399.61	0.00000	NO	YES
11	a1	468.56	0.03672	YES	YES
12	b1	476.56	1.03813	YES	YES
13	b2	511.90	8.66584	YES	YES
14	b1	616.66	0.02042	YES	YES
15	b2	683.47	28.00456	YES	YES
16	b2	750.98	52.24708	YES	YES
17	a1	767.96	4.52837	YES	YES
18	a2	820.55	0.00000	NO	YES
19	b2	903.64	5.58909	YES	YES
20	a2	951.25	0.00000	NO	YES
21	b2	972.18	0.10650	YES	YES
22	a1	995.44	0.14234	YES	YES
23	a1	1022.88	3.60571	YES	YES
24	b1	1074.38	6.89501	YES	YES
25	b1	1153.49	0.00064	YES	YES
26	a1	1158.44	0.89982	YES	YES
27	a1	1193.74	4.61535	YES	YES
28	b1	1300.87	0.46785	YES	YES
29	b1	1338.66	0.84049	YES	YES
30	b1	1445.10	2.68409	YES	YES
31	a1	1477.83	20.07246	YES	YES
32	b1	1577.64	3.90029	YES	YES
33	a1	1593.38	4.25340	YES	YES
34	a1	2115.00	152.84978	YES	YES
35	a1	3107.62	0.02572	YES	YES
36	b1	3116.89	5.90251	YES	YES
37	a1	3127.16	11.03760	YES	YES
38	b1	3136.71	4.53198	YES	YES
39	a1	3138.74	1.15310	YES	YES

p-CNC_6H_4F

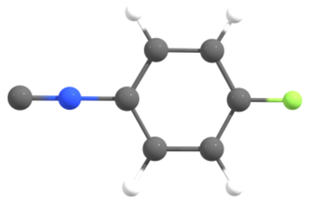

Method: (RI-)BP86(D3BJ)/def2-TZVPP
Symmetry: c2v

Cartesian coordinates in Ångström:

F	0.0000000	0.0000000	-3.0307758
H	2.1495431	0.0000000	-1.5703784
H	2.1524280	0.0000000	0.9451365
H	-2.1524280	0.0000000	0.9451365
H	-2.1495431	0.0000000	-1.5703784
N	0.0000000	0.0000000	2.4649521
C	0.0000000	0.0000000	3.6468149
C	0.0000000	0.0000000	1.0826766
C	-1.2182488	0.0000000	0.3871452
C	1.2182488	0.0000000	0.3871452
C	1.2195538	0.0000000	-1.0051855
C	-1.2195538	0.0000000	-1.0051855
C	0.0000000	0.0000000	-1.6770914

SCF energy GEOOPT = -423.8997215253 H
ZPE = 230.0 kJ/mol
FREEH energy = 248.97 kJ/mol
FREEH entropy = 0.34642 kJ/mol/K

mode	symmetry	wave number	IR intensity	selection rules	
#		cm**(-1)	km/mol	IR	RAMAN
7	b2	105.46	4.60659	YES	YES
8	b1	144.83	5.01400	YES	YES
9	b2	243.24	0.08333	YES	YES
10	b1	378.38	2.49263	YES	YES
11	a2	411.23	0.00000	NO	YES
12	a1	412.15	1.89503	YES	YES
13	b2	414.57	0.09776	YES	YES
14	b1	482.68	0.21375	YES	YES
15	b2	513.01	16.65484	YES	YES
16	b1	636.10	0.08517	YES	YES
17	a1	696.80	25.89350	YES	YES
18	b2	698.53	0.00685	YES	YES
19	a2	792.07	0.00000	NO	YES
20	b2	828.52	68.58879	YES	YES
21	a1	835.03	6.91367	YES	YES
22	b2	921.80	0.50919	YES	YES
23	a2	937.01	0.00000	NO	YES
24	a1	1002.96	1.67291	YES	YES
25	b1	1087.67	10.12388	YES	YES
26	a1	1140.41	12.69661	YES	YES
27	a1	1186.04	32.31100	YES	YES
28	a1	1230.13	95.49945	YES	YES
29	b1	1275.42	0.31597	YES	YES
30	b1	1330.43	1.06476	YES	YES
31	b1	1410.54	0.02826	YES	YES
32	a1	1489.80	132.95590	YES	YES
33	b1	1583.08	1.39086	YES	YES
34	a1	1597.86	32.29296	YES	YES
35	a1	2114.40	139.96773	YES	YES
36	a1	3131.30	2.48100	YES	YES
37	b1	3133.11	0.11038	YES	YES
38	b1	3144.50	0.13798	YES	YES
39	a1	3145.39	0.35056	YES	YES

CNC_6F_5

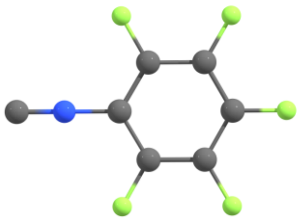

```
Method: (RI-)BP86(D3BJ)/def2-TZVPP
Symmetry: c2v

Cartesian coordinates in Ångström:
F      0.0000000    0.0000000   -3.0381663
F      2.3691708    0.0000000   -1.6785921
F      2.3710519    0.0000000    1.0490334
F     -2.3710519    0.0000000    1.0490334
F     -2.3691708    0.0000000   -1.6785921
N      0.0000000    0.0000000    2.4726014
C      0.0000000    0.0000000    3.6564437
C      0.0000000    0.0000000    1.1047272
C     -1.2097979    0.0000000    0.3884589
C      1.2097979    0.0000000    0.3884589
C      1.2125498    0.0000000   -1.0055983
C     -1.2125498    0.0000000   -1.0055983
C      0.0000000    0.0000000   -1.7022097

SCF energy GEOOPT =  -821.0110521292 H
ZPE =   146.3 kJ/mol
FREEH energy =   175.15 kJ/mol
FREEH entropy =   0.42496 kJ/mol/K
mode      symmetry     wave number   IR intensity    selection rules
#                       cm**(-1)         km/mol          IR      RAMAN
     7        b2               91.86          2.01434       YES      YES
     8        b1              111.73          2.45539       YES      YES
     9        a2              127.48          0.00000       NO       YES
    10        b2              155.99          1.03948       YES      YES
    11        b2              194.49          3.20887       YES      YES
    12        a1              265.13          0.00854       YES      YES
    13        b1              267.47          0.05138       YES      YES
    14        b2              279.88          0.00368       YES      YES
    15        b1              296.03          0.94438       YES      YES
    16        a1              307.32          1.27709       YES      YES
    17        a2              358.30          0.00000       NO       YES
    18        b1              369.27          0.01954       YES      YES
    19        b2              396.05          0.00012       YES      YES
    20        a1              411.39          1.07559       YES      YES
    21        b1              438.82          0.02213       YES      YES
    22        a1              532.87          0.86655       YES      YES
    23        a1              577.67          0.00423       YES      YES
    24        a2              630.78          0.00000       NO       YES
    25        b2              633.32          0.05770       YES      YES
    26        b2              682.60          0.00296       YES      YES
    27        b1              772.68          0.01502       YES      YES
    28        a1              958.63        125.16215       YES      YES
    29        b1              990.60        231.61804       YES      YES
    30        a1             1126.80         32.77193       YES      YES
    31        b1             1140.26          0.50005       YES      YES
    32        a1             1295.39         10.66483       YES      YES
    33        b1             1315.06          0.21237       YES      YES
    34        a1             1428.99          6.62021       YES      YES
    35        b1             1486.99        374.08388       YES      YES
    36        a1             1487.14        259.59357       YES      YES
    37        b1             1592.38          4.02300       YES      YES
    38        a1             1614.48         13.69082       YES      YES
    39        a1             2114.58        187.14012       YES      YES
```

5. References

[1] Borys, A. M. An Illustrated Guide to Schlenk Line Techniques. *Organometallics* **2023**, *42*, 182–196.

[2] M. R. Willcott, M. R. MestRe Nova. *J. Am. Chem. Soc.* **2009**, *131*, 13180.

[3] Dolomanov, O. V.; Bourhis, L. J.; Gildea, R. J.; Howard, J. A. K.; H. Puschmann, H. OLEX2: A complete structure solution, refinement and analysis program. *J. Appl. Cryst.* **2009**, *42*, 339–341.

[4] Sheldrick, G. M. SHELXT – Integrated space-group and crystal-structure determination. *Acta Cryst.* **2015**, *A71*, 3–8.

[5] Sheldrick, G. M. *SHELXL Version 2014/7, Program for Crystal Structure Solution and Refinement*; Göttingen, Germany, 2014.

[6] Sheldrick, G. M. A short history of SHELX. *Acta Cryst.* **2008**, *A64*, 112–122.

[7] Brandenburg, K. Diamond: Crystal and Molecular Structure Visualization http://www.crystalimpact.com/diamond (07/03/2025)

[8] Persistence of Vision Pty. Ltd. Persistence of Vision Raytracer. Ltd., Persistence of Vision Pty. 2004.

[9] a) von Arnim, M.; Ahlrichs, R. Performance of parallel TURBOMOLE for density functional calculations. *J. Comput. Chem.* **1998**, *19*, 1746-1757; b) Treutler, O.; Ahlrichs, R. Efficient molecular numerical integration schemes. *J. Chem. Phys.* **1995**, *102*, 346-354.

[10] Weigend, F.; Ahlrichs, R. Balanced basis sets of split valence, triple zeta valence and quadruple zeta valence quality for H to Rn: Design and assessment of accuracy. *Phys. Chem. Chem. Phys.* **2005**, *7*, 3297-3305.

[11] a) Sierka, M.; Hogekamp, A.; Ahlrichs, R. Fast evaluation of the Coulomb potential for electron densities using multipole accelerated resolution of identity approximation. *J. Chem. Phys.* **2003**, *118*, 9136-9148; b) Weigend, F. Accurate Coulomb-fitting basis sets for H to Rn. *Phys. Chem. Chem. Phys.* **2006**, *8*, 1057-1065; c) Ahlrichs, R. Efficient evaluation of three-center two-electron integrals over Gaussian functions. *Phys. Chem. Chem. Phys.* **2004**, *6*, 5119-5121.

[12] Grimme, S.; Ehrlich, S.; Goerigk, L. Effect of the damping function in dispersion corrected density functional theory. *J. Comput. Chem.* **2011**, *32*, 1456-1465.

[13] Deglmann, P.; Furche, F.; Ahlrichs, R. An efficient implementation of second analytical derivatives for density functional methods. *Chem. Phys. Lett.* **2002**, *362*, 511-518.

[14] a) Klamt, A. Conductor-like Screening Model for Real Solvents: A New Approach to the Quantitative Calculation of Solvation Phenomena. *J. Phys. Chem.* **1995**, *99*, 2224-2235.; b) Klamt, A.; Jonas, V.; Bürger, T.; Lohrenz, J. C. W. Refinement and Parametrization of COSMO-RS. *J. Phys. Chem. A* **1998**, *102*, 5074-5085.

[15] Chemcraft - graphical software for visualization of quantum chemistry computations. Version 1.8, build 682. https://www.chemcraftprog.com (07/03/2025)

[16] Assefa, M. K.; Devera, J. L.; Brathwaite, A. D.; Mosley, J. D.; Duncan, M. A. Vibrational scaling factors for transition metal carbonyls. *Chem. Phys. Lett.* **2015**, *640,* 175–179.
